# Proceedings from the 13th Annual Conference on the Science of Dissemination and Implementation: Virtual. 15-17 December 2020

**DOI:** 10.1186/s13012-021-01110-6

**Published:** 2021-05-20

## Conference website

## I1 Dissemination and Implementation Science in a Dynamic, Diverse, and Interconnected World: Meeting the Urgent Challenges of our Time

### Gila Neta^1^, David A. Chambers^1^, Lisa Simpson^2^

#### ^1^National Cancer Institute, Rockville, MD, USA, ^2^AcademyHealth, Washington, DC, USA

##### **Correspondence:** Gila Neta (Gila.Neta@nih.gov)

In the midst of a global pandemic and heightened national attention to the pervasiveness and impact of systemic racism, the 13^th^ Annual Conference on the Science of Dissemination and Implementation in Health focused on the theme of “Dissemination and Implementation Science in a Dynamic, Diverse, and Interconnected World: Meeting the Urgent Challenges of our Time.” Co-hosted by the National Institutes of Health and AcademyHealth in collaboration with our co-sponsors the Agency for Healthcare Research and Quality (AHRQ), the Patient Centered Outcomes Research Institute (PCORI), the Robert Wood Johnson Foundation (RWJF), and the US Department of Veterans Affairs (VA), the conference was held virtually December 15-17, 2020. While many virtual events attract fewer attendees than their in-person counterparts, the 2020 conference had the highest number of registrants: 1,587. As in prior years, a majority of attendees work in academic settings while 100 were students, 28 were patient scholarship recipients, and 133 participants joined us from 29 low- and middle-income countries.

Over the three-day agenda, we hosted keynote and plenary sessions, concurrent podium and poster sessions, workshops and discussion forums, and multiple networking events. The call for abstracts generated 767 submissions, including individual paper presentations, individual posters, and panel presentations spread across nine thematic tracks. Over two hundred reviewers from multiple disciplines, sectors, settings, and career stages comprehensively assessed the abstracts within each track, coordinated by the track leads. New this year, we piloted a streamlined process for developing concurrent sessions for multiple tracks: We increased the number of reviewers per abstract for more robust scores, which were then used by track leads to assemble thematically relevant concurrent sessions among the most rigorous submissions. Both track leads and reviewers were extremely positive about the new process which we will fully implement going forward. This supplement includes 112 abstracts from the concurrent paper and panel sessions which represents a variety of dissemination and implementation research funded by our conference sponsors as well as other agencies, organizations, and systems. As in previous years, the conference was organized into nine thematic tracks: Behavioral Health, Clinical Care Settings (separated into two tracks: Patient-Level Interventions and System-Level Interventions), Global Dissemination and Implementation Science, Promoting Health Equity and Eliminating Disparities, Health Policy Dissemination and Implementation Science, Prevention and Public Health, and Models, Measures and Methods, and Building the Future of D & I Science: Training, Infrastructure, and Emerging Research Areas. This supplement is organized by these track themes. Slides and recordings of plenary and concurrent sessions (with the agreement of the authors) were posted on the conference website for 90 days after the conference. The additional 402 abstracts from the poster sessions are not included here but can be viewed at https://academyhealth.confex.com/academyhealth/2020di/meetingapp.cgi/ModulePosterSessions/0.

The scientific programming for the conference was driven by the ongoing partnership between the NIH and AcademyHealth with substantial support from a multidisciplinary program planning committee which informed the plenary sessions, recruited key speakers, and helped to develop the topics for workshops and discussion forums. Our keynote and three plenary panels all turned their attention to the challenges of and opportunities in addressing the global health crises and how D&I science can provide direct benefit to ongoing issues of access, implementation and scale-up of evidence-based care. Dr. Ashish Jha opened the conferences with a talk on “The Pandemic Response: Misadventures in Evidence, Communication, and Implementation” highlighting the challenges of effective communication and implementation in the face of rapidly evolving evidence, as well as the limitations in that evidence, particularly regarding systematically documenting health disparities. Dr. Jha highlighted the challenges of actions in the face of limited evidence as well as the challenges of misinformation and disinformation and emphasized the importance of humility particularly among our public health leaders. He concluded with three recommendations for the D&I Science community: invest in data infrastructure; require rigor & quality of pre-prints without slowing down science; and think about who the trusted voices for dissemination and implementation are and build them up.

Building on this rich discussion, the first plenary panel grappled with the dynamism of evidence, focusing on the vast amount of information (and misinformation) being rapidly generated and how it can be best synthesized, disseminated, and acted upon in our implementation studies and practice. The next plenary panel turned attention to unpacking the role of innovation in improving our evidence, interventions, and strategies to maximize equitable implementation and sustainment of health and healthcare practices. Finally, the closing plenary focused on the role of policy in dissemination and implementation science and ways in which policy can provide context and supports to implementation efforts, as well as how evidence can inform policy. For each of these panels, noted leaders brought diverse perspectives to the issues resulting in rich discussions. This diversity was across dimensions including discipline, expertise, gender and race/ethnicity and was intentional in design.

New this year, we explored virtual strategies for fostering dialogue and networking opportunities. One of the most valuable aspects of the virtual platform was the ability for all attendees to engage in conversations through the chat function during the plenary and concurrent sessions. These chats allowed for participants at every level to join in, drive the interaction with presenters, as well as serve as a valuable repository of relevant references and web-based resources which both speakers and participants shared during each of the sessions. To provide additional networking opportunities, we hosted daily coffee chats every morning--each of which featured two D&I experts available for open discussions—as well as virtual yoga and other social gatherings. These networking sessions were hugely popular and well attended, providing attendees with the opportunity to speak directly with some of the leaders in the field. Another tremendous value of the virtual conference was the ability for us to host a record number of participants from LMICs.

As we turn our attention to the next D&I conference in December 2021, whether we are virtual or in-person, we hope to preserve and enhance the valuable lessons learned and connections made during this year’s invigorating conference.

## Behavioral Health

### S-1 Design decisions when using machine learning to optimize behavioral health interventions delivered via smartphones

#### Caroline Figueroa^1^, Adrian Aguilera^2^, Bibhas Chakraborty^3^, Arghavan Modiri^4^, Jai Aggarwal^4^, Nina Deliu^5^, Urmimala Sarkar^6^, Joseph Jay Williams^4^, Courtney Lyles^6^

##### ^1^University of California Berkeley, Berkeley, CA, USA; ^2^University of California, Berkeley, Berkeley, CA, USA; ^3^Duke-National University of Singapore (Duke-NUS) Medical School, Singapore; ^4^University of Toronto, Toronto, ON, Canada; ^5^Rome, Italy; ^6^University of California San Francisco, San Francisco, CA, USA

###### **Correspondence:** Caroline Figueroa (c.a.figueroa@berkeley.edu)

**Background:** Delivering behavioral health interventions via smartphones provides the possibility of personalizing these interventions to individuals’ changing behavior, preferences and needs using reinforcement learning (RL), a subfield of machine learning. Specifically, algorithms for contextual multi-armed bandit (MAB) problems, a reinforcement learning setting in which the algorithm chooses between different treatment options with different reward functions depending on participant’s contexts, might be particularly effective (1). However, the use of these algorithms presents multiple challenges that could impact the effectiveness of them in the real world.

**Methods:** Using thematic analysis, we provide guidelines for decision making about the design of algorithms for mobile health in light of our experience working with health services researchers, clinicians, computer scientists, biostatisticians, and mobile app developers. We use the design process of an MAB for a mobile health study ‘DIAMANTE’ for increasing physical activity in underserved patients with diabetes and depression. Over the 2-year project, we kept track of the research process using Google Docs tools, Whatsapp messenger and video teleconferencing. In year 2 we discussed, categorized, and coded critical challenges. CAF reviewed the initial list of challenges and coded the documents and recordings, which were revised by AA and CRL. Challenges were grouped to create thematic topic process domains.

**Findings:** Fifteen challenges emerged which were divided into 5 major themes. The major challenges were categorized as the following: 1. What type of model to pick for decision making; 2. Choosing the right contextual variables to include in the model; 3. What reward variable to use, 4. How to deal with sparse or missing data in real-time; 5. Efficacy vs. effectiveness/implementation.

**Implications for D&I Research:** Real-world lessons about decision making in the design of reinforcement learning algorithms to improve the effectiveness of behavioral health interventions, not just the final algorithm performance, is essential for advancing this field. Further, different values on efficacy vs. effectiveness/implementation research need to be taken into account, making the tradeoffs in the decisions listed above slightly more complicated. We need collaboration between different disciplines and partnerships to do this work well with real-world patients, in addition to simulations or convenience samples in which generalizability could suffer.

**Primary Funding Source:** Agency for Healthcare Research and Quality

### S-2 Evaluating the implementation of a digital intervention delivering cognitive behavioral therapy for insomnia in primary care

#### Eric Hermes^1^, Robert Rosenheck^1^, Laura Burrone^2^, Carrie Lukens^2^, Greg Dante^1^, Steve Martino^1^

##### ^1^Veterans Health Administration, VA Connecticut Healthcare System, West Haven, CT, USA; ^2^ Veterans Health Administration, VA New England Mental Illness Research, Education, and Clinical Center, West Haven, CT, USA

###### **Correspondence:** Eric Hermes (eric.hermes@yale.edu)

**Background:**

Insomnia is a common and serious condition. Evidence-based self-care digital interventions may mitigate barriers to gold-standard treatment (Cognitive Behavioral Therapy for Insomnia [CBTi]), but are rarely used in healthcare settings. This study evaluated the implementation of a digital CBTi in primary care.

**Methods:**

An implementation strategy and clinical intervention were evaluated using a single cohort design at a Veterans Administration (VA) primary care clinic. The clinical intervention was an evidence-based digital intervention delivering six CBTi sessions in a self-care format. The Replicating Effective Programs implementation strategy was adapted to digital intervention implementation (REP-DI), and included: (1) a local digital intervention coach providing user support; (2) provider/staff training; (3) integrated referral procedures; (4) point of care information products; and (5) a system monitoring implementation and iteratively adapting REP-DI. RE-AIM guided evaluation: Reach (progressive levels of user engagement), Effectiveness (change in clinical outcomes), Adoption (provider/staff referrals), Implementation (fidelity and iterative REP-DI changes), Maintenance (transition to clinical program status).

**Findings:**

Digital CBTi referrals numbered 153, increased from zero before implementation and representing 0.38% of all individuals utilizing primary care. Of referrals, 132 (86.3%) participated in an educational visit with the digital intervention coach. Of those educated, 77 (58.3%) enrolled (intent to treat), while 32 (41.6%) never engaged, 21 (27.3%) engaged in but did not complete the program, and 24 (31.2%) completed. Enrollment was associated with a mean decline of 4.3 (SD=5.6, t=6.4, p<0.001) on the Insomnia Severity Index, and more decline among completers compared to those who did not complete (F=9.6, p<0.001). While 91% of providers/staff were educated, only 26% of eligible providers made a referral. Iterative changes to REP-DI improved some aspects of reach (referral). However, adoption remained stable, and fidelity to coach activities varied.

**Implications for D&I Research:**

This study implemented a digital CBTi in VA primary care using a reproducible implementation strategy (REP-DI), which more than doubled the number of CBTi referrals at the facility, generated a study sample with more complex comorbidities than previous digital CBTi trials, and produced improvement in clinical outcomes. Further research is needed to improve the effectiveness of and fidelity to digital intervention coach and implementation strategy activities.

**Primary Funding Source:** Department of Veterans Affairs

### S-3 Leveraging technology to enhance depression screening and management: Lessons to support the scale and spread of the electronic capture of the PHQ9

#### Elizabeth Austin^1^, Savitha Sangameswaran^1^, Ms. Courtney Segal^1^, Lauren Drake^1^, Denise Chang^2^, Danielle Lavallee^1^

##### ^1^University of Washington, Seattle, WA, USA; ^2^University of Washington Medicine, Seattle, WA, USA

###### **Correspondence:** Elizabeth Austin (austie@uw.edu)

***Background*:** Standardized measurement tools, such as the Patient Health Questionnaire (PHQ), are becoming a mainstay for health systems looking to evaluate depression status and move towards collaborative models of care. Coupled with the increased use of health information technology, there is an opportunity to leverage patient portals to enhance the quality and efficiency of PHQ data capture. Engaging patients outside the care setting using patient portals to collect the PHQ9 can introduce workflow efficiencies and enhance data quality through increased follow-up between clinical encounters. However, little evidence exists to guide the design and implementation of workflows for electronic capture of patient-reported data. This case study reports on learnings from a health system pilot to implement electronic PHQ (ePHQ) via the patient portal for depression screening and management.

***Methods:*** We developed tools for electronic PHQ (ePHQ) data capture through our patient portal, and piloted the ePHQ tools in multiple clinics to support workflows for depression screening and management. Implementation monitoring metrics were reviewed weekly over the course of the one year, and we engaged in PDSA methodology to inform enhancements to technology, workflow, and training. Provider and patient stakeholders were engaged throughout the process to understand their experiences with the ePHQ implementation

***Findings:*** Over one year, 8,596 patients received the ePHQ for screening via the patient portal, of which 1,719 (21%) submitted the ePHQ prior to their scheduled appointment; 367 patients received the ePHQ, for depression management, of which 174 (47%) submitted the ePHQ via the patient portal. On average, 4.2% of screening patients and 9.1% of management patients indicated thoughts of suicide in their ePHQ submissions. Patients raised the importance of communicating the purpose of depression screening in primary care, as well as individualized correspondence for depression management. Feedback from providers called for clearly defined roles and clinical pathways to respond to asynchronous ePHQ response, particularly as the use of telemedicine increased.

***Implications for D&I Research:*** As health systems continue to integrate the electronic patient-reported outcomes into routine care delivery, it will be critical to ensure technology is thoughtfully aligned with pathways for care delivery and clinical decision support.

**Primary Funding Source:** Agency for Healthcare Research and Quality

### S-4 Examining the mechanisms of change for the implementation & sustainment facilitation (ISF) strategy

#### Bryan Garner, Michael Bradshaw, Marianne Kluckman, Stephen Tueller

##### RTI International, Research Triangle Park, NC, USA

###### **Correspondence:** Bryan Garner (bgarner@rti.org)

**Background:** Experimental evidence supports the Implementation & Sustainment Facilitation (ISF) strategy as an effective adjunct to the Addiction Technology Transfer Center (ATTC) strategy for improving implementation of a motivational interviewing-based brief intervention for substance use within HIV service organizations. However, beyond the need to identify effective implementation strategies is the need to understand the mechanisms of change for implementation strategies. This study explored the extent to which changes in implementation climate and readiness were mechanisms of change for the ISF strategy.

**Methods:** Thirty-nine HIV service organizations and their staff (n = 78) were randomized to receive the ATTC strategy or the ATTC+ISF strategy. Implementation climate (i.e., the extent to which implementation was expected, supported, and rewarded) and implementation readiness (i.e., the extent to which staff had the necessary training, knowledge, skill, and time for implementation) were assessed prior to randomization and after the project’s implementation phase. Adjusted multilevel regression analysis was used to examine the extent to which the implementation effectiveness of staff could be predicted by changes in implementation climate and implementation readiness.

**Findings:** The ISF strategy remained a significant predictor of implementation effectiveness in all models. In addition, variation in implementation effectiveness was explained by changes in implementation readiness (β = .14, *p* = .02), but not by changes in implementation climate (β = .05, *p* > .05). Regarding the impact of the ISF strategy on these two putative mediators, the ISF strategy was found to have a significant impact on changes in implementation climate (β = 0.83, *p* = 0.004), but did not quite reach statistical significance regarding changes in implementation readiness (β = 0.65, *p* = 0.09).

**Implications for D&I Research:** The ISF strategy improved implementation climate and implementation effectiveness, yet neither implementation climate nor implementation readiness was able to be supported as a potential mechanism of change. Future research is needed to help understand the mechanism(s) through which the ISF strategy improved implementation effectiveness.

**Primary Funding Source:** National Institutes of Health

### S-5 Influences of inner and outer settings on wraparound implementation fidelity

#### Jonathan Olson^1^, Alya Azman^1^, Philip Benjamin^1^, Kimberly Estep^2^, Kimberly Coviello^2^, Shannon Robshaw^2^, Eric Bruns^3^

##### ^1^University of Washington, Seattle, WA, USA; ^2^University of Maryland, Baltimore, MD, USA; ^3^Department of Psychiatry and Behavioral Sciences, University of Washington School of Medicine, Seattle, WA, USA

###### **Correspondence:** Jonathan Olson (jro10@uw.edu)

**Background:**

Current implementation models underscore the influence of contextual factors both within organizations (inner setting) and in the policy and funding environment (outer setting). However, recent studies within the behavioral sciences have focused more on the role of individuals and inner settings than outer settings, leaving a gap in our understanding of how systems-level factors influence implementation outcomes. The purpose of the current study was to analyze how outer setting administrative structures and inner setting characteristics influence implementation outcomes of the Wraparound Process, a defined and well-supported care coordination model for youth with complex behavioral health needs and their families.

**Methods:**

Wraparound implementation processes were tracked using the Stages of Implementation Completion (SIC). Data were collected from 9 states that represented two distinct outer setting approaches to organizing and financing care coordination for youth: Four employed traditional Community Mental Health Centers (CMHCs), and 5 employed Care Management Entities (CMEs). In addition, fidelity data were collected from a sample of 1174 care coordinators using the Coaching Observation Measure for Effective Teams (COMET). Finally, we examined inner setting workforce development processes by comparing training outcomes across CMHC and CME states within a separate sample of 652 administrators and practitioners from the same 9 states.

**Findings:**

SIC data indicate that compared to states employing a CMHC structure, CME states completed more implementation tasks; and time to completion was faster (14.2 months faster on average). Differences emerged immediately during the pre-implementation phase and widened over time. Furthermore, COMET data indicate that CME states had significantly higher fidelity scores than CMHC states (β= .325, t = 10.115, p < .001); and CMHC staff reported higher levels of competence with the Wraparound model, attended more trainings, but were less likely to make practice changes following a Wraparound training.

**Implications for D&I Research:**

These findings underscore the influence of outer setting, systems-level structures on Wraparound implementation. Compared to CMHC states, CME states were associated with more complete and efficient implementation processes, and had higher fidelity scores. Inner setting findings were mixed. Such results suggest that outer setting policy and fiscal structures should be carefully considered when implementing a multi-system intervention such as Wraparound.

### S-6 Sustainment of cognitive processing therapy: Reach and fidelity across three mental health systems

#### Shannon Wiltsey Stirman^1^, Erin Finley^2^, Jansey Lagdamen^3^, Kera Swanson^4^, Jiyoung Song^5^, Tasoula Masina^6^, Syed Aajmain^7^, Heidi LaBash^3^, Michael Suvak^8^, Vanessa Ramirez^9^, Jeanine Lane^6^, Scott Roesch^10^, Norman Shields^11^, Candice Monson^6^

##### ^1^Veterans Health Administration, National Center for PTSD and Stanford University, Menlo Park, CA, USA; ^2^UT Health San Antonio, San Antonio, TX, USA; ^3^Veterans Health Administration, Palo Alto VA, Palo Alto, CA, USA; ^4^University of California - Irvine, Irvine, CA, USA; ^5^Stanford University, Stanford, CT, USA; ^6^Ryerson University, Toronto, ON, Canada; ^7^Stanford University, Stanford, CA, USA; ^8^Suffolk University, Boston, MA, USA; ^9^UT Health Science San Antonio, San Antonio, TX, USA; ^10^San Diego State University, San Diego, CA, USA; ^11^Royal Canadian Mounted Police, Westmount, QC, Canada

###### **Correspondence:** Shannon Wiltsey Stirman (sws1@stanford.edu)

**Background:**

In the past decade, Cognitive Processing Therapy (CPT) training programs were developed by the VA Healthcare System, Canadian Forces and VA Canada’s Operational Stress Injury Clinics, and the State of Texas. The format of initial training (i.e., training workshops and intensive consultation) was similar, but systems differed in terms of implementation supports and policies, which can impact CPT implementation and sustainability. This study examined CPT reach and fidelity in clinics that had implemented CPT between one and ten years earlier, as part of a randomized controlled trial of implementation strategies to support sustained CPT delivery in these programs.

**Methods:**

This study was conducted in 34 clinics across the three systems. We examined system- and clinic-levels of reach and fidelity at baseline using clinic data, provider surveys (assessing implementation climate, leadership, and attitudes to evidence-based psychotherapies [EBP]), and observation of randomly selected CPT sessions that were recorded by therapists (n=137). Fidelity and adaptations of randomly selected CPT sessions collected during the baseline phase were coded by trained observers.

**Findings:**

Preliminary analyses indicate no significant differences across systems for reach or observer-rated fidelity scores; however the range of adherence to the CPT protocol scores (64-91% of session elements completed) and levels of skill/competence suggest room for improvement (2.59-3.30 on a 0-6 scale). VA clinics scored significantly higher on implementation climate, leadership, and some EBP attitudes than clinics in other systems. We will present analyses that examine associations between potential clinic-level determinants (e.g., implementation climate, leadership, and clinician attitudes perceived characteristics of innovations) and sustainment.

**Implications for D&I Research:**

Findings did not point to system-level differences in reach and fidelity in the years after CPT-training initiatives, but there were differences in potential determinants across systems. Findings also suggest opportunities to more effectively support therapists in the years after training, specifically related to identifying patients most likely to benefit from and increasing providers’ skill and comfort with CPT. These data are of critical importance for systems and organizations in understanding the degree to which EBP delivery is sustained after investment in training and implementation, as well as factors and strategies that may help to optimize sustainment over time.

**Primary Funding Source:** National Institutes of Health

### S-7 Developing a pragmatic measure of clinical supervision techniques that support evidence-based practice implementation: The evidence-based clinical supervision strategy scale

#### Mimi Choy-Brown^1^, Nallely Ramirez^2^, Susan Esp^2^, Nathaniel Williams^2^

##### ^1^University of Minnesota, St.Paul, MN, USA; ^2^Boise State University, Boise, ID, USA

###### **Correspondence:** Mimi Choy-Brown (mchoybro@umn.edu)

**Background:**

Clinical supervision is an attractive entry point for improving evidence-based practice (EBP) implementation in behavioral healthcare because it is nearly universally available in routine care settings and studies show that when supervisors use specific, evidence-based supervision strategies, therapists’ EBP fidelity improves. Currently, very few measures assess clinical supervisors’ use of evidence-based supervision strategies and those that are available have mixed psychometric strength and are not pragmatic. This study developed the Evidence-Based Clinical Supervision Strategies scale (EBCSS)—a pragmatic, theoretically grounded measure of two supervision strategies that are most strongly linked to improved fidelity across EBPs: supervisors’ provision of data-informed feedback, and supervisors’ use of active learning techniques (i.e., modeling and behavioral rehearsal).

**Methods:**

The reliability and validity (factor, convergent, discriminant) of scores on the EBCSS were assessed at baseline in a randomized controlled trial of measurement-based care implementation. The sample included 155 therapists in 21 outpatient behavioral health clinics across 3 states. Confirmatory factor analyses tested the EBCSS’s factor structure. Mixed effects analyses tested associations between scores on the EBCSS and supervisor availability, organizational climate for EBP implementation, and general dimensions of organizational climate. Item difficulty was assessed via item response theory analyses.

**Findings:**

The hypothesized factor structure was confirmed (χ^2^=7.70, *df*=4, *p*=.103, CFI=.993, RMSEA=.077, average standardized factor loading = .80). Coefficient alpha was acceptable for both subscales (feedback, α=.73; active teaching, α=.76), which were correlated *r*=.60, *p*<.001. Robust associations between scores on the EBCSS and climate for EBP implementation (*p*<.05) provided evidence of convergent validity. Small associations with supervisors’ general availability (*p*<.05) and smaller, non-significant associations with general dimensions of organizational climate (*p* > .05) provided evidence of discriminant validity. Scores on the EBCSS also varied in expected directions with therapist and supervision characteristics. IRT analyses indicated the most difficult feedback item was provision of feedback based on supervisor observation of sessions.

**Implications for D&I Research:**

The EBCSS is a promising measure of behavioral health supervisors’ adherence to two essential ingredients of effective clinical supervision for improved EBP fidelity. High quality measures of evidence-based clinical supervision have widespread utility for advancing the field.

**Primary Funding Source:** National Institutes of Health

### S-8 “it’s like riding a bike” – exposure, adherence, and experience with niatx implementation strategies increases treatment encounters for individuals with co-occurring substance use disorders and mental health disorders

#### Jay Ford^1,2^, Deepika Rao^1^, Aaron Gilson^3^, Arveen Kaur^3^, Mark McGovern^4^

##### ^1^School of Pharmacy, University of Wisconsin-Madison, Madison, WI, USA; ^2^University of Wisconsin, Madison, WI, USA; ^3^University of Wisconsin-Madison School of Pharmacy, Madison, WI, USA; ^4^Stanford University School of Medicine, Palo Alto, CA, USA

###### **Correspondence:** Jay Ford (jhfordii@wisc.edu)

**Background:** Although individuals with a co-occurring substance use and mental health disorders (COD) should receive integrated treatment services, only 7% of individuals receive such care. NIATx implementation strategies have been utilized by addiction treatment agencies to improve medication access, reduce appointment wait-times, and increase retention in care. However, its effectiveness at increasing access to treatment encounters for individuals with a COD is unknown.

**Methods:** Using a cluster-randomized waitlist control group design, addiction treatment programs (n=49) were assigned at baseline to Cohort1 (NIATx) or Cohort2 (Waitlist). All program patient admissions 45 days prior to and after each of three Dual Diagnosis Capability in Addiction Treatment (DDCAT) Index assessments were identified. For each admission, substance use disorder (SUD) and mental health (MH) encounters 90-days post-admission and patient demographics were extracted. General linear models examined changes over time for the dependent variables: percent of patients with an SUD, MH or COD encounter. Fixed factors were intervention (Cohort1 vs. Cohort2), period (Baseline, Year1, & Year2) and prior NIATx experience (Yes/No). Per-protocol analysis examined NIATx strategy adherence with and without a prior NIATx experience co-variate.

**Findings:** Percent of patients (n=11,971 admissions) with an SUD encounter increased significantly for Cohort 1 (4%, p=0.02) but decreased by 7.5% (p<0.0001) for Cohort 2 during the active intervention period. Per-protocol analysis (n=8,093 admissions) found that agencies with high NIATx adherence had more patients with an SUD encounter (26.3%, p< 0.001). Alternatively, agencies with low NIATx adherence had a higher percentage of MH encounters (2.8%, p<0.001) or COD encounters (2.5%, p<0.001). Prior NIATx experience eliminated adherence differences for SUD encounters. Agencies with lower adherence and prior experience showed a higher percentage of patients with MH (7.5%, p<0.001) or COD (6.9%, p<0.001) encounters.

**Implications for D&I Research:** NIATx implementation strategies were found to effectively increase access to SUD encounters. Agencies with prior NIATx experience, but low adherence, were able to increase patient encounters. As such, prior experience with NIATx can provide a foundational infrastructure to implement change, regardless of the focus of such change efforts. Future research is needed to replicate these results and understand the role of prior NIATx experience in organizational change.

**Primary Funding Source:** National Institutes of Health

### S-9 Team-level strategies for implementation of a pediatric chronic care model: Strategies, pilot findings, and future directions

#### David Kolko^1^, Ian Bennett^2^, Kimberly Hoagwood^3^, Satish Iyengar^4^, Heather Joseph^4^, Kelly Kelleher^5^, Amy Kilbourne^6^, Elizabeth McGuier^1^, Byron Powell^7^, Maria Silva^8^, Shawna Smith^9^, Renee Turchi^10^, Celeste Liebrecht^6^

##### ^1^University of Pittsburgh School of Medicine, Pittsburgh, PA, USA; ^2^University of Washington, Seattle, WA, USA; ^3^The Child Study Center at NYU Langone Medical Center, New York, NY, New York, NY, USA; ^4^University of Pittsburgh, Pittsburgh, PA, USA; ^5^Nationwide Children's Hospital, Columbus, OH, USA; ^6^Veterans Health Administration, Department of Veteran Affairs, Ann Arbor, MI, USA; ^7^Washington University in St. Louis, St. Louis, MO, USA^8^Allegheny Family Network, Pittsburgh, PA, USA; ^9^University of Michigan Medical School, Ann Arbor, MI, USA; ^10^St. Christopher's Hospital for Children, Philadelphia, PA, USA

###### **Correspondence:** David Kolko (kolkodj@upmc.edu)

**Background:**

Chronic care models (CCMs) can improve the quality of behavioral healthcare in pediatric primary care. Provider teamwork is associated with patient outcomes, and implementation strategies targeting teamwork could enhance CCM uptake and delivery. We will describe pilot work for a team-focused implementation intervention (TEAM) informed by the Enhanced Replicating Effective Programs (REP) Framework and work by Salas and colleagues on team training. TEAM consists of REP strategies integrated with components of team training (e.g., coaching + team self-correction; training in CCM + team cross-training).

**Methods:**

First, we used regression analyses to examine the temporal relationship between baseline practice cooperation and team collaboration interest, post-training team collaboration, and 18-month implementation outcomes (number of patient encounters, barriers to care) in a recent trial of a pediatric CCM for disruptive behavior disorders.

Second, we piloted TEAM with two care teams (n=5) and one leadership team (n=2) in a family medicine practice over a 3-month period. A clinical psychologist conducted bi-weekly team facilitation calls, and care teams incorporated TEAM strategies during brief “huddles.” All participants completed baseline and follow-up ratings of implementation climate (Implementation Climate Scale [ICS]), implementation leadership (Implementation Leadership Scale [ILS]), and teamwork (Primary Care Team Dynamics Scale [PCTDS]).

**Findings:**

In our prior clinical trial, practice cooperation predicted team collaboration interest at 6 months (Coeff=2.53, p<.009), and team collaboration predicted more encounters (Coeff=2.95, p<.001). Team cooperation also predicted fewer barriers to delivering optimal ADHD care 18 months later (Coeff= -0.049, p<.004).

In our TEAM pilot, baseline scores for implementation climate, leadership, and teamwork were close to scale midpoints (ICS = 2.2/4; ILS = 2.4/4; PCTDS = 3.8/5), suggesting room for improvement. Leadership and climate improved at follow-up (ICS = 2.7; ILS = 2.9); there was little change in team dynamics (PCTDS = 3.7). Participants reported TEAM strategies were acceptable, feasible, relevant, and helpful in enhancing CCM delivery.

**Implications for D&I Research:**

These pilot data suggest acceptability and feasibility of TEAM. Improving incorporation of team strategies and leadership strategies in routine practice settings may improve scalability of effective CCM practices and ultimately children’s behavioral health outcomes.

**Primary Funding Source:** National Institutes of Health

### S-10 Determinants of using research in children’s mental health policymaking: Variation by phase of policy process and purpose of research use

#### Jonathan Purtle^1^, Katherine Nelson^2^, Julia Spandorfer^3^, Sarah Horwitz^3^, Mary McKay^4^, Kimberly Hoagwood^3^

##### ^1^Health Management &Policy, Drexel University School of Public Health, Philadelphia, PA, USA; ^2^Health Management and Policy, Drexel University School of Public Health, Philadelphia, PA, USA; ^3^New York University, New York, NY, USA; ^4^Washington University at St Louis, St Louis, MO, USA

###### **Correspondence:** Jonathan Purtle (jpp46@drexel.edu)

**Background:** State policies influence the reach and fidelity of evidence-based mental health treatments (EBTs). D&I strategies that increase the frequency of research use in policymaking can promote policies that support EBTs. This study sought to identify determinants of research use in children’s mental health policymaking and assess how determinants vary by phase of policymaking process and purpose of research use.

**Methods:** A web-based survey of state agency mental health policymakers was conducted between December 2019-February 2020 (n= 224, response rate= 33.7%, 49 states responding, median respondents per state= 4). The dependent variables were seven composite measures of frequency of children’s mental health research use. Three assessed frequency of research use across phases of policy process (i.e., agenda setting, policy development, policy implementation) and four assessed frequency of research use for different purposes (i.e., conceptual to understand an issue, instrumental to inform a decision, political to persuade someone, imposed to satisfy requirements). The independent variables were four composite measures of determinants of using children’s mental health research: agency leadership, agency barriers, research use skills, and dissemination barriers. Adjusted multivariable regression models estimated associations between determinants of research use and each frequency of research use measure.

**Findings:** Determinants of research use varied by phase of policy process and purpose of research use. For example, agency leadership was significantly associated with research use in policy development (β= .27, *p*= .001) and implementation phases (β= .0.20, *p*= .01) but not agenda setting (β=0.12 *p*= .14) and agency leadership was the only determinant significantly associated with imposed research use (β= 0.31, *p*< 0.001). Skills for research use were only associated with research use in the agenda setting phase (β= 0.16, *p*= .04) and were the only determinant associated with political research use (β= 0.18, *p*= .03). Dissemination barriers were the most universal determinant of research use, as they were significantly and inversely associated with frequency of research use across all phases of policy process and conceptual and instrumental research use.

**Implications for D&I Research:** The implementation strategies optimally suited to increase the use of research evidence in mental health policymaking may vary across phases of policy process and purposes of research use.

**Primary Funding Source:** National Institutes of Health

### S-11 The implementation & sustainment facilitation (ISF) strategy: Cost and cost-effectiveness results from a 39-site cluster randomized trial integrating substance use services in AIDS service organizations

#### Jesse Hinde, Rasika Ramanan, Elizabeth Ball, Stephen Tueller, Bryan Garner

##### RTI International, Research Triangle Park, NC, USA

###### Correspondence: Jesse Hinde (jhinde@rti.org)

**Background**: With substance use among individuals living with HIV being both prevalent and problematic, improving the integration of substance use treatment within HIV service settings is urgently needed. The Substance Abuse Treatment to HIV care Project was funded to test an organization-focused strategy called Implementation & Sustainment Facilitation (ISF) as an adjunct to the Addiction Technology Transfer Center (ATTC) strategy. This presentation presents the cost and cost-effectiveness results from this cluster randomized implementation experiment.

**Methods**: Thirty-nine AIDS Service Organizations (ASO) and two brief intervention (BI) staff per ASO (N=78) were randomized to either receive (1) the ATTC strategy (ATTC only) or (2) the ATTC strategy plus the ISF strategy (ATTC+ISF). We estimated costs using primary data on the time spent by staff on the ATTC strategy (training, rated feedback, and group coaching calls), on the ISF strategy (monthly calls and an in-person meeting), and implementing BIs. Salary information was collected via staff surveys. We conducted a cost-effectiveness analysis at the staff level on the number of BIs implemented, the overall MIBI quality scores achieved, and total average patient days abstinent. This study was approved by the RTI International IRB.

**Findings**: Per BI staff costs were $3,133 for the ATTC strategy and $5,905 for the ATTC+ISF strategy, resulting in an incremental cost of $2,722. The standardized difference was 3.65 for BIs delivered and 4.04 for MIBI quality scores achieved, yielding incremental cost effectiveness ratios of $759 and $686, respectively. The incremental cost for BI implementation was $46 per BI staff and patients receiving a BI in the ATTC+ISF condition averaged 57 more days of abstinence at follow-up per BI staff, yielding an incremental cost effectiveness ratio of $50.

**Implications for D&I Research:** The ISF strategy was found to be a cost-effective adjunct to the ATTC’s current state-of-the-art implementation strategy. The current finding is important given that it suggests ISF as a promising strategy to improve the integration of substance use treatment within ASOs. This finding is also of importance given that the ISF intervention may hold promise for helping implement other evidence-based treatments within ASOs or for helping implement other evidence-based treatments within other settings.

**Primary Funding Source:** National Institutes of Health

### S-12 A novel New York State Medicaid reimbursement to incentivize behavioral health outcomes in primary care

#### Amy Jones^1^, Danielle Gadbois^1^, Nathalie Moise^2^, Jay Carruthers^1^

##### ^1^New York State, Office of Mental Health, Albany, NY, USA; ^2^Medicine, Columbia University Irving Medical Center, New York, NY, USA

###### **Correspondence:** Amy Jones (amy.jones@omh.ny.gov)

**Background:** The Collaborative Care Model (CoCm) is the most evidence-based, cost-effective model for integrating behavioral health and primary care. However, sustainable financing poses a major barrier for widespread CoCm adoption. In January 2015, the New York State Office of Mental Health launched the Collaborative Care Medicaid Program (CCMP) to support the sustainable implementation of the CoCm using a novel financing mechanism and quality incentive. We describe fidelity to CCMP strategy here.

**Methods:** All Medicaid primary care practices with the core components of CoCm were eligible to enroll. NYS established a per-member per-month (“case rate”) reimbursement ($112.50) for providing CoCm. In addition, practices licensed by NYS (“Article 28” practices) were also eligible for a quality supplemental payment (QSP). The QSP, an additional 25% reimbursement ($37.50 per patient per month), applied to cases where patients achieved the following targets: (1) a minimum of three months of CoCm services, (2) a clinically significant decrease in their PHQ-9 or GAD-7 (3) or if not improving, a documented change in treatment plan or psychiatric consultation review. NYS also provided ongoing technical assistance calls in addition to audit and feedback, which involved collecting quarterly data on caseload size, improvement rates, and rate of change in treatment or psychiatric consultation from CCMP sites and auditing/cross-checking against Medicaid claims data to monitor performance and ensure compliance.

**Findings:** From 2015 to 2019, 268 clinics enrolled in the CCMP;145 of those were Article 28 clinics. Out of these 145 sites in 2019, 80 (55%) billed the monthly case rate and 36 billed at least one QSP claim. When comparing sites that are billing QSP with those that were only billing the case rate, those billing QSP had an 11% larger average caseload size and billed for a larger percentage of their caseload overall (66% vs. 44%). Sites billing QSP vs. case rate only also had an average of 10% greater improvement rate (52% v. 42%) and greater psychiatric consultation/ change in treatment rates (54% vs. 48%).

**Implications for D&I Research:**We demonstrate feasibility, fidelity to and effectiveness of a hybrid incentive payment strategy for promoting the adoption and scale up of the Collaborative Care model.

### S-13 The role of an intermediary/purveyor organization in behavioral health care

#### Lisa Davis

##### UCLA, Los Angeles, CA, USA

###### **Correspondence:** Lisa Davis (LGDavis@mednet.ucla.edu)

**The Role of an Intermediary/Purveyor Organization in Behavioral Health Care**

**Background:** The quality of public mental health and human services is undermined by the gap between the availability and uptake of evidence-based practices (EBPs). To help spread empirically-supported treatment discoveries, treatment developers, implementation specialists, service system stakeholders and academics have created specialized organizations known as Intermediary/Purveyor Organizations (IPOs). IPOs facilitate dissemination and implementation of EBPs and enhance infrastructure to sustain them in various settings. To date, few studies delineate processes IPOs need to advance their work with public service systems. This study provides a conceptual framework highlighting factors critical to the efficacy of behavioral-health focused IPOs while emphasizing Community-Partnered Participatory principles as a means for IPOs to affect equity and justice, valuing of community expertise, and accountability to community stakeholders as a valued end.

**Methods:** A novel conceptual model was generated using studies examining EBP implementation in community contexts along with lessons learned from developing a university-based IPO serving a public mental health system.

**Findings:** Through conceptual modeling and real-world lessons, we distinguish key components of relationships among academics, implementation practitioners, and community partners in an IPO; and we identify critical functions, roles, and activities of an IO for behavioral health care. Lessons learned include (1) collaboration and policy change strategies critical to IPO success, (2) activities critical to dissemination and implementation (e.g., “change packages”), (3) examples of EBPs the IPO advances. Areas for further inquiry to better understand the contributions of IPOs are also identified.

**Implications for D&I Research:** Key barriers and facilitators in the work of an IPO include the service system political climate and will for change and resource allocation, previous success with innovation initiatives, the perceived needs of the system, available resources and funding, leadership support, and stakeholders’ willingness to work collaboratively. Our model emphasizes the notion that by facilitating knowledge transfer of empirically-supported practices while also attending to the needs and priorities of the local service system, IPOs may be well-situated to support multiple stakeholders in navigating the complex process of overall quality and systems improvement to affect individual and community health and wellbeing.

### S-14 The center for practice innovations: IPO strategies and activities built over a decade

#### Sapana Patel

##### Columbia University Department of Psychiatry, New York, NY, USA

###### **Correspondence:** Sapana Patel (Sapana.Patel@nyspi.columbia.edu)

**Background:** Intermediary/Purveyor organizations (IPO) can provide the infrastructure to support the spread of evidence-based practices while addressing emerging challenges and opportunities faced by service systems. Established in 2008, the Center for Practice Innovations (CPI) at Columbia Psychiatry and the New York State Psychiatric Institute is a mature IPO providing implementation support to the state of New York. New York State (NYS) was an early adopter of evidence-based practices such as Assertive Community Treatment (ACT), a multi-disciplinary team-based approach to serving individuals diagnosed with severe mental illness. ACT teams in NYS are contractually required to follow established standards of care that include a high-fidelity version of ACT. CPI provides training, technical assistance, and strategic planning for the NYS mental health service system to support implementation of ACT and other evidence-based practices.

**Methods:** A review of over a decade of activities by CPI provides key lessons and frameworks critical to successful and sustainability IPO impact within a public mental health system.

**Findings:** As an IPO, CPI has adopted an evolving range activities and strategies for implementation support. The work of CPI is guided by the Consolidated Framework for Implementation Research (CFIR), a practice change model consisting of five domains (i.e., intervention characteristics, outer setting, inner setting, characteristics of the individuals, and the process of implementation). In addition, the stages described in the Replicating Effective Programs framework are used to sequence the adoption of mental health interventions, from pre-implementation to implementation followed by maintenance and future evolution. In collaboration with stakeholders, CPI provides training, consultation and technical assistance to support implementation, identify gaps, and facilitate practice-based research.

**Implications for D&I Research:** CPI operates in a space between policy and practice, synthesizing and translating new knowledge for providers (inner) and policy makers (outer settings). Lessons learned about key barriers and facilitators of IPO work over a decade will be reviewed. Lessons reflect the role for an IPO from the clinic through to the policy level. Key activities that support sustainability of evidence-based practices will be reviewed. Future potential for IPO activities in other service systems will be discussed.

**Primary Funding Source:** New York State

### S-15 The national council for behavioral health

#### Teresa Halliday

##### National Council for Behavioral Health, Washington, DC, USA

###### **Correspondence:** Teresa Halliday (TeresaH@TheNationalCouncil.org)

**Background:**

As the unifying voice of America’s health care organizations that deliver mental health and substance use disorder treatment, the National Council for Behavioral Health (National Council) provides technical assistance and training in support of well-coordinated evidence-based services for vulnerable populations. A nationally-focused Intermediary/Purveyor Organization (IPO), the National Council interrelates with a diverse membership base for implementation support at the local and national levels, translating complex trends and experimental interventions into understandable practical guidance, including implementation of evidence-based practices (EBPs) that can be adopted and replicated in a variety of health care settings. Discussion will include examples of the IPO role in practice transferability across health and related systems, illustrated by delivery of clinical *change packages* – uniform, national standards specific enough for clinicians to implement and measure progress, and generalizable enough to be scaled in different settings.

**Methods:**

The National Council for Behavioral Health has designed, implemented, and evaluated more than 1,500 behavioral health practice improvement initiatives, supporting agencies and clinicians in implementing EBPs on a range of behavioral health and healthcare integration topics. While working with clinicians, the IPO supports agencies in making parallel changes to clinical workflows, business operations, technological infrastructure, and other operational factors that support change in clinical practice.

**Findings:**

The effective implementation of an EBP requires more than clinical staff training and competencies. Agencies will be successful providing EBPs if they ensure that their operational and administrative systems support the new practice. Executive staff must champion the clinical training and lead the non-clinical aspects of the initiative. Policies, procedures and other workflow processes related to billing, supervision, and admission/discharge are just a few of the internal workflow structures that may need to be modified for the EBP to operate successfully. Sustainable implementation requires a continued organizational commitment, ongoing support, and regular training.

**Implications for D&I Research:**

A nationally-focused IPO is uniquely positioned to benefit providers through a centralized pool of expertise fortified by connectivity with myriad institutions supporting education, research, practice, policy, and financing.

**Primary Funding Source:** Multiple

## Building the Future of D&I Science: Training, Infrastructure, and Emerging Research Areas

### S-16 D&I core evaluation and development of strategies for D&I capacity building (Washington University)

#### Elvin Geng^1^, Ana Baumann^2^, Rachel Tabak^3^, Stephanie Mazzucca^4^, Beth Prusaczyk^5^, Aaloke Mody^6^, Todd Combs^7^, Doug Luke^2^

##### ^1^UCSF, San Francisco, CA, USA; ^2^Washington University in St. Louis, St. Louis, MO, USA; ^3^Washington University in Saint Louis, Saint Louis, MO, USA; ^4^Prevention Research Center, Washington University in St. Louis, St. Louis, MO, USA; ^5^Vanderbilt University Medical Center, Nashville, TN, USA; ^6^University of Washington in St. Louis, St. Louis, MO, USA; ^7^Brown School of Social Work, Washington University in St. Louis, St. Louis, MO, USA

###### **Correspondence:** Ana Baumann (abaumannwalker@wustl.edu)

**Background:** We conducted a formative assessment of the penetration of D&I in our university through a survey carried out by the Washington University Clinical and Translational Science Institute and use results to shape emerging strategies to advance the spread of D&I.

**Methods:** In January of 2020, a 10 questions module in the ICTS survey assessed interest in and knowledge of D&I and, for those using ICTS services related to D&I, satisfaction with those services. We used descriptive statistical analyses and simple cross-sectional measures of association to identify factors associated with interest, experience and preferences for D&I.

**Findings:** A total of 595 ICTS customers responded to the survey. The majority of respondents were Assistant Professors (32%), followed by Professors (26%), and Associate Professors (19%). They reported that, overall, (77%) D&I was moderately or very important to their research, while only 7% felt “very familiar” with D&I. Even among 257 (43%) of respondents who considered D&I highly important, only 15% considered themselves familiar with the field. Reasons for lack of familiarity with D&I included not having enough time to learn a new science, and not sure how to integrate the science in their own research. While only 11% of the respondents had used DIRC services, 88% rated their experiences as very good or excellent. When asked who they would look for advice on D&I science, findings show that (a) there is a concentration of mentions identifying senior faculty, (b) a large additional individuals named, many of who may not necessarily identify themselves as D&I experts.

**Implications for D&I Research:** These results indicate even at a University with a strong D&I presence, penetration of the science is limited. To advance reach, our core has developed a strategic approach by: (a) deepening our understanding of consultation to standardize and train a larger cadre of new D&I researches, and (b) developing and tracking different implementation strategies to teach D&I science in our institution. As CTSA cores are constantly facing challenges in increasing capacity building, it is our hope that evaluating the process of teaching D&I will help inform how to effectively increase capacity building in our D&I cores.

**Primary Funding Source:** National Institutes of Health

### S-17 Building capacity to design for dissemination: Lessons learned from the D4D initiative at the UW-Madison CTSA hub.

#### Jane Mahoney^1^, Andrew Quanbeck^2^, Mondira Saha-Muldowney^3^, Sheena Hirschfield^4^

##### ^1^Department of Medicine, University of Wisconsin-Madison, Madison, WI, USA; ^2^Department of Family Medicine and Community Health, University of Wisconsin – Madison, Madison, WI, USA; ^3^University of Wisconsin-Madison, Madison, WI, USA; ^4^University of Wisconsin, Madison, WI, USA

###### **Correspondence:** Jane Mahoney (jm2@medicine.wisc.edu)

**Background:** Designing for dissemination (D4D) is “an active process that helps to ensure that ...health interventions, often evaluated by researchers, are developed in ways that match well with adopters’ needs, assets, and time frames.” (Brownson et al, 2013). The Dissemination and Implementation (D&I) Launchpad™ at the UW-Madison CTSA hub identified that investigators lacked understanding of how to engage adopters in D4D. To address this gap, we developed tools and resources for investigators, and trained practice-based research networks on how to engage adopters in research studies.

**Methods:** With stakeholder input, the D&I Launchpad developed tools to help investigators engage adopters in intervention design. We trained investigators of five practice-based research networks to utilize the tools. We encouraged investigators applying to the CTSA hub’s Clinical and Community Outcomes pilot grant program to consult with the D&I Launchpad and incorporate D4D concepts in their applications. We categorized our recommendations and evaluated investigators’ satisfaction and benefits derived from the consults.

**Findings:** The Launchpad team consulted with 9 of 13 applicants for the 2020 pilot program. Recommendations included: obtain letters of support from potential adopters; discuss potential for scale-up as part of dissemination plan; identify advisory board of potential adopters; and engage potential adopters in intervention design, including identifying barriers and facilitators to adoption and sustainment. Applicants found the consultations moderately (57%) to highly (43%) helpful. Key benefits included “identifying the best D&I opportunities”; “assistance with dissemination plan”; and “information about how to design a study for dissemination and implementation, including which stakeholders to include, type of letter of support to provide, and language to use in the grant.”

**Implications for D&I Research:** We discuss the tools, specific tactics, and overall approach that the D&I Launchpad has developed to maximize utilization of D4D by investigators who work with community-engaged programs and networks in our CTSA hub. Further mixed-methods evaluation of the D4D initiative will identify the extent to which D4D consultations have led to increased engagement with adopters in grant applications, and how grant reviewers’ perceive increased attention to D4D in applications.

**Primary Funding Source:** National Institutes of Health

### S-18 Implementation science scholars program: Bringing implementation science to clinician scholars at the university of Arkansas for medical sciences (UAMS)

#### Geoffrey Curran^1^, Sara Landes^2^, Benjamin Teeter^3^, Jeremy Thomas^4^

##### ^1^Central Arkansas Veterans Healthcare System, Little Rock, AR, USA; ^2^Department of Psychiatry, University of Arkansas for Medical Sciences, Little Rock, AR, USA; ^3^Department of Pharmacy Practice, University of Arkansas for Medical Sciences, Little Rock, AR, USA; ^4^University of Arkansas for Medical Sciences, Little Rock, AR, USA

###### **Correspondence:** Geoffrey Curran (currangeoffreym@uams.edu)

**Background:** The Implementation Science Scholars Program (ISSP) at UAMS is one component of a diverse CTSA-sponsored program to increase capacity in IS. While other programs within the UAMS hub focus on building capacity and activity in extramural research, the ISSP provides IS theory, concepts, methods, and tools to clinician scholars already involved in quality improvement initiatives. The 2-year program provides a series of short courses/trainings, a range of institutional supports (IRB, IT, informatics), salary support, and project mentoring from senior implementation scientists. Applicants propose a project and receive support letters from health system leaders. Health system quality improvement leads share in the review and selection of the scholars and contribute to didactics and project oversight. The goals of the program are—1) expand the scope and rigor of quality improvement within the UAMS CTSA hub, and 2) provide initial training and mentoring to clinician scholars as an invitation to future involvement in IS extramural grants.

**Methods:** The first cohort (5 scholars from 19 applicants) began in January of 2020. A mixed-method evaluation has been initiated. For each short course/training, quantitative evaluations (via REDCap) are completed, as are individual qualitative interviews, which drive content and process revisions. Additional qualitative interviews are conducted every six months to assess scholars’ progress and perceptions of institutional and mentoring support. An external reviewer will attend a yearly symposium of scholar presentations and provide a written evaluation of the program (also based on reviews of course syllabi, and all evaluation data).

**Findings:** Quantitative and qualitative evaluation of the initial didactics (10 modules over 6 weeks) found high levels satisfaction with course content and process; strong evidence of IS knowledge acquisition, methods skills acquisition (e.g., formative evaluation methods), and effective networking with quality and informatics leads; and high confidence in applying IS principles (with mentoring).

**Implications for D&I Research:** High interest and engagement in the ISSP indicates a potentially large pool of clinician scholars interested in learning and applying IS principles. Future evaluation will indicate the program’s effectiveness as a conduit to extramural research activity. The ISSP could serve as a model for other CTSAs and/or other health systems.

**Primary Funding Source:** National Institutes of Health

### S-19 Building research and training capacity for implementation science through CTSAs: Lessons learned from one CTSA hub (Columbia University)

#### Rachel Shelton^1^, Nathalie Moise^2^, Sapana Patel^3^

##### ^1^United States, Columbia's Mailman School of Public Health, New York, NY, USA; ^2^Medicine, Columbia University Irving Medical Center, New York, NY, USA; ^3^Columbia University Department of Psychiatry, New York, NY, USA

###### **Correspondence:** Rachel Shelton (rs3108@columbia.edu)

**Background:** The infrastructure, resources and mission of CTSAs are well-aligned with dissemination and implementation (D&I) science given their common goals of improving processes along the translational continuum to address the well-documented gap between research and practice in health and healthcare. While CTSA Program hubs are well-positioned to help advance and leverage the processes and impact of IS science in this area, not all hubs have well-developed D&I programs, resources, or infrastructure.

**Methods:** Recognizing the potential importance of D&I science within CTSAs and nationally across CTSA hubs, Columbia University’s Irving Institute for Clinical and Translational Research conducted an internal needs assessment in January of 2018 and convened a small faculty Working Group. This Working Group hosted an Implementation Science Symposium with 72 attendees (50% faculty) from 11 institutions/departments/schools across Columbia represented, with the goal of identifying infrastructure, training, and research needs and opportunities form promoting implementation science through the CTSA.

**Findings:** The symposium had 72 attendees (50% faculty) and over half categorized themselves as ‘beginners’ in terms of prior D&I training. Evaluation data identified needs and priorities, ranked as: 1) Introductory and Advanced D&I training; 2) D&I Speaker Seminar Series with national experts; 3) Grant-writing D&I course; 4) Website with D&I Resources; 5) Consultation, including matchmaking with faculty/mentors; and 6) D&I Pilot funding. Over the past 18 months, informed by this needs assessment, we launched an Intervention and Implementation Science pilot award program, monthly campus-wide IS seminar series, piloted a 3-tiered consultation program, and hosted monthly ‘Works-in-Progress’ to provide feedback on grants in development.

**Implications for D&I Research:** Our experience, challenges, and lessons learned in developing and launching and evaluating an Implementation Science Initiative through Columbia’s CTSA can be used as a model for others to develop a centralized infrastructure for enhancing awareness, building research capacity, and providing educational, training, and mentorship opportunities.

**Primary Funding Source:** National Institutes of Health

### S-20 Advancing adaptation research: Evaluating stakeholder-led adaptations to an EBP in routine mental health service settings

#### Kelly Aschbrenner^1^, Dr. Stephen Bartels^2^

##### ^1^Psychiatry, Geisel School of Medicine at Dartmouth College, Nashua, NH, USA; ^2^Massachusetts General Hospital, Harvard Medical School, Boston, MA, USA

###### **Correspondence:** Kelly Aschbrenner (kelly.aschbrenner@dartmouth.edu)

**Background:** Despite imperatives to maintain implementation fidelity when delivering an evidence-based practice (EBP) in routine care, EBPs are often adapted from their original design to fit provider characteristics, organizational contexts, and service settings. However, limited empirical evidence exists on the impact of adaptations that occur in implementing EBPs in real-world practice settings. The purpose of this study was to apply an existing framework for evaluating adaptations to an EBP (InSHAPE) for obesity in persons with serious mental illness in a national implementation in mental health care settings.

**Methods:** We conducted telephone interviews with InSHAPE provider teams at 37 (95%) of 39 study sites during 24-month follow-up of a cluster randomized trial of implementation strategies for InSHAPE at behavioral health organizations. Our team rated adaptations as fidelity-consistent or inconsistent. Multilevel regression models were used to estimate the relationship between adaptations and implementation and participant outcomes.

**Findings:** Of 37 sites interviewed, 28 sites (76%) made adaptations to InSHAPE (*M*=2.1, *SD*=1.3). Sixteen sites (43%) made fidelity-consistent adaptations while 22 (60%) made fidelity-inconsistent adaptations. The number of fidelity-inconsistent adaptations was negatively associated with InSHAPE fidelity scores (*Beta* = - 4.29; *p* <0.05). A greater number of adaptations were associated with significantly higher odds of participant-level cardiovascular risk reduction (*OR*=1.40; *CI* [1.08,1.80]; *p*<0.05). We found a significant positive association between the number of fidelity-inconsistent adaptations and cardiovascular risk reduction (*OR*=1.59; *CI* [1.01, 2.51]; *p*<0.05). This was largely explained by the fidelity-inconsistent adaptation of holding exercise sessions at the mental health agency vs. a fitness facility in the community (a core intervention form) (*OR*=2.52; 95% *CI* [1.11, 5.70]; *p*<0.05).

**Implications for D&I Research:** Findings suggest that adaptations, including those that are fidelity-inconsistent, can be positively associated with improved patient outcomes. This finding advances adaptation research by contributing empirical data to inform the fruitful fidelity-adaptation debate. Developing new methods and strategies for planning and guiding EBP adaptations that provide a potential practical advantage while maintaining the core function of the intervention may be more relevant than ever given the evolving context in which EBPs are being implemented in public health and health care settings.

**Primary Funding Source:** National Institutes of Health

### S-21 The micropolitics of implementation: A qualitative study exposing an unexplored path within implementation science

#### Lisa Rogers^1^, Aoife De Brún^1^, Sarah Birken^2^, Carmel Davies^1^, Eilish McAuliffe^1^

##### ^1^University College Dublin, Dublin, Ireland; ^2^Wake Forest School of Medicine, Winston-Salem, NC, USA

###### **Correspondence:** Lisa Rogers (lisa.rogers@ucdconnect.ie)

**Background:** Healthcare systems are complex, inherently political structures. Each healthcare organisation comprises of multiple stakeholders with differing priorities, roles, and expectations about how care should be delivered. To reach agreement among these divergent interest groups while ensuring optimum patient care, staff must navigate the micropolitical context of their healthcare organisation. Micropolitics in this study refers to the use of power, authority, and influence to affect team goals, vision, and decision-making processes. Although these concepts are influential when cultivating change, the micropolitical context of healthcare has received scant attention in implementation science literature. This research addresses this gap by exploring the role of power, authority, and influence when implementing a collective leadership intervention among two multidisciplinary healthcare teams.

**Methods:** The multiple case study design adopted employed a triangulation of qualitative research methods. Thirty-one hours of observations (Case A=16, Case B=15) and twenty-five interviews (Case A=13, Case B=12) were completed. An in-depth thematic analysis of the data using an inductive coding approach enabled a greater understanding of the mechanisms through which the micropolitical context influences implementation success.

**Findings:** The findings emphasised that implementing change in healthcare teams is an inherently political process heavily influenced by prevailing power structures. Two key themes were generated which revealed the dynamic role of power, authority, and influence throughout implementation. The first highlights that gaining support across multiple levels of leadership is critical to implementation success as the influence exercised by these individuals persuades follower engagement. However, the second theme emphasises that the historical dynamics of teams will determine how this influence is exerted and perceived, which can negatively impact participants’ experiences of implementation.

**Implications for D&I Research:** To date, micropolitics has received scant attention in implementation research. Although the extant literature has explored *what* relationships exist within multidisciplinary healthcare teams, understanding the micropolitical context of healthcare helps explain *why* these team dynamics occur. By introducing the concepts of power, authority and influence as essential contextual determinants, researchers can better negotiate the everyday politics of healthcare and develop more appropriate implementation strategies to create contexts receptive to change.

**Primary Funding Source:** Irish Health Research Board

### S-22 Advances in d&I research training: A decade of multimethod data from the IRI

#### John Landsverk^1^, Enola Proctor^2^, Douglas Luke^3^, Bobbi Carothers^4^, Ana Baumann^4^

##### ^1^Oregon Social Learning Center, Eugene, OR, USA; ^2^George Warren Brown School of Social Work, Washington University in St. Louis, St. Louis, MO, USA; ^3^Center for Public Health Systems Science, Washington University in St. Louis, Saint Louis, MO, USA; ^4^Washington University in St. Louis, St. Louis, MO, USA

###### **Correspondence:** John Landsverk (jlandsverk@aol.com)

**Background:** Through its networked “hands on” training for mental health implementation science, the Implementation Research Institute (IRI) directly responds to NIMH’s current strategic plan and its research training report *Investing in the Future*. Over ten years (2010-2020), the IRI has advanced its evaluation approaches through multiple annual surveys across 8 cohorts, social network analyses (SNA), and use of publicly available databases to examine scholarly product outcomes. We report training objectives, outcomes, and implications for advancing D&I research training.

**Methods:**

Annual surveys capture IRI participant collaboration on research grants, publications, peer mentoring, and satisfaction with training and mentoring. Library scientists perform bibliometric searches on publications of IRI fellows and applicants not selected (comparison group). Training aims and program logic model guide data analysis, employing visualization and descriptive and inferential statistics. We perform annual data collection and analysis within each year and cohort, as well as longitudinally across cohorts. Longitudinal mixed-effects modeling assesses change over time and identifies predictors of change. SNA captures how IRI develops mentoring relationships, leading to new scientific collaborations and new implementation science products.

**Findings:**

SNA findings reveal a dense, interconnected IRI network, ongoing contact, growth in scientific collaboration, and stability over time. Collaborations for IRI fellows on grant submissions increased over time, with a strong relationship between amount of mentoring and number of D&I research collaborations. Bibliometrics found that proportion of D&I publications increased by 7% compared to no gain for IRI applicants not selected. NIH Reporter searches showed fellows showed an 11% gain in D&I grants funded, compared to a 5% gain for applicants not accepted. For cohorts 5-8, applicants not accepted and fellows do not differ in year one but differences favoring fellows increased each subsequent year.

**Implications for D&I Research:**

Evaluation methods for D&I training have become more precise, independent of trainee self-report and supportive of longitudinal analyses in comparative designs, using application information to adjust for co-variates. Improvement of evaluation measurement and design provides preliminary confirmation for the role of training, mentoring and development of collaboration leading to D&I research outcomes.

**Primary Funding Source:** National Institutes of Health

### S-23 Integrating implementation science in clinical research to maximize public health impact: A call for the reporting of implementation strategy use in clinical research

#### Brittany Rudd^1^, Molly Davis^2^, Rinad Beidas^2^

##### ^1^University of Illinois at Chicago, Chicago, IL, USA; ^2^University of Pennsylvania Perelman School of Medicine, Philadelphia, PA, USA

###### **Correspondence:** Brittany Rudd (bnrudd@uic.edu)

**Background:**

As implementation researchers who study methods to promote the systematic uptake of evidence-based clinical practices (i.e., clinical interventions) into routine care to improve health, we are the last step in the translational research continuum to ensure public health impact of discovery. However, our programs of research draw upon the findings of our colleagues who work in earlier stages of the translational continuum, particularly clinical researchers who study the efficacy and effectiveness of clinical interventions. In order to conduct methodologically rigorous RCTs, clinical researchers expend significant resources to monitor the clinical intervention’s delivery and to support its implementation, meaning many clinical researchers are using elements of implementation science in their research programs. Differences in terminology used by scientists across the translational research continuum have left these “elements,” which would be referred to as “implementation strategies” in implementation science, unlabeled and under-reported in clinical research. We propose a novel way to accelerate research-to-practice implementation – revising frequently used reporting guidelines in clinical research to elicit the implementation strategies that clinical researchers are already using in their clinical efficacy and effectiveness trials.

**Methods:**

First, we systematically review clinical and implementation research reporting guidelines for randomized controlled trials from the Enhancing the QUAlity and Transparency Of health Research website (https://www.equator-network.org/) and integrate recent work to prospectively and retrospectively track implementation strategy use. Second, informed by our review, we propose revisions to standard clinical research reporting guidelines (e.g., CONSORT). Third, we share our Pragmatic Implementation Strategy Reporting Tool for use with the revised reporting guidelines. Fourth, we pilot the tool by coding a published effectiveness study for mention of implementation strategies using the tool.

**Findings:**

The primary clinical reporting guidelines (e.g., CONSORT, SPIRIT, TIDieR) provided limited prompting for implementation strategy reporting. The Pragmatic Implementation Strategy Reporting Tool was feasible to use when reporting implementation strategy use for an effectiveness study.

**Implications for D&I Research:**

We hope that specifying implementation strategy use earlier in the translational spectrum will accelerate translation from clinical to implementation research in order to expedite improvements in public health.

**Primary Funding Source:** National Institutes of Health

## Clinical Care Settings: Patient-level Interventions

### S-24 Cost of implementing collaborative care for perinatal depression

#### Lisa Saldana^1^, Tess Grover^2^, Mark Campbell^1^, Mindy Vredevoogd^2^, Ian Bennett^2^

##### ^1^Oregon Social Learning Center, Eugene, OR, USA; ^2^University of Washington, Seattle, WA, USA

###### **Correspondence:** Lisa Saldana (lisas@oslc.org)

**Background:**

Limited rigorous evaluation has examined the costs associated with implementing complex innovations for management of chronic illness in primary care. Cost assessments are needed to inform implementation planning. The evidence based Collaborative Care Model (CoCM) was implemented in 10 primary care clinics for treatment of common perinatal mental health disorders as part of a larger national randomized clinical trial.

**Methods:**

The Cost of Implementing New Strategies (COINS) approach was used to examine implementation costs, above and beyond intervention costs, necessary to implement CoCM. Standardized COINS methods were utilized, including defining implementation activities using the Stages of Implementation Completion (SIC) -- a well-validated measure of implementation process and milestones from Engagement (Stage 1) to Competency (Stage 8). The SIC and COINS measures, data collection, and tracking tools were tailored for the CoCM for perinatal depression implementation approach. Observational periods were limited to approximately 2 years post signing of the Memorandum of Agreement, per clinic.

**Findings:**

As measured by the SIC, two clinics discontinued implementations, five implemented CoCM but were not yet competent, and three achieved competent delivery. The three competent clinics showed a range of efficiency (62, 107, 109 weeks each). Across all clinics, significant levels of staff effort were needed to complete Readiness Planning, Hiring and Training, Establishing Fidelity Monitoring, and Ongoing Service Delivery stages (average = 64, 48, 54, 50 hours respectively). Clinical staff and the program champion were most involved in implementation activities. Cost estimates were calculated for each position using wage norms. On average, clinics that delivered CoCM spent $40,690 on implementation, but demonstrated a wide range of expenses $4,409 - $103,156. Of the three competent clinics, all did so relatively efficiently; range of $20,944 - $65,249 (mean = $41,720). Implementation costs do not include intervention costs.

**Implications for D&I Research:**

Implementation costs can be challenging to capture but if left undefined can lead to uninformed decision making. Evidence from the first 10 clinics recruited to implement CoCM suggests that programs that are most successful, are ones that also are efficient in their implementation approach.

**Primary Funding Source:** National Institutes of Health

### S-25 Informing de-implementation of low-value breast and prostate cancer practices

#### Ted Skolarus^1^, Daniela Wittmann^1^, Jane Forman^2^, Jordan Sparks^2^, Tabitha Metreger^3^, Anne Sales^1^, Alan Paniagua-Cruz^4^, Kristian Stensland^1^, Sarah Hawley^1^, Ton Wang^1^, Alison Baskin^1^, Nicole Mott^1^, Lesly Dossett^1^

##### ^1^University of Michigan, Ann Arbor, MI, USA; ^2^Veterans Health Administration, VA Ann Arbor Healthcare System, Ann Arbor, MI, USA; ^3^Veterans Health Administration, VA HSR&D Center for Clinical Management Research, Ann Arbor, MI, USA; ^4^University of Michigan Medical School, Ann Arbor, MI, USA

###### **Correspondence:** Ted Skolarus (tskolar@med.umich.edu)

**Background**: As two of the most common malignancies, addressing overtreatment of breast and prostate cancer is a public health priority. Despite continued use of many low-value practices, there has been some de-implementation of low value cancer care with limited formal efforts. Our objective was to understand provider-level behavioral determinants of de-implementation to inform theory-based strategy development. Guided by the Theoretical Domains Framework (TDF), we compared qualitative data regarding largely de-implemented though persistent low-value breast and prostate cancer practices.

**Methods**: We used qualitative data coded into TDF domains from 38 semi-structured provider interviews (18 breast and 20 prostate). Interviews explored knowledge, attitudes, and beliefs regarding low-value practices according to their specialty, specifically: 1) complete axillary lymph node dissection for low volume breast cancer node involvement, 2) re-excision of negative lumpectomy margins for invasive breast cancer, and 3) androgen deprivation therapy injections (ADT) for localized prostate cancer. We then mapped the most relevant TDF domains according to each practice to explore similarities and differences across cancer types.

**Findings**: We identified similar TDF domains for two low-value practices: negative margin re-excision and ADT injections. Shared domains among these practices included: knowledge, skills, beliefs about consequences, goals, and social influences (Table 1). In contrast, and in light of 2011 and 2013 randomized trial evidence indicating no survival benefit, we found knowledge and social influence most relevant for de-implementation of additional lymph node dissection. We found limited de-implementation relevance for several TDF domains regardless of practice type.

**Implications for D&I Research:** We characterized important behavioral domains across low-value breast and prostate cancer care. We identified similarities among relevant domains for two practices, while de-implementation of another appeared to be driven by recent high-level evidence and social norms. Better understanding generalizable behavioral determinants of low-value cancer practices may support theory-based de-implementation strategy tailoring.

**Primary Funding Source:** National Institutes of Health

**Table 1 (abstract S25).** See text for description

TDF Domain	Low-value practice		
	Breast cancer		Prostate cancer
	Additional node dissection	Negative margin re-excision	ADT injections for localized disease
Knowledge	X	X	X
Skills		X	X
Social/ professional role and identity			
Beliefs about capabilities			X
Optimism			
Beliefs about consequences		X	X
Reinforcement			
Intentions			
Goals		X	X
Memory, attention and decision processes			
Environmental context and resources			X
Social influences	X	X	X
Emotion			
Behavioral regulation			

### S-26 Assessing pre-implementation context in the intensive care unit through group model building

#### Mia Vogel^1,2^, Braveheart Gillani^3^, Peter Hovmand^3^, Jose Pineda Soto^4^, Enola Proctor^5^

##### ^1^Washington University in St. Louis, Saint Louis, MO, USA; ^2^Washington University in St. Louis, St. Louis, MO, USA; ^3^Case Western Reserve University, Cleveland, OH, USA; ^4^Children's Hospital Los Angeles, Los Angeles, CA, USA; ^5^George Warren Brown School of Social Work, Washington University in St. Louis, St. Louis, MO, USA

###### **Correspondence:** Mia Vogel (miavogel@wustl.edu)

**Background:**

Technology-driven approaches for better integration of pediatric severe traumatic brain injury (sTBI) guidelines can improve decision making and outcomes in critical care. Implementing technology-driven approaches in pediatric intensive care units (PICUs) requires adaptation and tailoring of implementation strategies to the context of PICUs. Group model building (GMB)^1^ is a participatory systems science method that has been proposed as an innovative approach to adapting and tailoring implementation strategies.^2^ In this study, GMB was used to map the implementation context of a technology-driven approach for integrating best available evidence for pediatric sTBI across 4 PICUs in the United States.

**Methods:**

Nurses, trainees, and attending physicians participated in 90-minute, profession-specific GMB sessions at each of 4 study sites. Participants were asked to share their PICU workflow through a series of facilitated GMB exercises generating causal loop diagrams (CLDs) to build a shared mental model of the implementation context as a system. Context analysis of the resulting CLDs was then used to compare the implementation context across professions and implementation sites.

**Findings:**

Seven CLDs were generated representing profession-specific mental models of workflow and information delays with regard to pediatric sTBI care. Across professions, nurses' CLDs were generally more complex than attending physicians'. Overall themes included communication, leadership, education, resources, and protocols. The CLDs of the implementation context were then used to tailor implementation strategies of a computerized pathway linking Brain Trauma Foundation guidelines to waveform patient data to aid evidence-based decision making in the sTBI.

**Implications for D&I Research:**

Results could inform best practices for implementing evidence-based guidelines in critical care contexts. Group model building is an effective method for generating support and reaching consensus on a shared mental model of the system; it was useful in assessing workflow and implementation context in the ICU. The ICU is a complex system, and group model building and similar systems science approaches show much promise for advancing implementation science.

References

1. Vennix J. *Group model building.* New York: John Wiley & Sons; 1996.
2. Powell BJ, Beidas RS, Lewis CC, et al. Methods to Improve the Selection and Tailoring of Implementation Strategies. *The journal of behavioral health services & research.* 2017;44(2):177-194.

**Primary Funding Source:** National Institutes of Health

### S-27 Theory-informed development of a toolkit for implementation of the segmental approach to assessement and intervention for trunk control.

#### Danielle Bellows^1^, Catie Christensen^2^, Abigail Kremer^2^, Brooke Bernstein^2^, Lori Grisez^2^, Kristin Welch^2^, Rachel Bican^2^, Jason Hubeny^3^, Jason Bracich^3^, Alexander Backus^3^, James Harkin^3^, Jamie Kane^3^

##### ^1^MCPHS University, Worcester, MA, USA; ^2^Nationwide Children's Hospital, Columbus, OH, USA; ^3^University of Hartford, West Hartford, CT, USA

###### **Correspondence:** Danielle Bellows (danielle.bellows@mcphs.edu)

**Background:** The Segmental Assessment of Trunk Control (SATCo) is a recommended measure of posture control for children with moderate to severe cerebral palsy. Specific scoring, special equipment, and manual skill make SATCo implementation challenging and research is limited on application of SATCo results to intervention. Identifying and addressing barriers to implementation through development of a toolkit of clinical resources may facilitate implementation of segmental assessment and intervention. The purpose of this study was to develop, implement and plan for evaluation of a toolkit to address barriers and leverage enablers for implementation of a segmental approach to trunk control in pediatric rehabilitation.

**Methods:** Nineteen pediatric physical therapists and one rehabilitation aide participated in focus groups across six clinic locations within a single mid-western pediatric specialty hospital to identify perceived barriers and enablers. A semi-structured interview guide was developed based on the Theoretical Domains Framework (TDF). Two researchers independently coded each verbatim transcript using a qualitative directed content analysis approach with TDF as the framework. Conflicts were resolved using a consensus process. Barriers were mapped to change strategies using the TDF/Behavior Change Wheel and conceptualized as an “implementation tool kit” to foster uptake of the segmental approach.

**Findings:** The most relevant TDF domains were therapist skill, confidence, beliefs about consequences, and environmental context/resources. A local implementation team and academic research team collaboratively identified and developed tool-kit products to address specific barriers. Products included: decision algorithms to guide intervention activities for improved trunk control, online education modules to increase therapist knowledge about the segmental approach, caregiver resources for discussing trunk control with community providers, and do-it-yourself instructions for caregivers to adapt existing or build new inexpensive equipment to facilitate segmental support in daily activities at home. The principal investigator provided monthly coaching to the implementation team.

**Implications for D&I Research:** We used the TDF to identify and map barriers to change strategies to foster increased use of the segmental approach in the outpatient department of a pediatric specialty hospital. Future research should investigate the effectiveness of these tool-kit resources and outcomes at patient, therapist, and healthcare system levels using an evaluation framework such as RE-AIM.

**Primary Funding Source:** University of Hartford Institute for Translational Research

### S-28 Achieving sustained adherence to lung protective ventilation: Lessons from a retrospective review of a multi-year implementation program

#### Lauren Allen^1^, Andrew Knighton^2^, Doug Wolfe^2^, Jason Jacobs^2^, Lori Carpenter^1^, Carrie Winberg^1^, Ithan Peltan^2^, Colin Grissom^2^, Raj Srivastava^2^

##### ^1^Intermountain Healthcare, Murray, UT, USA; ^2^Intermountain Healthcare, Salt Lake City, UT, USA

###### **Correspondence:** Lauren Allen (lauren.allen@imail.org)

**Background:**

Lung protective ventilation (LPV) is associated with reduced mortality and morbidity for individuals with acute respiratory distress syndrome, yet variation in LPV use persists globally. Intermountain Healthcare initiated a program to increase LPV use in 2017. In 2019, Intermountain received a two-year planning grant from the NHLBI (award number U01HL143505) for a three-site pilot study using targeted implementation strategies to achieve high adherence.

**Methods:**

Retrospective program review from inception was done following pilot completion to analyze the program journey. We measured variation in LPV adherence from January 2017-June 2020 using a time-weighted average of low tidal volume ventilation (≤6.5ml/kg) while on volume control and appropriate continuous positive airway pressure/pressure support settings (FIO2 ≤50%, PEEP ≤10, PS≤ 15) while weaning from ventilation. Comparisons were evaluated using a two-sample test of proportion (p<.05). We categorized implementation strategies, linked these to LPV adherence data, and developed implementation resource intensity estimates.

**Findings:**

LPV adherence increased significantly from 47% to 92% (p.<001). Four phases in the implementation program development were identified, associated with a change in implementation strategy followed by a sustained increase and plateau (Table 1). Early phase strategies (1&2) involved largely one-way communication to field teams at minimal cost. Later phase strategies (3&4) associated with the pilot study were more labor-intensive, involved more collaborative methods, and included barrier and facilitator identification and strategies tailored to address slow adopters.

**Implications for D&I Research:**

Low cost strategies effective in the early phases of a multi-year deployment were less effective in later phases; more resource-intensive strategies were required to address slow adopting clinicians. Given implementation resource constraints, research is needed to understand optimal sequencing of implementation strategies to achieve high adherence at the lowest possible cost.

**Primary Funding Source:** National Institutes of Health

**Table 1 (abstract S28).** Retrospective program review results

				Lung Protective Ventilation Adherence			
Phase	Time Period	Key Implementation Strategies	Implementation Resource Intensity	Start	End	Change	p-value
1	Q1 2017-Q4 2017	Center-led support, clinician education	Low	0.47	0.57	0.10	.01
2	Q1 2018-Q4 2018	Renewed leadership emphasis	Low	0.57	0.71	0.14	<.001
3	Q1 2019-Q3 2019	Listening tour, field conversations	Moderate	0.71	0.84	0.13	<.001
4	Q4 2019-Q2 2020	Local quality improvement teams, audit/feedback	High	0.84	0.92	0.08	<.001

### S-29 Advance care planning via group visits: Partnering to discern implementation strategies from stakeholder activities

#### Jacob Painter^1,2^, Monica Matthieu^2,3^

##### ^1^University of Arkansas Medical Sciences, Little Rock, AR, USA; ^2^Veterans Health Administration, Central Arkansas Veterans Healthcare System, Little Rock, AR, USA^3^Saint Louis University, St. Louis, MO, USA

###### **Correspondence:** Jacob Painter (Jacob.Painter@va.gov)

**Background:**

The process by which implementation strategy selection occurs is generally mired in a black box of stakeholder “wants and needs”. In this individual oral presentation, we will describe a methodology employed by our team to select and operationalize a broad set of implementation strategies by leveraging the Expert Recommendations for Change (ERIC) framework in a QUERI funded national program evaluation.

**Methods:**

This work is part of a mixed methods program evaluation aimed to investigate the implementation of Advance Care Planning (ACP) via Group Visits (ACP-GV) across the Department of Veterans Affairs (VA) healthcare system (N=152). To examine the cost of the program, a budget impact analysis in underway; to accurately estimate the cost of time spent implementing the program it was necessary to identify implementation activities conducted by ACP-GV stakeholders. The ERIC taxonomy of 73 implementation strategies was used to retrospectively identify all strategies in use in the ACP-GV National Program. Each strategy was evaluated using ERIC definitions by a former member of the ERIC team to identify implementation activities. A group of ACP-GV stakeholders provided insight, clarification and feedback on how implementation activities were performed and helped to discern how these activities fit into the taxonomy.

**Findings:**

56 implementation activities were assigned to 20 strategies representing 6 ERIC clusters and 5 stakeholder groups. Based on the identified strategies, the team developed survey items using the list of implementation activities cross-walked with implementation strategies. These items were then translated into a language that was commonly understood by VA providers. The resulting set of survey items will be used to query stakeholder groups to identify time spent in each of these implementation activities so costs can be assigned for use in the budget impact analysis.

**Implications for D&I Research:**

One challenge to estimating implementation costs is conducting an exhaustive review of program implementation to identify all related stakeholder activities. However, review and thoughtful assignment of these activities within a taxonomy is necessary to appropriately estimate time devoted to implementation. This presentation will describe an innovative approach to working with stakeholders to identify implementation activities and strategies using the ERIC framework.

**Primary Funding Source:** QUERI and ORH

### S-30 Improving evidence-based practice implementation in nursing homes: Effect of using a frontline staff “watch list” huddle implementation strategy

#### Christine Hartmann^1,2^, Ryann Engle^3^, Valerie Clark^1^, A. Lynn Snow^4,5^

##### ^1^Substance Abuse and Mental Health Services Administration, Bedford, MA, USA; ^2^University of Massachusetts, Lowell, MA, USA; ^3^Veterans Health Administration, VA Boston Healthcare System, Boston, MA, USA; ^4^Veterans Health Administration , Tuscaloosa VA Medical Center, Tuscaloosa, AL, USA; ^5^University of Alabama, Tuscaloosa, AL, USA

###### **Correspondence:** Christine Hartmann (christine.hartmann@va.gov)

**Background:** Nursing home residents are highly vulnerable. Yet nursing homes, including in the Department of Veterans Affairs (VA), provide variable care quality and traditionally have few structures and processes to facilitate high-functioning team communication, though literature indicates this improves outcomes. A “watch list” huddle is designed to address these issues. It is a brief, daily, stand-up meeting where frontline staff collaboratively identify nursing home residents at highest risk, hear from every member of the team, and develop and test evidence-based pilot projects.

**Methods:** Eight of the lowest-performing VA nursing homes were selected based on their quality measures to participate in a 9-month quality improvement collaborative, modeled on the Institute for Healthcare Improvement breakthrough series. The frontline watch-list huddle was the foundational implementation strategy; quality issues addressed included resident sleep, pain, and mobility. The collaborative engaged 4-member teams from each site in 3 virtual and 2 in-person learning sessions, using both virtual and in-person facilitation. Between learning sessions, participants implemented evidence-based practices using the frontline staff watch list huddle strategy. We gauged performance pre and post collaborative using administrative data and post-collaborative qualitative interviews.

**Findings:**

Seven sites made demonstrable improvements in administratively collected quality star ratings (1-5, with 1 being worst) and quality points (Table 1—site H had known internal issues that prevented its full participation in the collaborative). Interview participants consistently attributed part of their improvement in ratings to participation in the collaborative.

**Implications for D&I Research:** The watch list huddle represents a powerful implementation strategy for improving care. It has potential to be used in settings other than nursing homes.

**Primary Funding Source:** Department of Veterans Affairs

**Table 1(abstract S30).** VA nursing home quality points and star ratings pre and post collaborative

VA Nursing Home	Quality Star Rating, FY18, Quarter 3 (points)	Quality Star Rating, FY19, Quarter 3 (points)
A	1 star (450)	**5** stars (830: **+380**)
B	1 star (490)	**4** stars (735: **+245**)
C	2 stars (555)	**4** stars (735: **+180**)
D	1 star (470)	**4** stars (690: **+220**)
E	2 star (550)	**3** stars (635: **+85**)
F	1 star (465)	**2** stars (600: **+135**)
G	1 star (485)	**2** stars (570: **+85**)
H	2 stars (585)	2 stars (565: **-20)**

### S-31 Improving the delivery of team-based care after primary breast cancer treatment through a multi-level intervention: A pilot randomized controlled trial

#### Lauren Wallner^1^, Paul Abrahamse^1^, Archana Radhakrishnan^1^, Lawrence An^1^, Jennifer Griggs^1,2^, Anne Schott^1^, John Ayanian^1^, Anne Sales^1^, Steven Katz^1^, Sarah Hawley^1^

##### ^1^University of Michigan, Ann Arbor, MI, USA; ^2^Michigan Oncology Quality Consortium, Ann Arbor, MI, USA

###### **Correspondence:** Lauren Wallner (lwallner@med.umich.edu)

**Background**

The delivery of team-based care after primary cancer treatment remains challenging, in part due to a lack of effective interventions. We developed a multi-level intervention for breast cancer patients and their primary care and medical oncology providers to improve the delivery of team-based survivorship care called ConnectedCancerCare (CCC). CCC includes a patient-facing, personalized mobile website, and tailored feedback letters to primary care and medical oncology providers.

**Methods:** We conducted a pilot randomized controlled trial in a breast oncology clinic to establish the feasibility and acceptability of CCC. Women within one year of completing primary treatment for stages 0-II breast cancer were randomized to CCC (intervention) or a static online survivorship care plan (control). Participants completed online surveys at baseline and 3 months, ascertaining their knowledge about PCP roles in their survivorship care, their communication with their PCP about team-based care, and whether they scheduled a follow-up visit with their PCP. Multiple measures of acceptability were collected among women in the intervention arm (n = 28). Qualitative interviews were conducted at the completion of the study with 5 PCPs, 6 oncology providers, and 10 intervention patients to identify barriers and facilitators to implementing CCC.

**Findings:** Among 160 eligible women invited to participate, 66 completed the baseline survey and were randomized (41%), and 54 completed the 3-month follow-up survey (83%). The majority of women in the intervention arm found the content of CCC to be highly acceptable. A greater proportion of women randomized to CCC (vs. control) reported scheduling a PCP follow-up visit (64% vs. 42%) and communicating with their PCP about provider roles (67% vs. 18%). Women in the CCC arm also reported higher mean knowledge scores regarding team-based cancer care (3.7 vs. 3.4). Providers noted challenges to implementing CCC, including integration into electronic medical records, and supporting sustained engagement with CCC over time.

**Implications for D&I Research:** Our findings suggest deploying CCC in medical oncology practices is feasible, and the intervention content is acceptable among breast cancer patients. CCC shows promise for improving understanding and communication about provider roles in survivorship care, and facilitating patients to follow up with their PCP early in the survivorship period.

**Primary Funding Source:** National Institutes of Health

### S-32 Examples and challenges of engaging consumers in implementation science activities: An environmental scan

#### Andrea Melgar Castillo

##### University of Arkansas for Medical Sciences, Little Rock, AR, USA

###### **Correspondence:** Andrea Melgar Castillo (amelgarcastillo@uams.edu)

**Background:** Existing research on engaging consumers in implementation processes is limited, yet shows improvements in quality of care. However, the variety of terms used in literature poses challenges to outlining variety of examples and clear guidelines for engaging consumers in implementation. We systematically documented how implementers are engaging consumers in implementation processes, and coded recurring challenges in this cutting-edge work.

**Methods:** For our environmental scan of consumer engagement in implementation processes we gathered information from four sources: 1. A narrow, systematic literature search on consumer engagement in implementation activities. 2. A systematic search of the Veterans Health Administration Health Services Research and Development webinars. 3. Observation of workgroups that discussed possible consumer engagement in implementation processes. 4. Electronic surveys and phone interviews with implementation experts. Two researchers independently coded the collected data into templates, describing their innovation, recipient, context, and health equity domains according to the Health Equity Implementation Framework and also categorizing them by intensity of consumer engagement. We categorized each example into an intensity level of consumer engagement, according to Goodman and Thompson (2017), ranging from education to community based participatory research. Across all data sources, we extracted recurrent challenges for engaging consumers in implementation.

**Findings:**

We identified 26 examples of consumer engagement in implementation of healthcare services in North America. Majority of examples were a medium level of engagement, “coordination” (50%), followed by more intensive engagement, “cooperation” (19%), collaboration” (15% each), “community based participatory research” (4%), patient-centered (4%), and least intensive engagement, “outreach” (4%) and “education” (4%). We identified 9 recurrent challenges when engaging consumers in implementation processes; the top three challenges were: 1. recruiting consumers representative of the population served, 2. lack of processes for equitable communication among stakeholders, and 3. retaining consumers in implementation efforts.

**Implications for D&I Research:** In North America, we found consumers have been engaged in several implementation projects. This study provides a foundation for more robust guidance on consumer engagement in implementation necessary to push implementation science forward to reduce healthcare inequities.

**Primary Funding Source:** Department of Veterans Affairs

### S-33 Creating systems to support consumer engagement in research: Implementation and initial impact of the veteran consulting network

#### Vanessa Merker

##### Center for Healthcare Organizations and Implementation Research (CHOIR), Bedford, MA, USA

###### **Correspondence:** Vanessa Merker (vanessa.merker@va.gov)

**Background:** Engaging consumers in implementation research has potential to enhance the quality and relevance of research studies, and ultimately, the implementation and sustainment of evidence-based practices in clinical care. Researchers often require organizational support to effectively engage consumers in the research process. In fall 2017, the Center for Healthcare Organization and Implementation Research at the Veterans Health Administration began the Veteran Consulting Network to match individual Veterans with research teams for ongoing consultation on the planning, conduct, and dissemination of grants and subsequent studies.

**Methods:** The center used 7 major implementation strategies to promote uptake of the Veteran Consulting Network among researchers, including: coalition building within the organization to design and implement engagement efforts; cultivating champions who supported implementation efforts; accessing new funding to pay Veteran consultants and support the salary of program coordinators who provide technical assistance; conducting trainings and developing educational materials to prepare veterans and staff for engagement; and obtaining ongoing feedback from program users via survey and interviews.

**Findings:** During the first two years of the Veteran Consultant Network, 16/45 (36%) of submitted grants from our center included Veteran consultant input. In a January 2019 survey, >90% researchers (n=62) agreed that creating a center-wide strategy to include Veteran consultants was worthwhile. Trainings and educational resources were rated highly; feedback from researchers indicated additional interest in learning collaborative approaches to increasing capacity for engagement led by champions and early adopters. Interviews with Veteran consultants and researchers (n=23) indicate a range of positive impacts from Veteran engagement, including improved cultural competency and accessibility of intervention components and study instruments. However, interviews also revealed limitations in some researchers’ knowledge of how and when to utilize veteran consultants and some difficulties sustaining engagement due to inadequate follow-up with veteran consultants.

**Implications for D&I Research:** This work highlights the use and impact of specific implementation strategies to facilitate consumer engagement across multiple and diverse research teams in one research center. Continuing efforts to ensure meaningful consumer engagement may be best served by learning collaborative approaches, so that champions and early adopters can share examples of the utility of and best practices for consumer engagement.

**Primary Funding Source:** Department of Veterans Affairs

### S-34 A stakeholder-driven approach to the development of a workforce development initiative to improve care for obsessive-compulsive disorder

#### Sapana Patel

##### Columbia University Department of Psychiatry, New York, NY, USA

###### **Correspondence:** Sapana Patel (Sapana.Patel@nyspi.columbia.edu)

**Background:** Care recipients and family members bring unique perspectives to improve quality of behavioral health care that are often overlooked by researchers, clinicians and administrators. We describe how recipients of behavioral healthcare participated in the development of e-Learning modules and a multifaceted implementation strategy to improve evidence-based care for obsessive-compulsive disorder in a large, public behavioral health system in New York. This workforce development initiative (Improving Providers’ Assessment Care Delivery and Treatment of Obsessive-Compulsive Disorder [IMPACT-OCD]), engaged a diverse stakeholder advisory board comprised of individuals with lived experience, peer community, local advocacy organizations, and local and state leadership from the Office of Consumer Affairs.

**Methods:** Using principles of an instructional design model (Analysis, Design, Development, Implementation, and Evaluation [ADDIE]), board members participated in co-creation and dissemination of e-Learning modules and tools for behavioral health providers. Using the Consolidated Framework for Implementation Research, we solicited board members’ feedback on barriers and facilitators to implementation. These data were used to construct a multifaceted implementation strategy. We iteratively collected qualitative data (e.g., advisory board meetings, ADDIE cycle reviews, interviews) from advisory board members.

**Findings:** Advisory board members identified public misunderstanding about OCD as a barrier which led to a statewide webinar activity on the *Seven Common Misconceptions of OCD* to increase awareness and knowledge about OCD. Subsequently, individuals with lived experience and their anonymized accounts of treatment with an evidence based practice were integrated into the online training courses as a push/pull strategy to motivate recipient help seeking and referrals to specialty care providers. Educational materials to empower recipients was identified as a facilitator and led to collaboration with an advocacy organization and development of a toolkit of resources for children and adults with OCD.

**Implications for D&I Research:** To avoid tokenism and bias, care recipients were offered a continuum of options to participate in the IMPACT-OCD workforce development initiative. Including care recipients helped to identify knowledge gaps, ensure online training materials for providers included the care recipient voice, increase pull or demand for the innovation, and co-create materials to empower our most important end-users.

**Primary Funding Source:** New York State Psychiatric Institute

## Clinical Care Settings: System-level Interventions

### S-35 Coach-based and web-based practice facilitation for HPV vaccination: Implementation and effectiveness outcomes

#### Pamela Hull^1^, Debra Friedman^2^, Jaimie Shing^2^, Maureen Sanderson^3^, Liping Du^2^, Tatsuki Koyama^2^, Shelagh Mulvaney^4^, Kelly Moore^2^, Caree McAfee^1^, Lora Harnack^5^, Christine Stroebel^5^, Janet Cates^5^, Stephen Deppen^2^

##### ^1^University of Kentucky Markey Cancer Center, Lexington, KY, USA; ^2^Vanderbilt University Medical Center, Nashville, TN, USA; ^3^Meharry Medical College, Nashville, TN, USA^4^Vanderbilt University, Nashville, TN, USA; ^5^Cumberland Pediatric Foundation, Brentwood, TN, USA

###### **Correspondence:** Pamela Hull (pam.hull@uky.edu)

**Background:** Practice facilitation (PF) is an effective implementation strategy for improving adherence to clinical guidelines but has not been examined for HPV vaccination. Health systems may consider providing in-person versus remote PF. We compared two formats for delivering PF to engage pediatric practices in quality improvement focused on increasing HPV vaccination.

**Methods:** Pediatric practices (N=21) in Tennessee were randomized to Coach-based or Web-based PF. Each practice selected four change options to implement over 12 months. Using a Type 2 hybrid design, implementation outcomes included two measures of guideline adherence: receipt of HPV vaccine dose during well visits (patients ages 11-17) and bundling HPV with other recommended vaccines for 11-12 year-olds in the same visit. Clinical effectiveness outcomes included two practice-level HPV vaccination coverage measures: 1+ doses and all doses (patients ages 11-17). Interrupted time series analyses of logarithmic rates compared the practices’ 12-month baseline to the 12-month implementation period. H1: Both arms will improve from the baseline period to implementation period. For implementation outcomes, we expected immediate improvement (positive level change) followed by stability or gradual improvement (no or positive slope change). For Effectiveness Outcomes, we expected gradual improvement only (positive slope change). H2: Coach arm will improve more than Web arm on all measures.

**Findings:** For the implementation outcomes, both arms showed immediate 12-18% relative improvements for doses received in well visits (Relative increase: Coach 1.12 fold [95%CI 1.04-1.21], Web 1.18 fold [1.10-1.27]) and immediate 16-19% relative improvements for bundling vaccines (Coach 1.19 fold [1.06-1.35], Web 1.16 fold [1.03-1.31]), all with stable slopes. These changes did not differ by study arm. For the effectiveness outcomes, only the Coach arm showed gradual improvements for 1+ dose coverage (1.006 fold [1.004-1.008]) and all dose coverage (1.006 fold [1.003-1.008]), which were significantly greater than Web. These positive trend changes for the Coach arm translated to 1+ dose and all dose coverage being 7.4% relatively higher by the end of the 12-month implementation period than they would have been without PF.

**Implications for D&I Research:** Both formats equally improved implementation outcomes, while Coach-based PF yielded greater clinical effectiveness improvements. Further research should examine long-term effectiveness and cost differences across formats.

**Primary Funding Source:** National Institutes of Health

### S-36 Implementation evaluation of a complex intervention to improve timeliness of care for veterans with transient ischemic attack

#### Teresa Damush^1,2^, Edward Miech^3^, Nicholas Rattray^4^, Barbara Homoya^5^, Lauren Penney^6^, Ariel Cheatham^4^, Sean Baird^7^, Jennifer Myers^5^, Curt Austin^4^, Laura Myers^4^, Anthony Perkins^2^, Brenna Giacherio^4^, Meetesh Kumar^4^, Lauren Murphy^4^, Dawn Bravata^8^

##### ^1^Veterans Health Administration , VA Precision Monitoring Queri Center, Indianapolis, IN, USA; ^2^Indiana University School of Medicine, Indianapolis, IN, USA; ^3^VA PRIS-M QUERI, Indianapolis, IN, USA; ^4^Veterans Health Administration, Indianapolis, IN, USA; ^5^Veterans Health Administration , Roudebush VA Medical Center, Indianapolis, IN, USA; ^6^Veterans Health Administration, South Texas Veterans Health Care System, San Antonio, TX, USA; ^7^Veterans Health Administration , VA HSR&D Center for Health Information and Communication (CHIC) and VHA HSR&D PRIS-M QUERI; Richard L. Roudebush VA Medical Center, Indianapolis, IN, USA; ^8^Veterans Health Administration , PRIS-M QUERI/Indiana University School of Medicine, Indianapolis, IN, USA

###### **Correspondence:** Teresa Damush (tdamush@iupui.edu)

**Background:** The Protocol-guided Rapid Evaluation of Veterans Experiencing New Transient Neurologic Symptoms (PREVENT) program was designed to address systemic barriers to providing timely guideline-concordant care for patients with transient ischemic attack (TIA). We evaluated an implementation strategy bundle used to promote local adaptation and adoption of a multi-component, complex quality improvement (QI) intervention to improve the quality of TIA care.

**Methods:** PREVENT, a stepped-wedge implementation trial with six geographically diverse sites, employed a bundle of key implementation strategies: Team Activation; External Facilitation; and a Community of Practice. This strategy bundle had direct ties to four constructs from the Consolidated Framework for Implementation Research (CFIR): Champions, Reflecting & Evaluating, Planning, and Goals & Feedback. Participants were six facility QI teams comprised of multi-disciplinary, clinical staff across clinical settings: ER staff, hospitalists, neurologists, nursing, pharmacy and systems redesign. Using a mixed-methods approach guided by the CFIR and data matrix analyses, we evaluated the degree to which implementation success and clinical improvement were associated with implementation strategies. The primary outcomes were the number of completed implementation activities, the level of team organization and >15 points improvement in the Without Fail Rate (WFR) Over 1-Year.

**Findings:** Facility QI teams actively engaged in the implementation strategies with high utilization. Facilities with the greatest implementation success were those with central champions whose teams engaged in planning and goal setting, and regularly reflected upon their quality data and evaluated their progress against their QI plan. The strong presence of effective champions acted as a pre-condition for the strong presence of Reflecting & Evaluating, Goals & Feedback, and Planning (rather than the other way around), helping to explain how champions at the +2 level influenced ongoing implementation.

**Implications for D&I Research:** The CFIR-guided bundle of implementation strategies facilitated the local implementation of the PREVENT QI program and was associated with clinical improvement in the national VA healthcare system.

**Primary Funding Source:** Department of Veterans Affairs

### S-37 Project MIMIC (Maximizing Implementation of Motivational Incentives in Clinics): Process data from a type 3 hybrid trial comparing strategies for implementing contingency management

#### Sara Becker^1^, Bryan Garner^2^, Bryan Hartzler^3^, Carla Rash^4^, Cara Murphy^5^, Tim Janssen^6^

##### ^1^Brown School of Public Health, Providence, RI, USA; ^2^RTI International, Research Triangle Park, NC, USA; ^3^Seattle, WA, USA; ^4^Farmington, CT, USA; ^5^Brown University School of Public Health, Providence, RI, USA; ^6^Providence, RI, USA

###### **Correspondence:** Sara Becker (sara_becker@brown.edu)

**Background:** Contingency management (CM) is one of the most effective behavioral interventions for persons with opioid use disorder, but one of the least implemented in opioid treatment programs (OTPs). Project MIMIC (Maximizing Implementation of Motivational Incentives in Clinics) is a type 3 hybrid effectiveness-implementation trial comparing two different strategies for helping OTPs and their staff integrate CM. This submission reports on process data from the project’s first cohort of OTPs.

**Methods:** Eight OTPs and their staff were cluster randomized to receive either the Addiction Technology Transfer Center (ATTC) strategy (workshop + feedback + coaching) or the Enhanced ATTC (EATTC) strategy, which layered in two additional strategies: Pay-For-Performance and Implementation Sustainment Facilitation. Activities proceeded sequentially using the exploration, preparation, implementation, and sustainment (EPIS) framework. OTPs completed 5 months of preparation and 7 months of implementation, with OTPs currently completing the project’s 6-month sustainment phase.

**Findings:** During the preparation phase, 52 staff (28 ATTC, 24 EATTC) completed a baseline survey. Of those enrolled, 41 (75% ATTC, 83% EATTC) staff participated in a didactic CM workshop and 31 (54% ATTC, 67% EATTC) submitted a role play for performance feedback. During the implementation phase, each OTP had a target of enrolling 25 CM patients. OTPs receiving the ATTC strategy recruited 88% of the target, while OTPs receiving EATTC recruited 100%. Of the 58 CM session recordings submitted for feedback, 29 met the skills benchmark: 8 ATTC and 21 EATTC.

**Implications for D&I Research:** Project MIMIC advances implementation research by experimentally testing strategies for improving the implementation and sustainment of evidence-based innovations in practice settings. Our project’s preliminary findings suggest that the experimental EATTC strategy resulted in higher CM recruitment, training engagement, and session submissions than the ATTC control strategy. Next steps will examine the impact of the EATTC strategy on the number of CM sessions implemented with patients, reductions in patient’s days of opioid use over time, and OTPs sustainment of CM following removal of active support.

**Primary Funding Source:** National Institutes of Health

### S-38 Pragmatic hybrid effectiveness/implementation trial studying a CDC-based antibiotic stewardship program within a large, multi-center network of urgent care clinics

#### Kim Brunisholz^1,2^, Harris Carmichael^3^, Eddie Stenehjem^3^, Nora Fino^4^, Anthony Wallin^1^, Park Willis^1^, Naresh Kumar^3^, Whitney Buckel^3^, Raj Srivastava^1^, Matthew Samore^5^, Adam Hersh^2^

##### ^1^Intermountain Healthcare, Salt Lake City, UT, USA; ^2^University of Utah, Salt Lake City, UT, USA; ^3^Intermountain Healthcare, Murray, UT, USA; ^4^University of Utah Alumni Board, Salt Lake City, UT, USA; ^5^Veterans Health Administration, VA Salt Lake City Healthcare System, Salt Lake City, UT, USA

###### **Correspondence:** Kim Brunisholz (kim.brunisholz@imail.org)

**Background:** Urgent Care (UC) is a rapidly growing setting to deliver healthcare in the U.S. The CDC developed the Core Elements for Outpatient Antibiotic Stewardship--or, evidence-based strategies designed to reduce antibiotic overuse which remain poorly studied in UC settings. The objective was to examine effectiveness and implementation outcomes associated with a multi-faceted UC Stewardship program within a network of 39 UC clinics in the Intermountain West.

**Methods:** We conducted a non-randomized, type II hybrid effectiveness/implementation study of the UC stewardship program based on the CDC Core Elements. The intervention ran between July 2019-June 2020. Interim results after 7 months are presented to account for practice changes due to COVID19. The intervention was multi-faceted consisting of: education for patients/providers; media campaigns; EHR tools; prescribing dashboard for clinicians showing their up-to-date practice. UC clinician compensation changed from RVU- to shift-based pay with a quality pay-for-performance component focused on antibiotic prescribing. Effectiveness was measured by percentage of all respiratory visits where an antibiotic was prescribed. Implementation penetration among UC clinicians to change their prescribing practices was assessed (as defined by UC system leadership as the percentage who decreased respiratory prescribing by >10% or fell below 50%, the system pre-intervention respiratory prescribing rate average). A binomial mixed effects hierarchical model was used, calculating the odds of antibiotic prescribing associated with the intervention period accounting for pre-intervention trends and clustering within providers and clinics.

**Findings:** The overall number of UC encounters during the study period was 1,559,403 (1,163,849 pre-intervention; 395,554 post-intervention) and 41.5% were respiratory conditions. The percentage of patients with respiratory conditions that received an antibiotic prescription declined from 49.9% pre-intervention to 35.3% during the intervention (OR 0.73, 95%CI: 0.71-0.76), reaching a low of 30% during February 2020. Of 103 clinicians above goal threshold, 83.5% decreased respiratory prescribing rate by >10% or fell below the 50% pre-intervention system average.

**Implications for D&I Research:**This study addresses a critical knowledge gap in how to measure implementation to ensure effectiveness of a large, multi-center UC Stewardship program. After 7 months of the intervention, the UC Stewardship program was associated with reduced respiratory prescribing across a large network of UC clinicians who changed their prescribing behavior.

**Primary Funding Source:** Centers for Disease Control and Prevention

### S-39 Implementation strategies identified in state Medicaid programs’ natural experiments to improve substance use disorder treatment

#### Erika Crable^1^, Allyn Benintendi^2^, David Jones^1^, Alexander Walley^2^, Jacqueline Hicks^1^, Mari-Lynn Drainoni^2^

##### ^1^Boston University School of Public Health, Boston, MA, USA; ^2^Boston University School of Medicine, Boston, MA, USA

###### **Correspondence:** Erika Crable (ecrable@bu.edu)

**Background:** High rates of alcohol misuse, the opioid epidemic, and Medicaid expansion-related health costs have prompted Medicaid agencies to implement comprehensive, substance use disorder (SUD) care continuums. Some Medicaid agencies are seeking to adopt the American Society of Addiction Medicine (ASAM) Criteria as an evidence-based model to define new SUD care continuums. There is limited research describing how policymakers and providers collaborate to implement large-scale systems transformations in low-resource, public healthcare settings. This study examined 3 Medicaid agencies’ experiences implementing ASAM-driven SUD care continuums. We developed a taxonomy of implementation strategies used in Medicaid providers’ statewide adoption of EBPs.

**Methods:** We conducted 44 semi-structured interviews with Medicaid staff and health system partners [e.g. state health agencies, managed care organizations (MCOs), providers] in CA, VA, WV. Interviews were triangulated with document review of state readiness and implementation plans. The Exploration, Preparation, Implementation, Sustainment (EPIS) framework guided our implementation theme analysis. The Expert Recommendations for Implementing Change and Proctor (2013) specification criteria guided identification of implementation strategies. IRB approval was obtained.

**Findings:** We identified four common implementation experiences. (1) Medicaid agencies oscillated between adapting ASAM Criteria to the service environment, and altering setting dynamics to accommodate ASAM specifications. (2) Fidelity to ASAM was thwarted by providers’ lack of familiarity with the care model, and MCO utilization management and prior authorization requirements that contradicted ASAM specifications. (3) Providers assumed financial risk while new service documentation requirements and reimbursement processes were established. (4) Despite statewide implementation successes, gaps remain in Medicaid SUD care continuums due to inadequate provider networks. Medicaid agencies and partners used 29 implementation strategies including strategies used multiple times across phases (Exploration=6; Preparation=10, Implementation=22; Sustainment=6). Strategies to develop stakeholder interrelationships, financial strategies, evaluative and iterative strategies were most frequently used.

**Implications for D&I Research:** This study enhances our understanding of large-scale systems transformation in low-resource, public healthcare settings. Themes highlight fit issues between national EBPs and local service environments. Prior research specifying implementation strategies examined controlled research conditions and emphasized the need to study strategies as they are used in practice. This study reveals a taxonomy of strategies employed by policymakers and providers across states.

**Primary Funding Source:** The Lifespan/Brown Criminal Justice Research Program on Substance Use and HIV

### S-40 The implementation and sustainability of community health worker programs: Lessons learned from a process evaluation across ten sites

#### Melissa Davoust^1^, Sara S. Bachman^2^, Allyson Baughman^3^, Mari-Lynn Drainoni^1,4,5^, Serena Rajabiun^6^, Hill L. Wolfe^1^, Linda S. Sprague Martinez^3^

##### ^1^Boston University School of Public Health, Boston, MA, USA; ^2^University of Pennsylvania School of Social Policy & Practice, Philadelphia, PA, USA; ^3^Boston University School of Social Work, Boston, MA, USA; ^4^Boston University School of Medicine, Boston, MA, USA; ^5^Center for Healthcare Organization and Implementation Research, Edith Nourse Rogers Memorial Veterans Hospital, Bedford, MA, USA; ^6^University of Massachusetts, Lowell, Lowell, MA, USA

###### **Correspondence:** Melissa Davoust (mdavoust@bu.edu)

**Background:** Community health workers (CHW) have been increasingly recognized as a strategy to support people living with HIV (PLWH) in their care and reduce inequities in clinical outcomes, especially among racial and ethnic minorities. In 2016, the Health Resources and Services Administration HIV/AIDS Bureau funded a team from Boston University as a technical assistance (TA) and evaluation center to help 10 Ryan White HIV/AIDS Program (RWHAP) recipient sites across 8 states integrate CHWs into their multidisciplinary care teams. This presentation will focus on the strategies organizations used to implement their CHW programs, along with other factors associated with program adoption, implementation, and maintenance.

**Methods:** A process evaluation guided by RE-AIM was conducted to understand how organizations integrated CHWs into their care teams. Site team members participated in focus groups to walk-the-process at baseline and follow-up, and the transcripts were coded using a directed content analysis approach guided by the implementation strategies outlined in the Expert Recommendations for Implementing Change (ERIC) project^1^.

**Findings:** Implementation strategies related to organizational change were most frequently cited by site team members, followed by those focused on operations along with training and TA. Having an organizational champion at the supervisor level or above and a culture committed to addressing health equity facilitated program adoption. Implementation was facilitated by having accessible centralized TA; learning communities to connect CHWs and supervisors; clear role definition between CHWs and other care team members; informal and formal engagement of care team members via co-location or team huddles; and established policies and procedures on home visits, transporting clients, and accompanying clients to appointments. Finally, ensuring the CHW program connected with populations that sites had previously been unable to engage in care and using creative funding strategies facilitated program maintenance.

**Implications for D&I Research:** Findings from this work have policy and practice implications for the implementation and sustainability of CHW programs designed to support PLWH. In addition, identifying the strategies organizations used to integrate CHWs into their care teams may help others interested in implementing these types of programs to address health inequities in a number of chronic conditions.

Reference:

1. Powell BJ, et al. *Implement Sci*. 2015;10(1):21. doi:10.1186/s13012-015-0209-1

**Primary Funding Source:** Health Resources and Services Administration

### S-41 Using the consolidated framework for implementation research (CFIR) to evaluate fidelity variation amidst a primary care team implementation

#### Lori Merkel^1^, Linda Fleisher^2^, Marianna LaNoue^1^, Steven Spencer^3^, Mitchell Kaminiski^1^

##### ^1^Thomas Jefferson University, Philadelphia, PA, USA; ^2^Fox Chase Cancer Center, Philadelphia, PA, USA; ^3^Jefferson Health, Abington, PA, USA

###### **Correspondence:** Lori Merkel (lori.merkel@jefferson.edu)

**Background:** Primary care teams (PCT) are an evidence-based intervention that can increase cohesion across the continuum of care and reduce unnecessary, redundant, and expensive unplanned acute service utilization. Initiated using funds from a 5-year CMS grant, PCTs were established as a centralized resource comprised of a nurse care coordinator, a clinical ambulatory pharmacist, and a behavioral health consultant. This PCT-based initiative was deployed across 33 practices in a community-based primary care network.

**Methods:** This mixed methods study guided the development of two different dimensions of fidelity to assess EBP uptake. First, the frequency in which practices used this new resource for their high-risk patients was calculated as ‘primary fidelity’. A ‘secondary fidelity’ examined whether patients for whom it was intended received the intervention. High and low performing practices were invited to participate in semi-structured interviews. The CFIR guided interview development as it defined specific types of environmental influences on EBP implementation, including constructs from each domain, including the outer setting, inner setting, intervention characteristics, individual characteristics, and process. Both practice staff and members of the PCT were interviewed separately. After completing thematic coding, the valence and strength of each response was rated and mapped to each construct.

**Findings:** 80% of the 33 practices scored between 40-70% in primary fidelity, indicating the majority of them were using the new resource to some degree. Secondary fidelity revealed that only 11 practices were using the resource as it was intended for their high-risk patient cohort. Interviews revealed that aspects of the process were described as a positive facilitator to practice staff but a negative influencer to the PCT while centralization emerged as disparate but in the opposite way. Common barriers expressed included not enough of the resource available for the patient need, lack of engagement of stakeholders, and role confusion/lack of clarity. Common facilitators were good working relationships, easily accessible, and enhanced expertise improving care delivery to patients.

**Implications for D&I Research:** A mixed methods approach in implementation science can inform stakeholders if course corrections are needed to yield success. Additionally, development of divergent fidelity measures helps to illuminate more granularly, where opportunities lie for implementation improvement.

### S-42 Mixed-methods economic evaluation of the implementation of tobacco treatment programs within NCI-designated cancer centers

#### Ramzi Salloum^1^, Heather D'Angelo^2^, Ryan Theis^1^, Jennifer LeLaurin^1^, Danielle Pauk^3^, Li-Shiun Chen^4^, Andrew Day^5^, Adam Goldstein^6^, Brian Hitsman^7^, Deborah Hudson^8^, Andrea King^9^, Cho Lam^10^, Arnold Levinson^11^, Judith Prochaska^12^, Susanne Tanski^13^, Kathryn Taylor^14^, Janet Thomas^15^, Hilary Tindle^16^, Elisa Tong^17^, Graham Warren^18^, Timothy Baker^19^, Michael Fiore^20^

##### ^1^University of Florida College of Medicine, Gainesville, FL, USA; ^2^National Institutes of Health, National Cancer Institute, Rockville, MD, USA; ^3^University of Wisconsin-Madison, Madison, WI, USA; ^4^Psychiatry, Washington University School of Medicine, St. Louis, MO, USA; ^5^University of Texas Southwestern Medical Center, Dallas, TX, USA; ^6^University of North Carolina at Chapel Hill, Chapel Hill, NC, USA; ^7^Northwestern University, Chicago, IL, USA; ^8^Indiana University, Indianapolis, IN, USA; ^9^University of Chicago, Chicago, IL, USA; ^10^University of Utah, Salt Lake City, UT, USA; ^11^University of Colorado Cancer Center, Aurora, CO, USA; ^12^Stanford Cancer Institute, Stanford, CA, USA; ^13^Dartmouth University, Lebanon, NH, USA; ^14^Georgetown University, Washington, DC, USA; ^15^University of Minnesota, Minneapolis, MN, USA; ^16^Vanderbilt Center for Tobacco, Addiction, and Lifestyle (ViTAL), Nashville, TN, USA; ^17^Internal Medicine, University of California, Davis, Sacramento, CA, USA; ^18^Medical University of South Carolina, Charleston, SC, USA; ^19^University of Wisconsin, Center for Tobacco Research and Intervention, Madison, WI, USA; ^20^School of Medicine & Public Health, University of Wisconsin, Center for Tobacco Research and Intervention, Madison, WI, USA

###### **Correspondence:** Ramzi Salloum (rsalloum@ufl.edu)

**Background:** The Cancer Center Cessation Initiative was launched in 2017 as part of the NCI Cancer Moonshot program to assist cancer centers in developing tobacco treatment programs (TTPs) for all oncology patients who smoke. Forty-two participating centers have implemented unique evidence-based TTPs that fit their institutional resources and needs, offering a variety of components including quitline referrals, in-person and/or telephone-based counseling, integrated voice response systems, web- and text-based services, and medications.

**Methods:** We used a mixed methods comparative case study design to evaluate 14 participating cancer centers that retrospectively reported implementation costs from a health-system perspective for 1 six-month period between July 2018 and December 2019. We analyzed operating costs by resource category (e.g., personnel), cost-per-participant (i.e., reach) and cost-per-quit (i.e., effectiveness), concurrently with transcripts from key-informant interviews conducted during site visits to identify relevant contextual factors. Personnel salary costs were estimated using Bureau of Labor Statistics wage data adjusted for area and occupation, adding 30% for benefits.

**Findings:** The centers varied considerably in terms of TTP components including staffing levels, technology, and the provision of medications, education and/or training materials. Total monthly operating costs across funded centers varied from $517 to $20,495. The largest operating cost category was personnel ($3,955-$19,555), with the highest costs attributable to the provision of in-person TTP services. Monthly costs ranges for other categories were: medications ($50-$451), materials ($7-$487), training ($113-$1,000), technology ($250-$2,750), and equipment ($191-$1325). Cost-per-quit ($155) and cost-per-participant ($2) were lowest in a point-of-care program that reported $0 personnel costs. Four programs achieved similar cost-per-quit ($2,097-$3,180) using different combinations of TTP components, ranging from individually-delivered in-person counseling only to multiple components.

**Implications for D&I Research:** Among centers that have progressed in TTP implementation, the cost-per-quit was modest relative to other health interventions. Although some centers have achieved similar average costs by offering TTP components with varying levels of intensity, these programs have diverged widely in their reach and effectiveness. Evaluating implementation costs alongside reach and effectiveness provides decision makers in oncology with the important additional information needed to optimize resource allocation when establishing tobacco treatment services and tailoring them to their unique settings.

**Primary Funding Source:** National Institutes of Health

### S-43 Strategies for financing the implementation of evidence-based practices: A scoping review

#### Alex Dopp

##### RAND Corporation, Santa Monica, CA, USA

###### **Correspondence:** Alex Dopp (adopp@rand.org)

*Background*: Increased availability of evidence-based practices (EBPs) is essential to alleviating the public health and societal impact of health problems. A major challenge to implementing EBPs is the limited and fragmented nature of available funding.

*Methods*: We conducted a scoping review (Dopp et al., in press) assessing the current state of evidence on EBP financing strategies based on recent literature (i.e., post-Affordable Care Act). The review focused on behavioral health literature, but identified strategies that can used broadly in health care. We defined financing strategies as techniques that secure and direct financial resources to support EBP implementation. We used the review to create a compilation of identified financing strategies, following established reporting guidelines for implementation strategies.

*Findings*: Our review identified 23 financing strategies. Examples of strategies reported being used in the literature reviewed include increased fee-for-service reimbursement, capitated payments, pay-for-performance, and pay-for-success contracts. Available research did not permit strong conclusions about the impact of financing strategies on EBP implementation outcomes. We were able to characterize their level of reported use in behavioral health literature (13 current use, 9 limited/potential use, 1 potentially contraindicated); however, many financing strategies not currently used in behavioral health are likely applied more widely in other health care sectors.

***Implications for D&I Research:*** The existing literature on EBP financing strategies raises far more questions than answers. Therefore, we propose a research agenda that will help better understand financing strategies by (1) advancing methods to evaluate financing strategies, (2) partnering with stakeholders and decision-makers, (3) focusing on high-need strategies and service systems, (4) guiding selection of financing strategies, and (5) greater attention to sustainable financing. We will also discuss the implications of our findings for health professionals, system leaders, and policymakers who want to develop robust, sustainable financing for EBP implementation.

References:

Dopp AR, Narcisse MR, Mundey P, Silovsky JF, Smith AB, Mandell D, Funderburk BW, Powell BJ, Schmidt S, Edwards D, Luke D, Mendel P. A scoping review of strategies for financing the implementation of evidence-based practices in behavioral health systems: State of the literature and future directions. *Implementation Research and Practice*. Manuscript in press.

**Primary Funding Source:** Implementation Research Institute

### S-44 Pay for success financing: A strategy to scale multisystemic therapy in Colorado

#### Suzanne Kerns

##### University of Denver, Denver, CO, USA

###### **Correspondence:** Suzanne Kerns (Suzanne.Kerns@du.edu)

*Background*: Pay for Success (PfS) financing enables a government to partner with social impact investors to fund a project based on measurable positive outcomes. A government identifies a social problem and a service provider offers a potential solution (e.g., scale a specific evidence-based practice; EBP). Foundations or other impact investors provide upfront capital to the service provider, who then delivers services to the identified population. An independent evaluator measures predetermined success indicators. If found to be successful, the government repays investors with interest. Thus, PfS financing is one strategy to balance financial risks associated with EBP implementation. PfS financing remains an underutilized strategy to support scaling of EBPs.

***Methods:*** In the Colorado-based PfS project, reducing juvenile justice recidivism due to chronic antisocial behavior (e.g., conduct disorder) was the priority problem identified by the State of Colorado. Multisystemic Therapy (MST) was chosen as the behavioral health EBP to address this problem. The Center for Effective Interventions is an intermediary organization that manages the core PfS contract and supports “flow through” to local community-based mental health centers delivering MST. Success payments are predicated on 1) intermediate indicators of successful implementation (i.e., fidelity ratings), and 2) primary outcomes of reductions in youth recidivism and out-of-home placement compared to matched controls.

*Findings*: Six MST teams (across three cohorts) were implemented over an 18 month period. Between January 2019 and June 2020, the first cohort has served 95 youth. Between July 2019 and June 2020, the second cohort has served 53 youth. The third cohort launched in July 2020. Initial fidelity benchmarks were met, and thus the first repayment to the investors was made. This project is still underway, with final results anticipated by January 2022.

***Implications for D&I Research:*** Research on PfS financing introduces unique opportunities for studying multi-system partnerships and high-need service systems. The additional oversight and accountability inherent in PfS financing creates distinctive presses and pulls for participating community mental health agencies. Substantial transaction costs are associated with setting up this financing mechanism and fulfilling the requirements. Investigating these complexities will help realize the potential of PfS financing to support scaling of EBPs with consideration to long-term sustainability.

**Primary Funding Source:** Colorado State Pay for Success Initiative

### S-45 Translating economic evaluations into financing strategies: A case study of a multi-component alternative payment model for evidence-based oncology care

#### Laura Panattoni

##### Fred Hutchinson Cancer Research Center, Seattle, WA, USA

###### **Correspondence:** Laura Panattoni (lpanatto@fredhutch.org)

*Background*: In 2016, the Centers for Medicare and Medicaid Services (CMS) launched the 5-year Oncology Care Model (OCM), its first initiative to transition from fee-for-service payments to value-based purchasing in oncology. The OCM tests whether an innovative payment strategy can improve quality and lower costs to Medicare. We discuss the role economic evaluation played in the development of the OCM and a research agenda to advance alternative payment strategies in oncology.

***Methods:*** We conduct a case-study based on expert knowledge of the OCM. The OCM requires practices to provide the following evidence-based ‘enhanced services’: 24/7 access to a clinician, patient navigation, a care plan, and guideline based treatment. The provision of some enhanced services is underfunded by fee-for-service. The OCM utilizes fee-for-service payments plus a per-beneficiary $160 Monthly Enhanced Oncology Services (MEOS) Payment and performance-based incentive payments (PBP).

*Findings*: OCM’s payment mechanisms were informed by successful demonstration projects. However, a recent systematic review of 22 alternative payment strategies in oncology concluded that there was weak evidence for their efficacy. The $160 MEOS payment was calculated based on the estimated staffing required to provide the enhanced services and did not account for implementation costs. A more recent oncology alternative financing strategy has proposed MEOS payments of $225-$675. Resource use by OCM practices will hopefully shed light on the implementation and sustainment costs of enhanced services. A simulation analysis examined the budget impact to Medicare and practices and found that the MEOS payment is a small portion of Medicare’s total spending but it is large relative to the average fee-for-service revenues of an oncology practice. The analysis suggests practices would have to reduce utilization by roughly 4 percent for Medicare to ‘break even’. However, this analysis did not explicitly model the PBP quality metrics, which may have low reliability in small oncology practices.

***Implications for D&I Research:*** Despite extensive development efforts, it has proven a challenge to execute and evaluate the OCM in ways that demonstrably impact implementation and clinical outcomes. Future research is needed to develop alternative payment strategies that fairly reimburse practices for implementing evidence-based oncology care and support the economic sustainability of evidence-based practice.

### S-46 Associations between broadband access among rural/urban cancer patients and completion of electronic patient reported outcomes (ePROs) in a stepped-wedge cluster-randomized pragmatic trial (EHR-facilitated Cancer Symptom Control (E2C2))

#### Joan Griffin^1^, Kristin Fischer^2^, Jennifer Ridgeway^2^, Lila Finney Rutten^2^, Aaron Leppin^2^, Amanda Nelson^2^, Nathan Tesch^2^, Kathryn Ruddy^2^, Sarah Redmond^2^, Diedre Pachman^2^, Casey Fazer^2^, Margaret Wagnerowski^2^, Amy Johnson^2^, Andrea Cheville^2^

##### ^1^Mayo Clinic College of Medicine, Rochester, MN, USA; ^2^Mayo Clinic, Rochester, MN, USA

###### **Correspondence:** Joan Griffin (Griffin.Joan@mayo.edu)

**Background:** Rural cancer survivors experience greater disparities in cancer-related symptom management than urban-dwelling patients. Disparities can partly be explained by distance or access to healthcare, specialty care and social networks. Remote symptom monitoring and management systems may lessen disparities for rural patients, but electronic patient-reported outcome surveys (ePROs) used in these systems may be differentially adopted by rural patients, especially those with limited access to broadband services. To evaluate the equitable implementation of a novel EHR-enabled symptom monitoring and management system in the Midwest U.S., we examined differences in ePRO completion by residence and broadband access.

**Methods:** In the Enhanced, EHR-facilitated Cancer Symptom Control (E2C2) system, patients receive ePROs for sleep, pain, anxiety, depression, and fatigue symptoms and limitations in physical function in their patient portal. They can respond electronically or in-clinic via tablet. ePRO responses trigger evidence-based symptom-management approaches: automated self-management resources for patients reporting moderate symptoms/limitations and nurse-managed collaborative care for patients reporting severe symptoms and/or functional limitations.

As part of a Hybrid II stepped-wedge cluster-randomized pragmatic trial, we evaluated E2C2 “intervention reach” (i.e., ePRO response rates, response mode, access to patient portal, and portal use) for patients by urban or rural residence. Residence was also classified as being in a community with high (≥85% of households) or low (<85% of households) likelihood of broadband access (2018 American Community Survey zip code estimates).

**Findings:** From the first block of randomized clinical sites, urban patients were significantly more likely than rural patients to reside in communities with high broadband access (44% vs. 3%, p<.01.) Living in an area with higher levels of broadband access was significantly associated with patient portal activation (87% vs. 80%, p<.001) and higher ePRO response via the patient portal (62% vs. 48%, p<.001).

**Implications for D&I Research:** In an era where remote, web-based approaches to implementation are increasingly promoted and relied upon, our findings suggest web-based only approaches for symptom management may result in intervention reach and adoption disparities, and ultimately health outcomes. Efforts are needed to develop and target varied strategies, particularly in areas with low broadband access, which meet different needs and circumstances of individuals equitably.

**Primary Funding Source:** National Institutes of Health

### S-47 Implementation of a behavioral economics electronic health record (BE-EHR) module to optimize diabetes management in older adults

#### Hayley Belli^1^, Andrea Troxel^1^, Saul Blecker^2^, Judd Anderman^2^, Christina Wong^2^, Tiffany Martinez^1^, Devin Mann^2^

##### ^1^New York University School of Medicine, New York, NY, USA; ^2^NYU Langone Health, New York, NY, USA

###### **Correspondence:** Hayley Belli (hayley.belli@nyulangone.org)

**Background:** The integration of behavioral economics (BE) principles and electronic health records (EHRs) using clinical decision support (CDS) tools is a novel approach for delivering clinical care and improving health outcomes. Few studies to date have attempted to combine BE, EHR, and CDS approaches to manage chronic conditions and address the psychology of de-prescribing, specifically in diabetes where intensive glycemic control is of unclear benefit and carries increased risk for older adults. The American Geriatrics Society’s *Choosing Wisely* (CW) guideline promotes less aggressive glycemic targets and reduction in pharmacologic therapy for older adults with type II diabetes.

**Methods:** We developed and pilot tested an intervention that leverages BE with EHR technology and CDS tools to reduce overtreatment of type II diabetes in older adults (age 76+). The BE-EHR module was developed through a series of design workshops, interviews, user-testing sessions, and clinic observation visits. The final intervention comprised six components (nudges) that incorporate BE principles including framing, social norming, accountable justification, defaults, affirmation, and gamification. The module was successfully integrated into the New York University Langone Health (NYULH) EHR (Epic) for pilot testing in five NYULH clinics from July 12, 2018 through October 31, 2019.

**Findings:** The primary study outcome was patient-level CW compliance defined by a patient’s life expectancy and target HbA1c according to the CW guideline. Results indicated an average increase in CW compliance of 5.1% from a 16-week interval at baseline (1,278 patients; Mean [95% CI]: 16.1% [14.1%, 18.1%]) to a 16-week interval at the conclusion of the pilot study (680 patients; 21.2% [18.1%, 24.3%]). Implementation outcomes including acceptability, adoption, and reach among clinicians were also measured. Individual BE-EHR module components activated between 573-3,445 times during the pilot study.

**Implications for D&I Research:** The BE-EHR module shows promise for promoting the CW guideline and improving diabetes management in older adults. The approach of leveraging BE with EHRs and CDS tools has potential to be applied to other health outcomes, and is a highly scalable intervention. Currently, a pragmatic, cluster-randomized controlled trial is underway to test the effectiveness of the intervention vs. usual care across 66 NYULH clinics.

**Primary Funding Source:** National Institutes of Health

### S-48 Identifying immunization information system (IIS) implementation factors in the independent community pharmacy setting

#### Tessa Hastings^1^, David Ha^2^, Brent Fox^3^, Jingjing Qian^3^, Joni Lakin^4^, Salisa Westrick^3^

##### ^1^University of South Carolina, Columbia, SC, USA; ^2^Stanford Health Care, Stanford, CA, USA; ^3^Auburn University, Harrison School of Pharmacy, Auburn, AL, USA; ^4^Auburn University, Auburn, AL, USA

###### **Correspondence:** Tessa Hastings (hastint@mailbox.sc.edu)

**Background:**

Immunization Information Systems (IISs) consolidate vaccination information for individuals within a defined geographic area. This information is critical for immunization providers to make strong recommendations at the point of clinical care, and to monitor vaccination rates and facilitate outbreak response efforts at the population level. However, pharmacy participation is limited in many states, like Alabama, where reporting is not mandatory and especially among independent pharmacies with limited resources compared to their larger chain counterparts. The objective of this study was to describe implementation factors that influence independent community pharmacy non-mandatory participation in IISs.

**Methods:**

A 68-item questionnaire was administered to 33 independent community pharmacists in Alabama participating in a randomized controlled trial to improve IIS enrollment and participation. Likert-type items were mapped to the Consolidated Framework for Implementation Research (CFIR) domains: 1) IIS Characteristics (25 items), 2) Individual Characteristics (3 items), 3) Inner Setting (21 items), 4) Outer Setting (6 items), and 5) Process (13 items). Subscales were identified using exploratory factor analysis. Mean scale scores were calculated for each overall domain and subscale with scores ranging from 0 to 6. Hierarchical linear regression was used to explore the relationship between implementation factors and IIS participation. Participation was defined as proportion of self-reported doses administered that were also recorded in the IIS.

**Findings:**

IIS Characteristics had the highest score (4.98), followed by Inner Setting (4.92), Process (4.71), Characteristics of Individuals (4.67), and Outer Setting (4.51). Implementation factors were associated with participation in IIS, *R*^2^ = 0.599,*F*= 3.884,*p* = 0.023. Ease of Use was a predictor of participation (p=0.038), *R*^2^ = 0.419,*F*= 3.599,*p* = 0.039. Additionally, Process was associated with IIS participation, *R*^2^ = 0.430,*F*= 6.043,*p* = 0.011, with the Executing subscale being a significant predictor (p=0.003). There was a significant difference in Readiness for Implementation (p=0.003) between intervention and control groups three months post-intervention.

**Implications for D&I Research:**

Pharmacy participation in IISs is critical to safe, effective, and efficient provision of immunizations. Implementation Factors were found to predict participation in the IIS. These results may be used to inform the selection and/or development of future strategies to improve IIS adoption and utilization in independent community pharmacies.

**Primary Funding Source:** Agency for Healthcare Research and Quality

### S-49 Economic analysis of a remote patient monitoring program for post-discharge patients with type 2 diabetes

#### Tzeyu Michaud, Paul Estabrooks, Jennie Hill, Dejun Su

##### University of Nebraska Medical Center, Omaha, NE, USA

###### **Correspondence:** Tzeyu Michaud (tzeyu.michaud@unmc.edu)

**Background**: Less than a quarter of all published telehealth studies contain quantitative cost data or make reference to the costs of telehealth. It is important to understand the costs of implementing technology-facilitated disease management programs to determine program sustainability and potential for future adoption. This study aimed to assess the cost and cost-effectiveness of implementing a remote patient monitoring (RPM) program to enhance the management of type 2 diabetes.

**Methods**: Using retrospective data collected during RPM implementation from September 2014 to February 2018, we estimated the costs of implementing a RPM program for post-discharge diabetes management in the primary care setting. Measures included total and average annual costs, costs per participant enrolled or completed, costs per person-day, and cost-effective ratios. We took the perspective of a healthcare system to assess the prospect of sustaining the program after grant funding creased. We further conducted sensitivity and scenario analyses (SA) to examine variations in estimated program costs associated with varying program efficiencies and alternative personnel compositions of the RPM team.

**Findings**: Altogether 182,932 person-days were covered during the 3.5 years of the RPM program. The total RPM implementation costs were estimated at $4,210,047 with an average annual program costs of $1,202,871, which translated to $3,087 per participant completing the three-month program. The per person-day cost was averaged at $23.The cost-effectiveness ratio was $5,512 for each unit decrease in hemoglobin A1c level. SA results indicate that the sustainment costs were approximately $1.5 million annually and the per-person-day costs were between $20 and $26 with each nurse coach on average serving a panel of 61-91 patients.

**Implications for D&I Research:** Estimating the cost of implementing and operating an RPM program is essential in justifying its adoption in the context of budget constraints for stakeholders. This study presents an economic case with implementation and sustainment costs, and cost-effectiveness metrics of an RPM-facilitated diabetes management program to aid informed decision for healthcare organizations related to program adoption and sustainability. The daily reimbursement rates, estimated by costs per person-day managed, shed some lights in terms of the program efficiency and service coverage for the potential RPM service payment.

### S-50 Partnered innovation to implement timely and personalized care: A case study

#### Cole Hooley^1^, Enola Proctor^2^, Virginia Mckay^3^, Emre Toker^4^, Rebecca Lengnick-Hall^5^, Thomas Maddox^6^

##### ^1^Brigham Young University, Provo, UT, USA; ^2^George Warren Brown School of Social Work, Washington University in St. Louis, St. Louis, MO, USA; ^3^School of Social Work, Washington University in St. Louis, St. Louis, MO, USA; ^4^Washington University in St. Louis, St. Louis, MO, USA; ^5^washington university in st. louis, St. Louis, MO, USA; ^6^BJC Health System, St. Louis, MO, USA

###### **Correspondence:** Cole Hooley (cole_hooley@byu.edu)

**Background:**

A growing number of healthcare systems have established formal innovation labs to shape and implement service delivery improvements. Little research has examined the role of innovation centers and entrepreneur partnerships in implementation efforts. This study aims to: understand how innovative companies, health systems, and their leaders partner to design and implement innovations into care delivery; characterize decision points, information and data sources; and identify the challenges and risks in partnering for viable uptake and scale-up of innovation.

**Methods:**

Through a CTSA project on the implementation science-entrepreneurship interface, we conducted a case study to understand how organizations collaborated to co-create and implement an innovation designed to improve the timeliness and patient-centeredness of cardiovascular care. Three organizations were part of the collaboration: a private innovative startup company; a health system innovation lab; and a cardiovascular clinic. Researchers conducted four focus groups with key informants from each organization from November 2019- January 2020. Focus groups were audio recorded, transcribed verbatim, and manually checked for accuracy. Using a case analysis approach, all members of the transdisciplinary research team iteratively worked to condense and synthesize data in an iterative fashion.

**Findings:**

The collaboration proceeded through the following phases: early exploration of the partnership, formalizing the partnership, co-creating the innovation, piloting the innovation, and planning for scale-up. Phases were punctuated by clear decision points such as forming the partnership, negotiating terms of the partnership, iterating form and features of the innovation, and determining if value-add of the innovation is sufficient to promote scale-up and sustainment. As the organizations’ leaders described their aims and processes, key implementation concepts were apparent, including implementation strategies (e.g., champions and iterative trialing) and the implementation outcomes of acceptability, sustainment, and scale-up. Participants also described the potential risks of collaboration, reflected on their co-creation process, and the value of engaging stakeholders in innovation design.

**Implications for D&I Research:**

Results describe a process, decisions points, and lessons learned that could inform subsequent collaborations between entrepreneurs and implementation scientists in healthcare systems. They further reveal how health system leaders make decisions about shaping and implementing innovations to improve care.

**Primary Funding Source:** National Institutes of Health

### S-51 Getting to implementation: A manualized adaptation of getting to outcomes for healthcare settings selecting implementation strategies

#### Shari Rogal^1^, Vera Yakovchenko^2^, Sharon McCarthy^3^, Byron Powell^4^, Brittney Neely^1^, Lisa Lederer^1^, Shauna McInnes^1^, Matthew Chinman^1^

##### ^1^VA Pittsburgh Healthcare System, Pittsburgh, PA, USA; ^2^Veterans Health Administration, BridgeQUERI & CHOIR, Bedford, MA, USA; ^3^Veterans Integrated Service Network 4 Mental Illness Research, Education and Clinical Center, VA Pittsburgh Healthcare System, Pittsburgh, PA, USA; ^4^Washington University in St. Louis, St. Louis, MO, USA

###### **Correspondence:** Shari Rogal (rogalss@upmc.edu)

**Background:** Getting To Outcomes (GTO) is an evidence-based, multi-step approach that has been used to improve organizations’ planning, implementing and evaluating community-based programs. GTO employs a learn-by-doing approach, site-expert collaborations, and stakeholder-engaged decision making. While GTO has been adapted for different evidence-based community interventions, it has not been adapted as a tool for selecting and tailoring implementation strategies. Therefore, we aimed to systematically adapt GTO, developing Getting To Implementation (GTI), a user-friendly tool for healthcare settings focusing on the uptake of evidence-based practices.

**Methods:** The Framework for Reporting Adaptions and Modifications-Expanded (FRAME) was used to specify adaptations made to GTO. Adaptations were *planned/proactive* and occurred in the *planning/pre-implementation phase*. Decisions were made *collaboratively* by the study team (including GTO developers), healthcare system leaders, systems engineers, and subject matter experts. Generic GTI was then tailored for a national program aiming to improve cirrhosis care, informed by clinician implementation strategy surveys from 100 VA facilities and semi-structured interviews with 12 of the highest performing facilities in VA cirrhosis care.

**Findings:** Modifications were made to GTO’s *context, content, training,* and *evaluation* to produce GTI. While GTO is agnostic to setting, its *context* was adapted such that GTI specifically supports evidence-based practices in healthcare settings. While the core GTO *content* was preserved, the manual *packaging* was minimized for busy healthcare workers and steps were condensed and expanded to accommodate the change in focus from intervention to implementation. GTI *training* was likewise condensed from one week to an intensive two-day session for busy clinicians. *Evaluation* was intensified to include real-time tracking of implementation activities and regular check-ins, given the extent of adaptations. The generic GTI structure was subsequently tailored for implementing evidence-based cirrhosis care at VA medical centers by integrating cirrhosis-specific details and instructions for choosing a bundle of implementation strategies based on strategy survey findings.

**Implications for D&I Research:** GTO was adapted, with fidelity, to create GTI for cirrhosis care in healthcare settings. The generic version of GTI can be tailored to help practitioners design, select, and tailor implementation strategies across a range of domains.

**Primary Funding Source:** Department of Veterans Affairs

### S-52 Practical EHR export pathways (PEEP): Rapid implementation of automated data abstraction for a global COVID-19 registry using the dynamic sustainability framework

#### Smith Heavner-Sullivan^1^, Catherine Chen^2^, Yasir Tarabichi^3^, Trayson Llano^1^, Laura Rolke^4^, Mackenzie Stuenkel^4^, Dakotah Insley^1^, Sarah Griffin^4^, Valerie Danesh^5^, Alena Guenther^6^, Namrata Patil^7^, Amr Abdelaty^8^, Sreekanth Cheruku^2^, Omar Mehkri^9^, Joshua Denson^10^, Aaron Gillet^10^, Prerana Roth^1^, Alain Litwin^1^, Karen Boman^11^, Vikas Bansal^12^, Vishakha Kumar^11^, Rahul Kashyap^12^

##### ^1^Prisma Health, Greenville, SC, USA; ^2^UT Southwestern, Dallas, TX, USA; ^3^MetroHealth Medical Center, Cleveland, OH, USA; ^4^Clemson University, Clemson, SC, USA; ^5^University of Texas at Austin, Austin, TX, USA; ^6^Allina Health, Minneapolis, MN, USA; ^7^Brigham and Women's Hospital, Boston, MA, USA; ^8^Englewood Health, Englewood, NJ, USA; ^9^Cleveland Clinical Foundation, Cleveland, OH, USA; ^10^Tulane University School of Medicine, New Orleans, LA, USA; ^11^Society of Critical Care Medicine, Mount Prospect, IL, USA; ^12^Mayo Clinic, Rochester, MN, USA

###### **Correspondence:** Smith Heavner-Sullivan (smitty.heavner-sullivan@prismahealth.org)

**Background:**

The SCCM’s Critical Care Network, Discovery, has led the Viral Infection and Respiratory Illness Universal Study (VIRUS) in creating a global COVID-19 registry for real-time data gathering and analytics and rapid dissemination of treatments and outcomes. Guided by the dynamic sustainability framework (DSF), 38 health system sites are collaborating in rapidly developing tools and resources to optimize electronic health record (EHR) data collection. This VIRUS subcommittee is called Practical EHR Export Pathways (PEEP).

**Methods:**

Recognizing this work as an “intervention,” PEEP sites began an iterative process, with multiple sites producing and rapidly distributing resources (e.g., Excel templates, SAS code), incorporating feedback to describe successful tools, to optimize fit to practice settings and the ecological system as defined in DSF, and to identify key constructs by consensus. PEEP-prioritized resources specific to the Epic platform leverage standard search queries to obtain structured data from common EHR-generated relational databases and facilitate peer-to-peer coaching on the development and execution of end-user Epic reporting functions. PEEP sites report implementation progress and validity through an online form and in biweekly team meetings.

**Findings:**

Ten of 38 PEEP sites automated over 80% of VIRUS registry variables within 9 weeks. Key intervention characteristics included adaptability, trialability, and complexity. Successfully fitting the intervention required access to necessary platforms, knowledge of local data structure, and insight into clinical culture and documentation practices. At 3 sites reporting more than 90% automation, gathering data for VIRUS was a core operational priority.

Sites (n=10) have reported greater than 90% validity of automated data, with each undertaking an iterative process of adaptation and continuous quality improvement. A multisite, longitudinal data validation study is evaluating sustainability (expected by October 2020). Expansion and replication of PEEP procedures are extending to the Cerner EMR platform.

**Implications for D&I Research:**

DSF guided the rapid creation of a COVID-19 registry in response to the COVID-19 pandemic. The registry’s development and its methodology for sustainability provide critical information to improve intervention, practice, and ecological outcomes. This work will inform future infectious disease response, maximizing response through D&I methodology.

## Global Dissemination and Implementation Science

### S-53 Evaluating the integrative systems praxis for implementation research (INSPIRE) methodology: Implementation of an HPV-based screen and treat cervical cancer prevention program in the peruvian Amazon

#### Patti Gravitt^1^, Anne Rositch^2^, Batel Blechter^2^, Graciela Meza^3^, Margaret Kosek^4^, Lita Carillo^5^, Valerie A. Paz-Soldan^6^

##### ^1^University of Maryland School of Medicine, Baltimore, MD, USA; ^2^Johns Hopkins Bloomberg School of Public Health, Baltimore, MD, USA; ^3^Universidad Nacional de la Amazonia Peruana, Iquitos, Peru; ^4^University of Virginia, Charlottesville, VA, USA; ^5^DIRESA-Loreto, Iquitos, Peru; ^6^Tulane University School of Public Health and Tropical Medicine, Lima, Peru

###### **Correspondence:** Patti Gravitt (PGravitt@som.umaryland.edu)

**Background:** The INSPIRE methodology uses a participatory, systems thinking approach to facilitate resource adaptation and implementation of evidence-based interventions (EBIs) through iterative phases of contextual system understanding, finding leverage for change, acting, learning and adapting.

**Methods:** Multilevel stakeholders from the Micro Red Iquitos Sur health network (Iquitos, Peru) participated in facilitated group model building ‘design’ workshops (DWs). Using a combination of knowledge transfer, deliberative dialogue, and scenario analyses, a shared decision was reached to change cervical cancer screening from a visual screen and referral algorithm (VIA) to a molecular HPV-based screen and treat algorithm. Stakeholder working groups developed service delivery plans according to available resources and typical system processes. The following screening and management indicators were evaluated for 18 months pre- and 8 months post-implementation of HPV-based screening using the RE-AIM framework: REACH: percent of women screened (goal: 70% coverage of screen-eligible population); EFFECTIVENESS: percent of screen-positive women completing care; ADOPTION: percent of health facilities using HPV testing, percent of self-collected samples; IMPLEMENTATION: reduced time-in-system.

**Findings:** Average screening rates increased from 21.9% (85.3 tests/month) to >100% (278.5 tests/month) of monthly screening goals in the post-implementation (p=0.004). As of February 2020, 220/336 (60.1%) of HPV-positive women had been evaluated for ablative therapy eligibility; 199 (90%) were eligible and all received treatment by thermal ablation. In contrast, 53.2% of women testing positive by VIA- or Pap had no record of colposcopy and only 38.4% of pre-implementation screen-positive women reached a completion of care endpoint by February 2020. Most samples (62.8%) were self- vs. clinician-collected with similar HPV prevalence (19.5% and 18.4%, respectively). All 17 health facilities adopted HPV testing and de-implemented VIA-based testing within 6-months of program initiation. The average time-to-treatment was reduced from several months to <30 days.

**Implications for D&I Research:** Application of the INSPIRE methodology for context adaptation of evidence-based cervical cancer screening and management ensured stakeholder buy-in and integration of services into existing health system processes, resulting in rapid scale-up across 17 health facilities. Next steps are to expand to other health networks to further evaluate the effectiveness of integrated systems thinking approaches for regional scale-up and sustainability of EBIs in complex health systems.

**Primary Funding Source:** National Institutes of Health

### S-54 Learning needs assessment for multi-stakeholder implementation science training in LMIC settings: Findings and recommendations

#### Mallory Turner^1^, Stephanie Bogdewic^1^, Erum Agha^2^, Carrie Blanchard^1^, Rachel Sturke^3^, Audrey Pettifor^1^, Kathryn Salisbury^1^, Andrea Marques^4^, Rohit Ramaswamy^1^

##### ^1^University of North Carolina, Chapel Hill, Chapel Hill, NC, USA; ^2^University of North Carolina at Chapel Hill School of Medicine, Chapel Hill, NC, USA; ^3^National Institutes of Health, Fogarty International Center, Bethesda, MD, USA; ^4^National Institutes of Health, National Institute of Mental Health, Bethesda, MD, USA

###### **Correspondence:** Mallory Turner (malwolfe@email.unc.edu)

**Background:**

With the global growth of implementation science (IS), the need to build the capacity of various stakeholders has become a priority, particularly in low- and middle-income countries (LMICs). Current training programs are mostly available for researchers in high income settings. Participants in these programs have indicated that LMIC specific curricula and training programs customized to the local needs of researchers, practitioners and policy makers are urgently required. This study seeks to address this gap by presenting results from an IS learning needs assessment conducted across various LMIC stakeholders.

**Methods:**

We asked a purposively sampled group of IS experts to enumerate IS stakeholders and then reviewed the IS/KT literature to define a final stakeholder group list. Forty semi-structured interviews were used for the needs assessment and study participants were recruited through three global IS networks. Interested participants were asked to assign themselves to a stakeholder group and interviewees were selected using stratified random sampling. Employing principles of double-loop learning, we conducted deductive thematic analysis using the interview guide as a framework, followed by a reflexive analysis to highlight assumptions about LMIC contexts made during analysis. Themes and assumptions were independently validated by external assessors with lived experience in LMICs.

**Findings:**

Preliminary results suggest that stakeholders vary widely in their perceptions of IS and identified the need for fundamental, widespread, and context-specific training and increased capacity for conducting basic operational research. Stakeholders requested interdisciplinary content and mixed online and in-person delivery by instructors familiar with LMIC contexts. Challenges to utilizing IS methods and frameworks “imported” from different contexts and funded by donors in high-income countries with little understanding of local context in LMICs was a dominant theme. Our results show that many researchers in LMICs first need IS training combined with basic research skills strengthening, whereas practitioners need content, delivery methods and instructors who can help them address problems in their own countries.

**Implications for D&I Research:**

Continued development and testing of learning programs for LMIC settings based on this assessment is needed, and the relevance of the current body of knowledge to low-income settings should be evaluated.

**Primary Funding Source:** National Institutes of Health

### S-55 Participatory action research and rapid ethnography to increase coverage of mass drug administration in Benin

#### Arianna Means^1^, Kevin Bardosh^2^, Euripide Avokpaho^3^, Benon Monra^4^, Aubierge Kpatinvoh^5^, Emmanuel Sambieni^4^, Moudachirou Ibikounlé^6^

##### ^1^Department of Global Health, University of Washington, Seattle, WA, USA; ^2^University of Washington, Seattle, WA, USA; ^3^Institut de Recherche Clinique du Bénin, Abomey-Calavi, Benin; ^4^University of Parakou, Parakou, Benin; ^5^University of Abomey-Calavi, Contonou, Benin^6^Université d’Abomey-Calavi, Cotonou, Benin

###### **Correspondence:** Arianna Means (aerubin@u.washington.edu)

**Background:**

Neglected tropical diseases (NTDs) are a group of disabling conditions that cause severe morbidity and income loss. One of the primary methods used to control NTDs is mass drug administration (MDA), in which entire endemic communities are targeted with treatment. However, the impact of MDA is predicated on achieving high treatment coverage and, in many settings, coverage of MDA amongst specific populations remains low. The purpose of this project is to determine if engaging communities in the development of effective and feasible implementation strategies will improve treatment coverage of hard-to-reach populations.

**Methods:**

This study takes place in two districts in Benin, an intervention and a control district. We use participatory action research and rapid ethnography to learn about community member perceptions of MDA programs and identify who is missed by MDA, best practices for reaching these individuals, and different delivery strategies that might be most effective in reaching specific sub-groups. Rapid ethnography findings are shared with Ministry of Health NTD leaders during a participatory co-design meeting, and a structured nominal group technique is used to derive implementation strategies most likely to increase treatment coverage. The new implementation strategy is delivered during community outreach prior to the next round of MDA, and a difference-in-differences design is used to determine if the newly devised implementation strategy effectively increased coverage relative to the control district.

**Findings:**

Key barriers to MDA participation identified during rapid ethnography include mistrust of community health workers delivering MDA, poor penetration of MDA programs into rural or geographically isolated areas of Benin, and low demand for MDA amongst specific sub-populations driven by religious or traditional beliefs. Due to a six month delay related to COVID-19, this study is currently in the rapid ethnography phase. However, the study will be completed by September 2020.

**Implications for D&I Research:**

Much of the implementation research conducted in sub-Saharan Africa is implemented in a top-down manner, with both implementation determinants and strategies derived in the global North. This project provides an important opportunity to test the potential of participatory action research that involves both community members and Ministry of Health officials to optimize program delivery.

**Primary Funding Source:** DFID

### S-56 Cultural tailoring of a texting intervention to increase uptake of smoking cessation support services in Vietnam

#### Celine Larkin^1^, Thomas Houston^2^, Hoa Nguyen^3^, Stephen Fu^4,5^, Daniel Amante^1^, Cuong Kieu Nguyen^6^, Anh Tuan Vuong^6^, Jessica Wijesundara^1^, Rajani Sadasivam^1^

##### ^1^University of Massachusetts Medical School, Worcester, MA, USA; ^2^Wake Forest University, Winston-Salem, NC, USA; ^3^Baylor Scott &White Health, Dallas, TX, USA; ^4^Minneapolis VA Center for Care Delivery and Outcomes Research, Minneapolis, MN, USA; ^5^University of Minnesota, Minneapolis, MN, USA; ^6^Institute of Population, Health and Development, Hanoi, Viet Nam

###### **Correspondence:** Celine Larkin (celine.larkin@umassmed.edu)

**Background:** Tobacco is a leading preventable cause of mortality, killing more than 8m people each year, mostly in low- and middle-income countries. In Vietnam, 47% of men smoke but only 3% of smokers report receiving cessation counseling. In fact, the “Quitline”, a national telephone-based smoking cessation counseling service established in 2015, is underutilized, receiving only 25 calls daily. In this project, we worked with multiple stakeholders to increase service demand and uptake among rural Vietnamese men and women.

**Methods:** We adapted an evidence-based texting intervention to increase consumer demand for smoking cessation support services in Vietnam, namely a counseling Quitline and nicotine replacement therapy. Before beginning a full scale RCT, we convened former-smoker consultants to review existing motivational text messages and create new messages. First, fourteen Vietnamese-American former smokers reviewed 126 messages derived from experts and smokers in our previous studies using a card-sorting exercise, which was then repeated with five counselors and two doctors from the Quitline service in Vietnam. Finally, former smokers in Vietnam were invited to draft new text messages based on prompts. A bottom-up qualitative coding approach was eventually used to classify the message pool and identify key themes.

**Findings:** Smoker consultants and Quitline counselors identified a total 21 existing text messages that had high acceptability, and these were identified for inclusion in the message pool. 238 new messages were created by Vietnamese former smokers, of which 44 messages were selected for inclusion in the message pool based on independent ranking by three researchers. Messages from both exercises were found to reflect five themes: Quitline as helpful (proactive/encouraging/informative); awareness of smoking’s adverse effects (on self and others); intrinsic motivation (self-efficacy/perseverance/rewards); role of family (in quitting and maintaining cessation); and quitting tips (substitutions/alcohol avoidance/awareness of environmental triggers).

**Implications for D&I Research:** We previously demonstrated that a texting intervention could increase smoking cessation in a U.S. sample. Here, we engaged Vietnamese stakeholders to adapt the messages for cultural appropriateness and meaning, and found divergent preferences and novel themes compared to U.S. smokers. This approach will likely increase consumer-side demand, enhancing the reach of smoking cessation services and increasing cessation in Vietnam.

**Primary Funding Source:** National Institutes of Health

### S-57 Building the capacity of the education system to implement an evidence-based tobacco control intervention for school teachers in India

#### Eve Nagler^1^, Mangesh Pednekar^2^, Dhirendra Sinha^3^, Keyuri Adhikari^2^, Mary Vriniotis^1^, Prakash Gupta^2^, Prof. Glorian Sorensen^1^

##### ^1^Dana-Farber Cancer Institute, Boston, MA, USA; ^2^Healis-Sekhsaria Institute for Public Health, Navi-Mumbai, India; ^3^Healis-Sekhsaria Institute for Public Health, Navi Mumbai, India

###### **Correspondence:** Eve Nagler (eve_nagler@dfci.harvard.edu)

**Background:**

There is a profound need for evidence-based interventions (EBIs) that promote tobacco control on a large scale, particularly in India and other low-and middle-income countries (LMICs) where the tobacco epidemic persists. We build on an intervention for schoolteachers, called the *Tobacco-Free Teachers/Tobacco-Free Society* (TFT-TFS) program, shown to be efficacious in increasing tobacco use cessation and tobacco policy implementation in a cluster-randomized-controlled trial in the Bihar School Teachers’ Study (BSTS). Teachers in India are an important channel for promoting tobacco control, given their roles as community leaders and role models. Guided by the RE-AIM Framework, this study tested if TFT-TFS could be successfully implemented by building the capacity within the Education Department to train and support headmasters to implement TFT-TFS in their schools.

**Methods:**

Six blocks from three Districts in Bihar, India were randomized into either the intervention or control arms. Using a cascade-training model, cluster coordinators in the intervention arm - who routinely interact with headmasters - were trained to train headmasters from their respective clusters to implement TFT-TFS over one academic year. This study used a non-inferiority design to test if program implementation would not be inferior to the high standards demonstrated in BSTS; it also assessed program effectiveness in improving cessation among teachers and policy implementation by comparing schools in the intervention and control arms.

**Findings:**

Despite data collection delays due to COVID-19, preliminary findings indicate 95% of intervention cluster coordinators were trained as TFT-TFS trainers. Nearly 90% of the 218 headmasters *adopted* the program, defined as attending at least one TFT-TFS training. Over 92% of headmasters who adopted the program *implemented* the four core components (group discussions with teachers, hanging thematic posters, providing cessation materials; posting school tobacco policies), with posters being the most implemented component. Program *reach* among teachers in schools that implemented TFT-TFS was 80%. Full results will be available at the conference.

**Implications for D&I Research:**

This study provides a roadmap for D&I research and practice in LMICs to implement tobacco control EBIs within schools using lay interventionists. These findings also demonstrate how this capacity-building approach can be used to scale other public health-related EBIs in resource-constrained areas.

**Primary Funding Source:** National Institutes of Health

### S-58 Crowdsourcing as youth-friendly implementation strategies to increase uptake of HIV testing in Nigeria

#### Ucheoma Nwaozuru^1^, Oliver Ezechi^2^, Titi Gbaja-biamila^2^, Chisom Obiezu-Umeh^1^, David Oladele^2^, Adesola Musa^2^, Ifeoma Idigbe^3^, Florida Uzoaru^1^, Ronda BeLue^1^, Collins Airhihenbuwa^4^, Joseph Tucker^5^, Juliet Iwelunmor^1^

##### ^1^Saint Louis University, Saint Louis, MO, USA; ^2^Nigerian Institute of Medical Research, Yaba, Lagos, Nigeria; ^3^Nigerian Institute of Medical Research, Lagos, Nigeria; ^4^Georgia State University, Atlanta, GA, USA; ^5^University of North Carolina at Chapel Hill, Chapel Hill,, NC, USA

###### **Correspondence:** Ucheoma Nwaozuru (ucheoma.nwaozuru@slu.edu)

**Background:**

Suboptimal HIV testing rates among Nigerian youth creates a pressing need for implementation strategies that not only support youth voice but also increases testing rates, including the uptake of HIV self-testing (HIVST). Crowdsourcing which engages crowds to generate health solutions is a promising implementation strategy to actively engage youth in implementation and adoption of HIV testing services. In this study, we organized a crowdsourcing contest among Nigerian youth to generate strategies to promote HIV self-testing (HIVST) in their communities.

**Methods:**

This contest occurred in three phases. First, Nigerian youths (14-24 years) were invited to share their ideas on how to promote the uptake of HIVST. Second, submissions were evaluated and shortlisted by an expert panel based on desirability (appealing to young people), feasibility (easy to implement), and impact (will significantly influence HIVST uptake among young people). Third, top ideas (finalists) from the evaluation phase were invited to pitch their ideas at a community-facing event. Ideas from the finalist were content analyzed using Powell et al.’s taxonomy of implementation strategies.

**Findings:**

A total of 348 submissions were received (281 online + 67 offline entries). Participants' mean age was 19.6 years (SD=3.4) and 53% were male. We invited 32 shortlisted entrants to pitch their ideas. From the ideas presented,13 unique implementation strategies were identified. These which were categorized as Planning (recruiting and training youth representatives in schools to serve as peer navigators); Education (collaboration with educational institutions to train young people to deliver HIVST kits, training hair salon owners to distribute HIVST kits); Restructure (engaging young people to repackage the HIVST kits to enhance appeal to young people); *Communication* (creating awareness through educational outreach on social media, storytelling; conducting youth-led gain-framed HIV stigma-reduction campaigns), and; *Alternative HIVST provision strategy* (gamification approaches to motive HIVST testing, discrete door-to-door delivery of HIVST) which were not captured by the existing taxonomy.

**Implications for D&I Research:**

Crowdsourcing is a useful and feasible implementation strategy to improve the development and implementation of HIVST strategies among young people in Nigeria. Future research to determine the adoption and effectiveness of crowdsourcing with promoting and increasing uptake of HIV testing are needed.

**Primary Funding Source:** National Institutes of Health

### S-59 Adapting a multi-component hypertension intervention in an under-resourced evolving context in Guatemala: An application of the RE-AIM/PRISM framework with a focus on health equity and sustainability

#### Alejandra Paniagua-Avila^1^, Rachel Shelton^2^, Jiang He^3^, Diego Hernandez-Galdamez^4^, Vilma Irazola^5^, Manuel Ramirez-Zea^6^, Meredith Fort^7^

##### ^1^Columbia University, New York, NY, USA; ^2^Columbia University Mailman School of Public Health, New York, NY, USA; ^3^Tulane University, New Orleans, LA, USA; ^4^INCAP Research Center for the Prevention of Chronic Diseases, Institute of Nutrition of Central America and Panama – INCAP, Guatemala City, Guatemala; ^5^Department of Chronic Diseases, Institute for Clinical Effectiveness and Health Policy (IECS), Buenos Aires, Argentina^6^INCAP Research Center for the Prevention of Chronic Diseases, Guatemala, Guatemala^7^Colorado School of Public Health, Aurora, CO, USA

###### **Correspondence:** Alejandra Paniagua-Avila (mp3856@columbia.edu)

**Background:**

It is urgent to understand how evidence-based interventions are adapted within low-resource settings in response to the COVID-19 pandemic, a striking example of evolving context. In 2019, we began a type-2 effectiveness-implementation trial testing an evidence-based multicomponent hypertension control program within Guatemala’s primary healthcare system. This abstract documents program adaptations occurring during implementation –before and after COVID-19- with a focus on health equity and sustainability.

**Methods:**

We applied the sustainability/health equity extension of the RE-AIM/PRISM framework. In-depth interviews focused on patient-, provider-, and facility-level adaptations to the protocol-based hypertension treatment and implementation strategies: 1. providers training, 2. team-based care, 3. health coaching, 4. blood pressure measurement with audit/feedback, and 5. home-based blood pressure monitoring. Key informants provided informed consent and included providers/administrators (n=15, ongoing), research assistants (n=3), and data collectors (n=5) working in health districts across three states. Through Rapid Identification of Themes from Audio Recordings (RITA) and matrix-based thematic analysis we captured actionable findings addressing sustainability and health equity issues. Analysis will finalize by September 2020.

**Findings:**

Preliminary results show that all program components have undergone content / delivery adaptations. Before COVID-19, all districts reduced the frequency/duration of health coaching sessions and team meetings, while expanding the content of providers training. After COVID-19, team meetings, audit/feedback and providers training stopped; delivery mode of health coaching sessions and hypertensive shifted; and frequency of home-based BP increased. Adaptations leading to patterns of health inequities include delivering phone-base shortened health coaching sessions, as they do not reach those without phone. Delivering medications at patients’ homes, rather than the health post, might facilitate equitable access to medications. Contextual factors threatening sustainability include increased staff overload and reduced financial resources due to COVID-19. Throughout the implementation, key factors leading to adaptations that are likely to enhance program sustainability include: a district-level program champion, a previously established hypertension program and community support.

**Implications for D&I Research:**

The sustainability / health equity extension of the RE-AIM/PRISM framework has enabled us to capture adaptations of an evidence-based hypertension program being implemented and tested in Guatemala’s primary healthcare system in response to the COVID-19 pandemic.

**Primary Funding Source:** National Institutes of Health

### S-60 Project khanya: Evaluating the implementation of a peer-delivered behavioral intervention for substance use and HIV medication adherence in a low-resource, global HIV care setting

#### Jessica Magidson^1^, Jennifer Belus^2^, Lena Andersen^3^, Sybil Majokweni^3^, Alexandra Rose^2^, Kristen Regenauer^2^, Nonceba Ciya^3^, Morgan Anvari^2^, Bronwyn Myers^4^, John Joska^3^, Steven Safren^5^

##### ^1^University of Maryland College Park, College Park, MD, USA; ^2^University of Maryland, College Park, MD, USA; ^3^University of Cape Town, Cape Town, South Africa; ^4^South African Medical Research Council, Parow, South Africa; ^5^University of Miami, Miami, FL, USA

###### **Correspondence:** Jessica Magidson (jmagidso@umd.edu)

**Background:** Substance use is prevalent in South Africa and associated with poor HIV outcomes, yet, it is largely unaddressed in HIV care. Implementation questions remain regarding how to integrate substance use treatment into HIV care. We evaluated the implementation of an evidence-based, task-shared intervention for substance use and antiretroviral therapy (ART) adherence in HIV care in South Africa.

**Methods:** Guided by the RE-AIM framework, a randomized, hybrid type 1 effectiveness-implementation trial (*n*=61) was conducted to evaluate “Khanya,” a six-session, peer-delivered intervention that integrates problem solving and motivational skills for ART adherence (Life-Steps), behavioral activation to increase substance-free rewarding activities, and relapse prevention skills (including mindfulness), with an optional six booster sessions. Individuals were randomized to receive Khanya or enhanced standard of care (ESOC), a facilitated referral to an on-site substance use treatment clinic—Matrix, an evidence-based substance use treatment model. Implementation outcomes were defined by Proctor’s model and included mixed methods evaluations of feasibility, acceptability, appropriateness, and fidelity, using semi-structured individual interviews and the Applied Mental Health Research (AMHR) implementation science measure (items rated on a 0-3 scale). An independent rater coded a random subsample (20%) of intervention sessions for content fidelity and peer clinical competence using select items from the ENhancing Assessment of Common Therapeutic factors (ENACT) scale (items rated on a 1-3 scale).

**Findings:** Individuals randomized to Khanya attended on average 4.96 (*SD*=1.63) sessions (of 6 total); 80.6% (*n*=25) in the control condition were linked to Matrix. Feasibility (*M*=2.92; *SD*=0.18), acceptability (*M*=2.99; *SD*=0.04) and appropriateness (*M*=2.94; *SD*=0.09) were rated highly for the intervention condition. For content fidelity, the interventionist delivered 92% (*SD*=13%) of components as intended. Establishing peer role fidelity, the mean score for ENACT items was *M*=2.69 (*SD*=0.28). Qualitative data indicated the acceptability of receiving the intervention from a peer, feasibility of implementing mindfulness in daily life, and interest in expanding the intervention to community and/or group formats.

**Implications for D&I Research:** Results of this trial provide important evidence regarding the feasibility, acceptability, appropriateness, and fidelity of a peer-delivered integrated intervention for ART adherence and substance use in a resource-limited, global HIV care setting and will inform a subsequent larger trial.

**Primary Funding Source:** National Institutes of Health

### S-61 Optimizing treatment cascades for mental healthcare in Mozambique: Preliminary effectiveness of the systems analysis and improvement approach for mental health (SAIA-MH)

#### Katrin Fabian^1^, Alberto Muanido^2^, Vasco Cumbe^3^, Nelia Manaca^2^, Leecreesha Hicks^4^, Dr. Bryan Weiner^5^, Kenny Sherr^6^, Bradley Wagenaar^1^

##### ^1^University of Washington, Seattle, WA, USA; ^2^Health Alliance International, Beira, Mozambique; ^3^Department of Mental Health, Ministry of Health, Beira, Mozambique^4^Health Alliance International, Seattle, WA, USA; ^5^University of Washington School of Public Health, Seattle, WA, USA; ^6^Global Health, University of Washington, Seattle, WA, USA

###### **Correspondence:** Bradley Wagenaar (bwagen@uw.edu)

**Background:** Substantial investments are being made to scale-up access to mental healthcare in low-and middle-income countries (LMICs), but less attention has been paid to quality and performance of nascent public-sector mental healthcare systems. The present study tested the initial effectiveness of an implementation strategy to optimize routine outpatient mental health care cascade performance in Mozambique (the Systems Analysis and Improvement Approach for Mental Health [SAIA-MH]).

**Methods:** This study employed a pre-post design from September 2018 to August 2019 across four Ministry of Health clinics among 810 patients and 3,234 outpatient mental health visits. Effectiveness outcomes evaluated progression through the care cascade, including: (1) initial diagnosis and medication selection; (2) enrolling in follow-up care; (3) returning after initial consultation within 60 days; (4) returning for follow-up visits on time; (5) returning for follow-up visits adherent to medication; and (6) achieving function improvement. Clustered generalized linear models evaluated odds of completing cascade steps pre- versus post-intervention.

**Findings:** Facilities prioritized improvements focused on the follow-up cascade, with 62.5% (10 of 16) monthly system modifications targeting medication adherence. At baseline, only 1 in 23.8 (4.2%) patient visits achieved function improvement; during the 6 months of SAIA-MH implementation, this improved to 1 in 7.6 (13.1%) patient visits. Multilevel logistic regression found increased odds of returning on time and adherent (aOR = 1.53, 95% CI [1.21, 1.94], p = 0.0004) and returning on time, adherent, and with function improvement (aOR = 3.68, 95% CI [2.57, 5.44], p <0.0001) after SAIA-MH implementation. No significant differences were observed regarding other cascade steps.

**Implications for D&I Research:** The SAIA-MH implementation strategy shows promise for rapidly and significantly improving mental healthcare cascade outcomes, including the ultimate goal of patient function improvement. Given poor baseline mental health care cascade performance, an urgent need exists evidence-based implementation strategies to optimize the performance of mental health care cascades in LMICs.

**Primary Funding Source:** National Institutes of Health

### S-62 Qualitative investigation of determinants for implementation and sustainability of adolescent mental health services integrated within mozambican primary care settings

#### Kate Lovero^1^, Bianca Kann^1^, Salma Adam^2^, Carolina Bila^2^, Maria Eduarda Fernandes^2^, Teresa Rodrigues^2^, Rinad Beidas^3^, Milton Wainberg^4^

##### ^1^Columbia University Medical Center/New York State Psychiatric Institute, New York, NY, USA; ^2^Mozambique Ministry of Health, Maputo, Mozambique; ^3^University of Pennsylvania Perelman School of Medicine, Philadelphia, PA, USA; ^4^Columbia University/New York State Psychiatric Institute, New York, NY, USA

###### **Correspondence:** Kate Lovero (kate.lovero@nyspi.columbia.edu)

**Background**: Mental disorders are the primary cause of disability in adolescents worldwide, accounting for 45% of years lived with disability.^1^ It is estimated that 90% of adolescents live in low- and middle-income countries (LMIC),^2^ and that 10-20% of these adolescents have a mental disorder.^3^ Despite the substantial burden of mental illness, adolescent mental health services are extremely limited in LMIC.^4^ Mental health services delivered by non-specialists (task-shifting) within primary care of LMIC has been proposed as an efficient way to close the adolescent mental health treatment gap. However, research is needed to determine how to effectively integrate and sustain EBP for managing adolescent mental disorders within LMIC primary care settings.^5,6^

**Methods**: We conducted a qualitative exploration of adolescent, caregiver, health provider, and policymaker perspectives on implementing and sustaining group-based mental health interventions within primary care settings, guided by the Consolidated Framework for Implementation Research (CFIR)^6^ and Integrated Sustainability Framework.^7^ Trained local researchers conducted key informant interviews with Ministry of Health officials involved in adolescent (n=4) and mental health (n=4) programming as well as focus groups with adolescents (n=8) and caregivers (n=5). Following COVID-limitations on in-person research, in lieu of focus groups, we conducted tele-interviews with providers of adolescent (n=4) and mental (n=4) healthcare. Qualitative transcripts were analyzed using the best fit framework approach.^8^

**Findings**: Results indicated great interest from all types of participants in integration of group mental health interventions in primary care. Participants indicated mental health services provided by non-specialists were acceptable and often preferable. Primary determinants of adoption were identified in the intervention characteristics, specifically complexity and adaptability to the local context, as well as implementation process, specifically promoting early engagement and buy-in of providers and clinic administration. Primary determinants of sustainability were identified in the implementation process, including assuring continued supervision/quality of service provision and feedback for providers and clinic administration on intervention outcomes.

**Implications for D&I Research:** Results from this study will inform development of the implementation strategy for delivering adolescent mental health services in Mozambican primary care. Broadly, this work adds to the very limited knowledge of key determinants for implementation and sustainability of mental health services for adolescents in LMIC.

**Primary Funding Source:** National Institutes of Health

### S-63 Results of a cluster randomized trial testing a systems analysis and improvement approach (SAIA) versus usual procedures to increase HIV counseling and testing in family planning clinics in mombasa, Kenya

#### McKenna Eastment^1^, George Wanje^2^, Barbra Richardson^3^, Emily Mwaringa^4^, Kenneth Sherr^1^, Ruanne Barnabas^1^, M. E. Perla^1^, Kishorchandra Mandaliya^1^, Walter Jaoko^5^, R. Scott McClelland^1^

##### ^1^University of Washington, Seattle, WA, USA; ^2^Ganjoni Clinic, Mombasa, Kenya; ^3^Fred Hutchinson Cancer Research Center, Vaccine and Infectious Disease Division, Seattle, WA, USA; ^4^Mombasa County Department of Health, Mombasa, Kenya; ^5^University of Nairobi, Nairobi, Kenya

###### **Correspondence:** McKenna Eastment (mceast@uw.edu)

**Background:** In high HIV prevalence settings, integration of HIV counseling and testing (HCT) into family planning (FP) clinics holds promise for improving women’s health and reducing HIV transmission. This study tested an implementation strategy, the Systems Analysis and Improvement Approach (SAIA), to increase rates of HCT in FP clinics in Mombasa, Kenya.

**Methods:** Twenty-four FP clinics were randomized 1:1 to an intervention arm implementing SAIA versus a control arm with no intervention. Study staff implemented monthly SAIA cycles for 12 months. SAIA has five steps. Step 1 uses a “cascade analysis” tool to quantify the number of individuals who complete each step of a process. Step 2 involves sequential process flow mapping to identify modifiable bottlenecks in the system. Step 3 develops and implements a workflow modification to address bottlenecks. Step 4 assesses impact of the modification by recalculating the cascade analysis to determine whether performance has improved. Step 5 repeats the cycle. The primary outcome was the proportion of new FP clients tested for HIV during the last three months of the trial.

**Findings:** During the last three months of the trial, 42% (364/859) of FP clients were tested for HIV at intervention clinics compared to 32% (485/1521) at control clinics (prevalence rate ratio [PRR] 1.33, 95% confidence interval [CI] 1.16-1.52). The intervention effect was different in public versus private clinics (interaction term p-value 0.001). SAIA led to more HIV testing in public intervention versus control clinics (PRR 1.59, 95%CI 1.35-1.89), but not in private intervention versus control clinics (PRR 0.97 95%CI 0.77-1.23). Eighty-five percent (740/868) of new FP clients were counseled for HIV testing in intervention clinics compared to 67% (1036/1542) in control clinics (PRR 1.27, 95%CI 1.15-1.30). SAIA’s effect on HIV counseling was similar in public and private clinics.

**Implications for D&I Research:** SAIA led to a significant increase in HIV testing in FP clinics in Mombasa County. The next steps are to scale-up SAIA while transitioning implementation from research staff to County Ministry of Health staff to understand its scalability and sustainability.

**Primary Funding Source:** National Institutes of Health

### S-64 Measuring the m in the RE-AIM framework: Using a stepped wedge design to evaluate maintenance of the saia-scale PMTCT program post-external support in Mozambique

#### Jonny Crocker^1^, Mery Agostinho^2^, Fernando Amaral^2^, Kristjana Asbjornsdottir^1^, Joana Coutinho^3^, Emilia Cruz^4^, Aneth Dinis^1^, Sarah Gimbel^1^, Celso Inguane^1^, Kenneth Sherr^1^

##### ^1^University of Washington, Seattle, WA, USA; ^2^Health Alliance International, Mozambique, Chimoio, Mozambique; ^3^Health Alliance International, Beira, Mozambique; ^4^Health Alliance International, Beira, WA, Mozambique

###### **Correspondence:** Jonny Crocker (crockerj@uw.edu)

**Background:** There is lack of models and approaches for evaluating the maintenance and sustainment of healthcare implementation strategies and programs once external support is reduced or removed. Thus, there is limited evidence on maintenance of these same programs – which indicates that promising programs often fail to be maintained and produce anticipated benefits. In many cases, these programs specifically involve training of health providers in an effort to build capacity in the public sector to maintain these programs with limited external support.

**Methods:** We use a stepped wedge design to incorporate prospective evaluation of program maintenance into the study period. The SAIA-Scale stepped wedge trial includes three 12-month intensive implementation waves, each comprising 12 health facilities. During intensive implementation, study nurses train district officials to use the Systems Analysis and Improvement Approach, a 5-step systems engineering approach, to improve prevention of mother to child transmission of HIV (PMTCT). Study nurses then accompany district officials on supervision visits to selected health facilities to implement the SAIA approach. A maintenance phase follows, in which districts receive solely to minimal financial support. District officials performed implementation tracking by self-report their supervision visits using a mobile survey tool. The primary metrics of program maintenance is frequency of supervision visits to health facilities post intensive implementation.

**Findings:** During wave 1 of the intensive implementation phase (April 2018–March 2019), supervision visits to health facilities occurred an average 1.14 times/month. During the maintenance phase (April 2019–June 2020), supervision visits occurred an average of 0.99 times/month, corresponding to a 12.7% reduction in frequency. The average number of health facility staff participating in supervision visits was 7.8 during the intensive phase, and 8.4 during the maintenance phase, corresponding to a 7.7% increase.

**Implications for D&I Research:** The stepped wedge design combined with implementation tracking allowed for prospective evaluation of program maintenance after external support decreased. A modest reduction in supervision frequency and increase in participation indicate the program was well maintained.

**Primary Funding Source:** National Institutes of Health

## Health Policy Dissemination and Implementation Science

### S-65 Dismantling of state-level food environment policies

#### Alexandra Morshed

##### Washington University in St. Louis, St. Louis, MO, USA

###### **Correspondence:** Alexandra Morshed (a.b.morshed@wustl.edu)

**Background:** Growing research exists on obesity policy knowledge translation and enactment. However, little is known about the phenomenon of dismantling of obesity-related policies, i.e., decrease, diminution, or elimination of existing public policies. We focused on policies related to food environments (e.g., food/beverage taxes) which have a large role in shaping obesity-related dietary behaviors. The purpose of this study was to quantify and identify determinants of state-level dismantling of food environment policies.

**Methods:** Fifty-one policies in 32 states in place or enacted between 2009 and 2018 were systematically identified from publicly available policy tracking and surveillance sources. Policies were retrospectively followed during 2009-2018 and coded for attempted and successful legislative dismantling attempts. Potential predictors and covariates of policy dismantling were measured at the policy and state levels, including policy procedure and composition and state political, vested interest, sociodemographic, and economic factors. Proportion of policies that faced dismantling, five-year survival rates, and survival probability curves were calculated by dismantling category. Multilevel Poisson regression and survival analysis adjusted for clustering within states were used to identify predictors of dismantling.

**Findings:** Over a period of five-years, 28% of policies can expect to face any dismantling attempts and 11% are successfully dismantled. Levying taxes and greater number of years under a Republican governor were positively associated with the risk of dismantling, while preemption policies and governor party turnover were negatively associated with risk of dismantling. In predicting dismantling incidence, levying taxes and Senate origin of a policy were associated with greater incidence of dismantling, while number of policy sponsors and number of years Republicans controlled the legislature were protective.

**Implications for D&I Research:** This study fills an important research gap by characterizing to what extent the phenomenon of dismantling is happening at the state level for food environment policies. It also fills a gap in D&I literature by characterizing de-implementation at the policy level, including initial findings about what modifiable determinants exist to inform potential strategies. Public health professionals and advocates should be prepared to respond to dismantling by identifying strategies for ensuring that evidence-based policies remain in place and harmful or ineffective ones are removed.

**Primary Funding Source:** National Association of Social Workers Foundation Jane B. Aron / Social Work HEALS Fellowship

### S-66 Implications for policy implementation measurement from a systematic review

#### Peg Allen^1^, **Callie Walsh-Bailey**^2^, Jonathan Purtle^3^, Meagan Pilar^4^, Cole Hooley^5^, Ross Brownson^1^

##### ^1^Brown School, Prevention Research Center, Washington University in St. Louis, St. Louis, MO, USA; ^2^Washington University in St. Louis, St. Louis, MO, USA; ^3^Health Management &Policy, Drexel University School of Public Health, Philadelphia, PA, USA; ^4^Washington University in St. Louis, St Louis, MO, USA; ^5^Brigham Young University, Provo, UT, USA

###### **Correspondence:** Callie Walsh-Bailey (callie.w@wustl.edu)

**Background:** While policy development has been extensively studied, less is understood about which policy implementation processes are key to successful implementation in clinical and public health practice. There has been relatively little D&I research to inform policy implementation strategies and evaluate implementation efforts. The purpose of the systematic review was to identify quantitative measures of policy implementation outcomes and determinants that can be applied across health policy topics and settings.

**Methods:** We searched six databases for empiric quantitative or mixed methods studies of policy implementation in any policy topic or setting pertaining to physical, mental, or behavioral health. Studies were included if published in English in peer-reviewed journals from January 1995 to April 2019 and used quantitative measures to assess Implementation Outcome Framework outcomes or implementation determinants from other frameworks. We conducted dual independent abstract screening and full text screening, followed by consensus abstraction by two coauthors.

**Findings:** The study team provided a compendium of identified measures in a publicly available website launched in 2020. Few measures of external contexts affecting health policy implementation were identified in the 70 extracted measures: actor relationships and networks (17%), political will for implementation (10%), and little else. Measures that assessed readiness for implementation (61%) or intra-organizational climate and culture (39%) were more commonly identified than measures of implementation outcomes such as fidelity (26%) or acceptability (24%). Measures overall scored well on pragmatic quality ratings (e.g., free or inexpensive to access, suitable reading level). Few studies documented pilot or psychometric testing of instruments.

**Implications for D&I Research:** Refinement of implementation outcome definitions and delineation of additional policy implementation outcomes (such as enforcement) can further policy D&I research. More extensive development and testing of measures of external policy implementation contexts are needed to inform and evaluate policy implementation. Reporting pilot testing and measure development, however briefly with links to supplemental materials, will help other researchers. Identification and sharing of valid, reliable, and pragmatic policy implementation measures specific to health policy topics and settings will inform future policy implementation beyond this initial broad brush review.

**Primary Funding Source:** National Institutes of Health

### S-67 Future directions in policy implementation science

#### Ali Abazeed

##### National Institutes of Health, National Cancer Institute Bethesda, MD, USA

###### **Correspondence:** Ali Abazeed (ali.abazeed@nih.gov)

**Background:** Implementation Science (IS) has developed as the result of strategic training activities, an emphasis on the development of conceptual frameworks and measures, increased acceptance of appropriate study designs, and a dedicated review mechanism. We continue to explore how implementation science may contribute to understanding the translation of scientific evidence to health-related policy in governmental and non-governmental sectors—an area of inquiry that we refer to as *policy implementation science (policy IS)*. The considerable body of implementation research underway rarely utilizes policy approaches and we believe there are several key opportunities that could build the field’s strength in policy IS.

**Methods:** As part of a planning process sponsored by the Implementation Science team at NCI’s Division of Cancer Control & Population Sciences (DCCPS), we conducted 31 interviews with stakeholders and NCI and other federal employees working at the intersection of policy and health research. Stakeholders spanned geographic zones across the US with expertise in national and global health policy who work in diverse disciplines including political science and public policy, public health law, behavioral science, and social policy among others. Stakeholders from outside the federal government offered recommendations for areas of research focus and identified barriers and facilitators to conducting more policy-focused implementation research. Stakeholders from within the government identified existing structures that can be utilized to support policy implementation research, as well as some of the challenges.

**Findings:** Our conversations yielded six domains through which we intend to increase policy IS engagement: field-seeding/writing, training programs, grant reviews, data centralization, research agenda development, and stakeholder engagement. We are arranging our work by those that we consider to be tenable within months, those that have significant value-add, but are likely take more time and resources, and those that are worth considering as part of a long-term strategy.

**Implications for D&I Research:** Taken as a whole, these interviews highlighted the important role that policy implementation science can play in the improvement of population health. This presentation will provide an overview of the information generated in the key informant interviews, as well as several steps that the NCI Implementation Science Team will be taking to support policy implementation science.

### S-68 A document analysis methodology for tracking and assessing use of research evidence in health policymaking

#### Itzhak Yanovitzky^1^, Matthew Weber^2^

##### ^1^Communication, Rutgers University, New Brunswick, NJ, USA; ^2^Hubbard School of Journalism and Mass Communication, University of Minnesota, Minneapolis, MN, USA

###### **Correspondence:** Itzhak Yanovitzky (itzhak@rutgers.edu)

**Background:** Document analysis can be a useful and robust methodology for tracking and assessing use of research evidence (URE) in health policymaking, if applied correctly. Relative to other methods, document analysis affords researchers a flexible, unobtrusive way of tracking URE longitudinally (both prospectively and retrospectively) while situating measurement in the unique context of the underlying policymaking process and the actors involved. Both quantitative and thematic analyses of the data are possible, producing rich insights, as well as augment other methods and advanced applications (e.g., network analysis of evidence networks).

**Methods:** The goal of rigorous document analysis is to represent the different routes through which policymakers obtain research; the scope and nature of the research evidence acquired; how research evidence is interpreted; and how research is used (e.g., instrumental, conceptual, or political use).This requires a careful selection and sampling of documents the capture URE in the context of the policy decisionmaking process; a theory-grounded measurement of use; valid and reliable coding of documents; and data analysis that centers on exposing patterns of use (research use routines) and conditions that facilitate or impede use.

**Findings:** The presentation is designed to walk participants through the key methodological decisions involved in ensuring a rigorous application of document analysis to track and assess URE in the context of health policymaking. An illustrative example from a project that uses document analysis in combination with other methods to track state mental health policy regarding the prevention of adolescent depression and suicide is provided, as well as additional useful resources.

**Implications for D&I Research:** Document analysis has significant potential to advance the standardization of URE measurement in the context of health policymaking, whether as a stand-alone method or in combination with other methods. However, it also has a number of important limitations and a significant potential to bias findings if not employed rigorously. The goal if this presentation is to introduce a robust approach to document analysis that capitalizes on its significant affordances and minimize potential limitations and bias.

**Primary Funding Source:** William T. Grant Foundation

### S-69 Analyzing survey data from policymakers in d&I research: New approaches

#### Jonathan Purtle

##### Health Management &Policy, Drexel University School of Public Health, Philadelphia, PA, USA

###### **Correspondence:** Jonathan Purtle (jpp46@drexel.edu)

**Background:** Public policymakers are an extremely heterogenous audience who make decisions in highly complex contexts. Policymakers are increasingly surveyed in D&I research, but traditional analytic approaches are not always optimally suited capture such heterogeneity and complexity. This presentation will illustrate how three analytic approaches—*agent-based modeling (ABM)*, *latent class analysis (LCA)*, and *small area estimation*—have been applied to analyze survey data from policymakers in D&I research.

**Methods:** *ABM* is a computational technique that simulates the actions of individuals within a hypothetical context. A 2019 survey of 224 state mental health agency policymakers was conducted about uses of research evidence in decision-making about children’s mental health. These data informed an ABM that simulated the frequency of research use under different scenarios of policymaker-researcher collaboration. *LCA* is technique that explores relationships between multiple variables to identify single latent variables. A survey of 475 state legislators was conducted about their opinions related to behavioral health issues. These data were analyzed using LCA to identify “audience segments” of legislators who vary in their opinions about behavioral health. *Small area estimation* is a technique that approximates the health status of a population within a highly specific geopolitical jurisdiction. A 2016 survey of 224 city mayors was conducted about their beliefs related to health disparities in their cities. Small area estimation was used to generate estimates of the magnitude of health disparities in these cities and multi-level regression assessed associations between the magnitude of disparities and mayors’ opinions about disparities.

**Findings:** The ABM elucidated how strengthening policymaker-researcher collaborations could increase the use of research evidence in children’s mental health policymaking, highlighting promising areas for intervention. The LCA identified three discrete segments of legislators, shedding light on how dissemination strategies could be tailored for each segment. The small area estimation analysis found that the magnitude of health disparity in a mayor’s city was independently associated with the mayor’s beliefs about disparities, illuminating how population health status could function as an outer-contextual factor in policy-making and implementation.

**Implications for D&I Research:** Findings from these analytic approaches can inform the selection and design of policymaker-focused dissemination and implementation strategies.

**Primary Funding Source:** The Robert Wood Johnson Foundation

### S-70 Tools for measuring federal lawmakers’ use of research evidence: Legislative surveys and bill coding

#### Elizabeth Long^1^, Lawrie Green^2^, Rebecca Smith^3^, Lauren Supplee^4^, Elizabeth Jordan^5^, Jennifer Taylor Scott^1^, Max Crowley^6^

##### ^1^Penn State University, University Park, PA, USA; ^2^Pennsylvania State University, University Park, PA, USA; ^3^Virginia Commonwealth University, Richmond, VA, USA; ^4^Early Childhood Research, Child Trends, Bethesda, MD, USA; ^5^Child Trends, Bethesda, MD, USA; ^6^Pennsylvania State University, State College, PA, USA

###### **Correspondence:** Elizabeth Long (ecl5218@psu.edu)

**Background:** Despite growing recognition of the need to use research evidence to inform policy making, the development and validation of tools to measure the use of research evidence (URE) are emergent and methodologies can be highly context-dependent. For instance, quantitative surveys have mostly been tested in regional or administrative contexts rather than the within the US federal legislature. Qualitative coding of legislative bills may also vary based on purpose area. Accordingly, robust methods to assess URE in varied contexts are needed. The focus of the current study is the US Congress.

**Methods:** A newly developed survey, the Legislative Use of Research Survey (LURS), was administered to 80 congressional staff to measure their reported research use, value of research, interactions with researchers, general information sources, and research information sources. Descriptive statistics and structural validity were explored by conducting confirmatory factor analyses (CFAs) on each of these 5 scales. We then trimmed the number of items based on poor factor loadings and ran the CFAs again. This trimmed measure was administered to staff again after an intervention designed to improve URE, the Research-to-Policy Collaboration, was implemented. To complement the survey data, we developed a qualitative coding framework to evaluate the presence and purpose of URE in legislation. To date, we have coded 127 bills related to child and family well-being.

**Findings:** Trends in baseline descriptive statistics will be presented. The fit of our CFA models for each scale were substantially improved in the trimmed measure, all of our items showed acceptable factor loadings, and each scale demonstrated acceptable to strong reliability. Staff receiving the intervention reported an increase in the value of URE by 7%. We will also discuss the different ways that research was used in legislative bills and how it changed across time.

**Implications for D&I Research:** This work offers two novel tools for assessing URE in health policymaking at the federal level. We have demonstrated that it is not only possible to rigorously study federal policymakers’ URE, but also to effect and measure change in how much they value it.

**Primary Funding Source:** William T. Grant Foundation

### S-71 Policy sustainability research to advance health equity: A mixed-methods case study of the implementation and sustainability of tobacco control policies in New York City

#### **Mr. Matthew Lee**^1,2^, Simona Kwon^3^, Rienna Russo^2^, Jonathan Purtle^4^, Rachel Shelton^1^

##### ^1^Columbia University Mailman School of Public Health, New York, NY, USA; ^2^NYU School of Medicine, New York, NY, USA; ^3^Population Health, NYU School of Medicine, New York, NY, USA; ^4^Health Management &Policy, Drexel University School of Public Health, Philadelphia, PA, USA

###### **Correspondence:** Matthew Lee (ml3440@cumc.columbia.edu)

**Background:** Despite evidence of successful implementation and scale-up of effective tobacco control policies in New York City (NYC) and nationally, recent research suggests striking smoking-related health inequities have persisted across communities, particularly among Asian Americans. Sustainability research in implementation science has largely focused on evidence-based programs and guidelines, with very little work focused on the sustainability of evidence-based policies. This study addresses this gap by examining the extent to which NYC tobacco policies have been adapted and sustained, and how these factors impact health equity.

**Methods:** A concurrent mixed-methods data collection design was used across five key sources: 1) policymaking documents (text/video/audio from when key tobacco bills and statutes were first proposed, debated, and adopted); 2) media documents (local newspaper coverage of the policies and their impacts on communities over time); 3) key informant interviews (n=21) with leaders at local agencies and community-based organizations that serve Asian American populations; 4) direct observation periods (n=14) in Asian American neighborhoods/ethnic enclaves; and 5) secondary analysis of the Community Health Survey conducted annually by the NYC Department of Health and Mental Hygiene.

**Findings:** Key findings include: 1) Asian Americans in NYC are typically not considered in policymaking or policy implementation until after a policy has been adopted and maintained; 2) delayed policy adaptation and resource allocation to reach Asian American communities may not be sufficient for existing policies to either achieve their intended health outcomes or reduce health inequities; 3) in response to inconsistent policymaker attention and engagement, Asian American-serving health and advocacy organizations have recently altered their strategies to build political power; and 4) NYC tobacco control policies have experienced both program drift and voltage drop during the sustainment phase for Asian Americans and other underserved populations.

Implications for D&I Research: This work extends on prior findings that uneven, “one-size-fits-all” approaches to policy implementation may contribute to limited reach and inequitable impacts among minoritized and marginalized communities, to focus explicitly on sustainability processes and outcomes, and advancing health equity. We present key dimensions that influence the equitable sustainability of evidence-based policies, as well as an updated operational definition of “policy sustainability” to inform future efforts in this understudied area.

**Primary Funding Source:** The Robert Wood Johnson Foundation

## Models, Measures, and Methods

### S-72 Assess: A comprehensive tool to support reporting and critical appraisal of qualitative, quantitative, and/or mixed methods implementation research outcomes

#### **Nessa Ryan**^1^, Dorice Vieira^2^, Joyce Gyamfi^1^, Temitope Ojo^1^, Emmanuel Peprah^1^

##### ^1^New York University School of Global Public Health, New York, NY, USA; ^2^New York University Langone Health, New York, NY, USA

###### **Correspondence:** Nessa Ryan (ryann01@nyu.edu)

**Background:**

Helpful tools exist to improve reporting of implementation studies and to guide critical appraisal of interventional studies; however, researchers, practitioners, and policy makers have no tool to support the concurrent synthesis and critical assessment of outcomes for intervention and implementation research. Our objective: develop a comprehensive tool to 1) describe studies focused on implementation that use qualitative, quantitative, and/or mixed methodologies, and 2) assess risk of bias of implementation and intervention outcomes

**Methods:**

We modeled our approach after recommended methodology for developing health research reporting guidelines and critical appraisal tools, including literature review, iterative rounds of tool integration and modification through consensus, application of the tool to various study types*,* and invitation of expert feedback on draft versions. We combined elements of the StaRI checklist (Pinnock et al., 2017), MMAT tool (Hong et al., 2018), implementation and health service outcomes taxonomy (Proctor et al., 2011), FAME (JBI, 2014), TIDieR checklist (Hoffman et al, 2014), and FRAME (Stirman et al., 2019) to produce ASSESS.

**Findings:**

The 24 items are applicable to a broad range of study designs employed in implementation science, including qualitative, RCTs, non-randomized, and mixed methods studies. A key concept is the dual columns for implementation strategy and intervention, within which the methods and results are described and the intervention and implementation outcomes are assessed for bias. We have prepared an accompanying explanation and elaboration document that identifies and describes each of the items, explains the rationale, and models examples of good reporting and appraising practice, as well as an instructional video.

**Implications for D&I Research:**

The comprehensive, adaptable ASSESS tool addresses the challenge of critical assessment of a methodologically diverse and growing body of implementation science literature. This tool could prove particularly helpful for designing and carrying out reviews and meta-analyses of empirical studies of implementation (i.e., primary research based on experiment, observation, or simulation and focused in part or fully on implementation), examining how process and context may lead to heterogeneity of results. Its use could improve the reporting and synthesis of implementation strategies, which will facilitate translation of effective public health interventions into routine practice within clinical or community settings.

### S-73 Developing a set of standard implementation outcome measures for HIV interventions to connect research projects and create generalizable knowledge from diverse settings

#### Dennis Li^1,2^, Nanette Benbow^1^, J.D. Smith^1,3^, Brennan Keiser^2^, Juan Villamar^1^, Brian Mustanski^1,2^

##### ^1^Northwestern University Feinberg School of Medicine, Chicago, IL, USA; ^2^Northwestern University, Chicago, IL, USA; ^3^University of Utah School of Medicine, Salt Lake City, UT, USA

###### **Correspondence:** Dennis Li (dennis@northwestern.edu)

**Background:**

The HIV field has moved rapidly towards implementation research (IR) in the last decade. In 2019, the Ending the HIV Epidemic (EHE) initiative funded 65 planning projects across the US focusing on IR. However, shifting from individual/patient-level to systems-level outcomes presented a challenge for some researchers. Although established implementation evaluation frameworks exist, guidance is needed on how to operationalize constructs, especially when IR begins to proliferate in a new health domain.

**Methods:**

Employing the RE-AIM and Proctor frameworks, we operationalized implementation outcomes for 8 categories of HIV interventions investigated by EHE projects (e.g., pre-exposure prophylaxis, molecular surveillance). We held 6 informal discussions with groups of projects and 2 meetings with representatives from CDC and HRSA to assess utility of the resulting crosswalk. Last, we used a modified Delphi method with 11 HIV IR experts to discuss the crosswalk and rate each outcome based on importance to different stages of IR (i.e., pre-implementation, testing/trialing, going to scale).

**Findings:**

We found sufficient similarity across HIV interventions to warrant a standard set of metrics, with added considerations for specific interventions. The crosswalk was organized by outcome domain, level (i.e., site, implementer, patient), and research question. Of 71 proposed measures, 9 fell under Reach; 13 under Effectiveness; 30, Adoption; 10, Implementation; and 9, Maintenance. Table 1 shows importance by IR stage. Across stages, 13 patient-level constructs were rated “if relevant to research” (e.g., hybrid effectiveness–implementation designs).

**Implications for D&I Research:**

Coordinating outcomes for HIV IR nationally facilitates synthesis and creation of generalizable knowledge from diverse local settings. Our crosswalk expands upon common frameworks to explicitly detail what constructs should be used at which IR stage and their measurement for HIV interventions. This process highlighted the stability of some outcomes compared to changing importance of others across stages. The importance of qualitative methods was also emphasized. The crosswalk may be readily adaptable for other disease areas. Next steps include refining the supplemental language to aid in dissemination and use.

**Primary Funding Source:** National Institutes of Health

**Table 1 (abstract S73).** Number of measures by importance and IR stage

Rating	Pre-Implementation	Pilot	Scale-Out
Required	6	23	22
Recommended	10	23	28
If desired	0	12	8
N/A	42	0	0

### S-74 The implementation strategies and satisfaction survey: Evaluating implementation strategy use for an early intensive behavioral intervention (EIBI) among community agencies serving children with autism

#### Tatiana Bustos, Aksheya Sridhar, Andrew Guhin, Amy Drahota

##### Michigan State University, East Lansing, MI, USA

###### **Correspondence:** Tatiana Bustos (bustosta@msu.edu)

**Background:** Despite recent efforts to standardize D&I terminology, taxonomy and research reporting^1,2,3^, documenting discrete implementation strategies being utilized in community-based settings remains uncommon. This study identified and evaluated strategies utilized to implement an early intensive behavioral intervention (EIBI) for children with autism among university and community-based sites in order to support translational efforts into real-world settings.

**Methods:** Using a sequential explanatory mixed methods design, survey and interview data were collected from directors, supervisors and direct providers (*N* = 26) across three EIBI sites. The Implementation Strategies and Satisfaction Survey (ISSS),^4^ derived from literature identifying and defining a taxonomy of implementation strategies^1,3^, was administered to identify staff-reported implementation strategy use and satisfaction (6-point Likert scale). Informed by quantitative results, follow-up semi-structured interviews were conducted with a subsample (*n* = 13) to further explore staff experiences with the implementation strategy used and gather suggestions for improving future efforts.

**Findings:** Survey results identified frequencies of general and discrete implementation strategies endorsed by site (Table 1) and role (Table 2). Overall, staff felt “very satisfied” with the implementation strategies used within their agencies. Content analysis of qualitative data revealed three salient themes related to implementation strategy use—context, communication, and successes/challenges—providing in-depth detail on how strategies were utilized (by site and role) and strategy effectiveness based on staff experiences. Recommendations for improvement were also collected to improve strategy use within sites. Using a joint display, **QUAN and QUAL** data integrate frequency of strategies with details on quality of use as described by each role and site.

**Implications for D&I Research:** Findings demonstrate the ISSS’s utility in identifying and evaluating implementation strategies utilized to implement EIBIs across settings providing services to children with autism. Reporting implementation strategies using an existing taxonomy offers insight into providers’ perspectives, satisfaction and experiences with agency implementation use that can be compared and tested in other settings. Qualitative findings provide insight into quality of implementation strategies utilized, which can generate more relevant and responsive strategies for community-based settings. Increasing agency staff’s capacity to conceptualize, measure and track implementation strategies is critical to the advancement of D&I science.

**Tables and References available here**

**Primary Funding Source:** Michigan Department of Health and Human Services

### S-75 Identifying facility-level conditions linked to higher reach: A configurational analysis of obesity treatment options offered by VA medical centers

#### Edward Miech^1^, Michelle Freitag^2^, Richard Evans^3^, Jennifer Burns^4^, Laura Damschroder^3^

##### ^1^Veterans Health Administration, VA PRIS-M QUERI, Indianapolis, IN, USA; ^2^Veterans Health Administration, VA PROVE QUERI, Ann Arbor, MI, USA; ^3^Veterans Health Administration, VA Ann Arbor Healthcare System, Ann Arbor, MI, USA; ^4^Veterans Health Administration, VA Center for Clinical Management Research, Ann Arbor, MI, USA

###### **Correspondence:** Edward Miech (edward.miech@va.gov)

**Background:** Implementation science often involves evaluation of complex phenomena where no single factor alone can explain an implementation outcome. Configurational methods offer a mathematical approach to data analysis based on Boolean algebra and set theory that remains relatively new within implementation science. Unlike other methods, configurational analysis expressly allows for causal complexity (when several conditions must jointly appear for an outcome to occur), as well as equifinality (when multiple pathways lead to the same outcome), making it well-suited for implementation-related evaluations where results may depend heavily on local context. We applied configurational methods across a national sample of VA medical centers to identify facility-level conditions directly linked to higher reach in obesity treatment.

**Methods:** After linking facility-level data from the national VA Healthcare Analysis and Information Group (HAIG) survey of obesity treatment options (77 items fielded in March 2017) with FY17 facility-level weight management outcome data from the VA Corporate Data Warehouse, we applied configurational methods to identify facility-level conditions associated with higher reach. There were 125 VA medical centers that responded to the HAIG survey and had complete facility-level weight outcome data. Reach scores were calculated by taking the number of “new” weight management program (MOVE!) visits in 2017 at a VA facility and dividing by the total number of Veterans at that facility times 100. The configurational analysis compared the obesity treatment options offered by the VA facilities in the upper 40% of reach scores (n=50) with those in the lower 40% (n=50). Analysis was conducted using the R package for Coincidence Analysis (“cna”).

**Findings:** The overall model explained 80% (40/50) of the higher-reach facilities with 87% consistency (40/46). The model consisted of 5 distinct pathways, where each included at least one context-dependent condition, such as an experienced pharmacist, a MOVE! coordinator working >20 hours/week, or VA facility complexity level.

**Implications for D&I Research:** Configurational methods identified specific combinations of facility-level conditions distinguishing higher-reach from lower-reach facilities, which included local context. Configurational methods can potentially help address two long-standing challenges in implementation science: accounting for local context in cross-case analyses and determining how the joint presence of specific conditions links directly to an implementation outcome.

**Primary Funding Source:** Department of Veterans Affairs

### S-76 Using a novel request for partners process to recruit community partners for an implementation research study

#### Lucia Leone^1^, Christina Kasprzak^2^

##### ^1^University at Buffalo, Buffalo, NY, USA; ^2^University at Buffalo School of Public Health, BUFFALO, NY, USA

###### **Correspondence:** Lucia Leone (lucialeo@buffalo.edu)

**Background:**

Identifying community organizations to implement research-tested interventions can be a challenging and lengthy process due to the relationship building that is needed. Partners are also often chosen based on convenience, rather than capacity or diversity. Our prior efficacy study of a mobile produce market model (Veggie Van), took 38 months to conduct outreach with 53 potential sites. Prolonged timeline was cited as a barrier to implementation among sites that chose not to implement. Streamlined processes are needed to identify qualified, diverse, and invested partners to implement evidence-based programs.

**Methods:**

We used a Request for Partners (RFP) approach to recruit partners to participate in a cluster-randomized hybrid implementation-effectiveness study of the Veggie Van model. The RFP process was a multi-stage process including formative work to inform RFP development, creation of a selection committee, an intent-to-apply round, a full application round, and an in-person training and selection process. Chosen study partners receive a toolkit, training, technical assistance and funding to offset the costs of implementing the Veggie Van model and assisting with evaluation. Data was collected to characterize applicant size, location and experience; surveys were conducted before and after the in-person training to understand its effectiveness.

**Findings:**

We received 59 intent-to-apply submissions, of which we invited 28 organizations to complete full applications; 17 submitted application and 12 finalists were invited to the in-person training and selection process. The RFP process took approximately 8 months to recruit 9 organizations and 32 community sites across 5 states with a range of size and experience. The process increased understanding of the intervention and partner responsibilities; for example, 63.6% of finalists reported being very to extremely familiar with the Veggie Van model post-training compared to 28.6% pre-training. Only one partner organization dropped out (due to significant organizational change).

**Implications for D&I Research:**

An RFP process is familiar to even small community-based organizations who are accustomed to competing for grant funding, but may not have prior research experience. This RFP reduced partner recruitment timelines, increased partner diversity and cultivated community among mobile market organizations. It may also improve study completion and enhance fidelity.

**Primary Funding Source:** National Institutes of Health

### S-77 A mixed-methods, multi-level remote implementation process evaluation: A viable model in the era of remote conduct of implementation science trials

#### Andrea T. Duran^1^, Jennifer Mizhquiri Barbecho^1^, Darlene Straussman^1^, Othanya Garcia^1^, Samantha Flores^1^, Maria Serafini^1^, Julia Ellis^1^, Julia Menzies^1^, Amy Jones^2^, Siqin Ye^1^, Nathalie Moise^1^

##### ^1^Columbia University Irving Medical Center, New York, NY, USA; ^2^New York State, Office of Mental Health, Albany, NY, USA

###### **Correspondence:** Andrea T. Duran (atd2127@cumc.columbia.edu12)

**Background:** COVID-19 demanded remote healthcare and intervention delivery, necessitating dynamic implementation process evaluation frameworks. Using an AHRQ funded study of a multi-level electronic shared decision making (eSDM) implementation strategy for sustaining Collaborative Care (CC) as a use case, we describe how we adapted our mixed-methods evaluation plan for the era of telehealth.

**Methods:** We adapted our transtheoretical evaluation plan for remote conduct. For RE-AIM (Reach, Effectiveness, Adoption, Implementation, Maintenance) we 1) leveraged the EHR (i.e., EPIC) for outcomes (e.g., reach, fidelity) and 2) added the Stages of Implementation Completion framework to remotely monitor and evaluate eSDM implementation activities. Our original plan employed the Theoretical Domains Framework and the Consolidated Framework for Implementation Research (CFIR) to identify multi-level determinants of CC and eSDM implementation, which we adapted to include 1) Zoom-directed multi-level focus groups/interviews; 2) Qualtrics and phone-administered surveys, and 3) remote observations of inner/outer context (e.g., clinic/ provider workflow) using emails, websites, and EPIC abstraction. To elicit feedback on our remote process evaluation, we employed a human-centered design/rapid cycle model with diverse stakeholders (patients/providers, marketing experts, researchers).

**Findings:** In the pre-implementation period of the AHRQ study, we extracted 12965 charts, abstracted 25 clinic/patient-level metrics and reviewed 100 emails and 5 websites. We determined we could feasibly measure reach (i.e., demographics), adoption (i.e., email-correspondence), effectiveness (tele-CC uptake), implementation (i.e., eSDM fidelity) and context (i.e., external policies, networks-communication, and leadership engagement via emails/websites). Measures that proved infeasible to collect remotely included race, ethnicity, depressive symptoms, and visit wait-times due to unreliable administration/documentation in EPIC, and the quality component of fidelity and select CFIR constructs (e.g., culture, stress) due to inability to observe non-written/verbal communication (e.g., email tone) and workflow. Based on feedback from our stakeholders, we refined our implementation process evaluation to include: 1) remote real-time observation of phone or video visits; 2) virtual clinic-level chart abstraction (e.g., EPIC department-level statistics); 3) application of rigorous content analysis methods to emails/websites.

**Implications for D&I Research:** We demonstrate key determinants to adapting and combining existing evaluation frameworks to enable mixed-method, multi-level remote implementation process evaluation for the telehealth era.

**Primary Funding Source:** Agency for Healthcare Research and Quality

### S-78 The roll-out implementation optimization (ROIO) design: Rigorous testing of a data-driven implementation improvement aim

#### J.D. Smith^1,2^, C. Hendricks Brown^3^

##### ^1^Psychiatry and Behavioral Sciences and Preventive Medicine, Northwestern University Feinberg School of Medicine, Chicago, IL, USA; ^2^University of Utah School of Medicine, Salt Lake City, UT, USA; ^3^Northwestern University Feinberg School of Medicine, Chicago, IL, USA

###### **Correspondence:** J.D. Smith (jd.smith@northwestern.edu)

**Background:** D&I science and quality improvement (QI) share a number of objectives and natural synergies, yet often differ in scope, rigor, and type of design. Whereas QI favors generation of local knowledge through small-n designs, D&I strives for generalizable causal inference. In this presentation, we describe a new design for situations where local knowledge and generalizability are simultaneously sought, such as the learning health system (LHS).

**Methods:** The roll-out implementation optimization (ROIO) design involves crossovers where clusters of units (e.g., clinics) begin in one condition and move to another, with staggered crossover points determined prospectively with units (randomly) assigned to positions in the sequence. The “optimization” feature is planful and prospective and uses early quantitative and qualitative data to inform strategy tailoring for subsequent units in the roll-out. This contrasts with the standard method for roll-out trials in which the strategies are held constant across units and variation is unplanned and treated as protocol violations or studied as adaptation. In the ROIO design, the goal is to improve implementation with each successive roll-out to arrive at the most effective strategy to take to scale. Methodologic challenges with specific implications for the ROIO design are (1) appropriate timing of the roll-out schedule (sufficient time between clusters), (2) selection of outcome metrics (sensitive to change) and meaningful (criterion-based), (3) measurement and evaluation that is rapid and informative of needed changes, and (4) analysis using continuous (multilevel models, time-series analysis) and criterion-based approaches (non-parametric tests) that account for cluster serial independence.

**Findings:** Power analysis of an illustrative trial to test guideline-adherent diagnosis of pediatric hypertension in community health centers indicated feasibility of this design using both criterion-based and continuous outcomes with a feasible number of units (n=32; 4 clusters of 8 units) and within a 5-year grant period. Engagement of partner healthcare organization in a LHS indicated high degree of acceptability for the ROIO design.

**Implications for D&I Research:** The ROIO design can be used harmoniously within and in partnership with LHSs, lead to an optimal implementation strategy, and satisfy the needs and goals of partner agencies who might otherwise be reluctant to participate in implementation research.

**Primary Funding Source:** National Institutes of Health

### S-79 Systems science for implementation interventions: An application to tobacco smoking cessation for persons with serious mental illness

#### Wanyu Huang^1^, Chia-Hsiu Chang^1^, **Takeru Igusa**^1^, Elizabeth Stuart^2^, Nae-Yuh Wang^1^, Emma McGinty^2^, Gail Daumit^3^

##### ^1^Johns Hopkins University, Baltimore, MD, USA; ^2^Johns Hopkins Bloomberg School of Public Health, Baltimore, MD, USA; ^3^Johns Hopkins University School of Medicine, Baltimore, MD, USA

###### **Correspondence:** Takeru Igusa (tigusa1@jhu.edu)

**Background:** Implementation of complex interventions addressing diverse populations, tasks and environments through sophisticated strategies is challenging in public health. Trials with adaptive and other innovative designs have been useful for some implementation research questions, but randomized trials large enough to address multiple contextual factors are often prohibitively resource intensive. Systems science models have been proposed to help guide, improve, and scale-up complex implementation processes. Such models have not yet been widely used in implementation science, partly because they are not straightforward to develop.

**Methods:** We propose a stepwise procedure for developing systems science models and show how this procedure can be used to address implementation research questions using a case example of improving delivery of evidence-based smoking cessation practices for persons with serious mental illness in community mental health clinics. We focus on microsimulation and agent-based modeling to simulate the mechanism and components of complex intervention to highlight dynamics during implementation and gain insights into the implementation process.

**Findings:** Two detailed examples were used to demonstrate the approach. First, we used microsimulation to model observed tobacco smoking behaviors of persons with serious mental illness in a recently completed NIH-funded randomized trial. This provided insights into how the time-evolution of willingness to quit smoking impacted intervention outcomes. Second, we demonstrated how agent-based modeling can be used to develop a dashboard to provide system-level guidance for the implementation strategies. The dashboard includes levers for simulating what-if scenarios that can be used to guide site-level implementation decisions for provider-level training while incorporating system and external factors such as treatment reimbursement policy and workforce availability.

**Implications for D&I Research:** In the implementation science field, there is a pressing need to understand and address complexity accounting for multiple intervention barriers at the patient, provider, organizational and policy levels. We show how systems science models can provide insights into some of the processes in complex interventions, and simulate what-if scenarios to examine intervention options and guide implementation decisions for improving delivery of evidence-based practices in community mental health clinics. A stepwise procedure for developing such models is proposed to make such modeling efforts accessible to implementation scientists.

**Primary Funding Source:** National Institutes of Health

### S-80 Multi-tiered external facilitation: The role of feedback loops and tailored interventions in supporting change in quality processes in a stepped-wedge implementation trial

#### Lauren S. Penney^1,2^, Teresa Damush^3^, Nicholas Rattray^4^, Edward Miech^5^, Barbara Homoya^6^, Sean Baird^7^, Laura Myers^8^, Dawn Bravata^9^

##### ^1^Medicine, University of Texas Health San Antonio, San Antonio, TX, USA; ^2^Veterans Health Administration , South Texas Veterans Affairs Healthcare System, San Antonio, TX, USA; ^3^Veterans Health Administration , VA Headache Centers of Excellence Research and Evaluation Center, West Haven, CT, USA; ^4^Veterans Health Administration, VA HSR&D Center for Health Information and Communication (CHIC)/PRIS-M QUERI; Richard L. Roudebush VA Medical Center, Indianapolis, IN, USA; ^5^Veterans Health Administration , VA PRIS-M QUERI, Indianapolis, IN, USA; ^6^Veterans Health Administration , Roudebush VA Medical Center, Indianapolis, IN, USA; ^7^Regenstrief Institute, Indianapolis, IN, USA; ^8^Veterans Health Administration, Health Services Research and Development Service, Indianapolis, IN, USA; ^9^Veterans Health Administration , PRIS-M QUERI/Indiana University School of Medicine, Indianapolis, IN, USA

###### **Correspondence:** Lauren S. Penney (Lauren.Penney@va.gov)

**Background:** Facilitation is a complex, relational implementation strategy that guides change processes. The methods by which facilitators come to know about and make sense of contextual factors that prompt tailoring activities have not been well-described.

**Methods:** We conducted a mixed methods evaluation of a trial to improve the quality of transient ischemic attack care. Six sites in the Veterans Health Administration received external facilitation (EF) before and during a 1-year active implementation period. We examined how EF was employed and activated. Data analysis included prospective logs of facilitator correspondence with sites (160 site-directed episodes) and collaborative call debriefs (n=22). Logs were descriptively analyzed across facilitators, sites, time periods, and activity types. Debriefs were reviewed to identify instances of and utilization of EF during site critical junctures.

**Findings:** Multi-tiered EF at the six sites was supported by three groups (1. site-facing quality improvement [QI] facilitators and the 2. data and 3. implementation cores) that were connected by feedback loops to guide process changes. Multiple forms of longitudinal data (e.g., monthly collaborative call site updates, periodic site visits by the implementation core, monthly administrative data pulls by the data core, chart reviews by the QI nurse) were reflected on and evaluated by the EF team to sensemake about each site. Debriefing exercises conducted between the QI facilitators and cores generated ideas for tailored EF and site redirection. Each site received an average of 24 episodes of site-directed EF; most of the EF was delivered by the QI nurse. Site-directed EF varied widely across sites and time periods in terms of these facilitation types. External facilitators used monitoring and dialogue to intervene by facilitating redirection when sites faced implementation barriers. External facilitators, in collaboration with the implementation and data cores, successfully used strategies tailored to diverse local contexts, including networking, providing data, and brainstorming solutions.

**Implications for D&I Research:** This work contributes to understanding how feedback loops and interdisciplinary sensemaking can support EF tailoring to support change in quality processes. Critical juncture cases illustrate the complexity of EF and the necessity of multiple strategies in combination to facilitate implementation progress.

**Primary Funding Source:** Department of Veterans Affairs

### S-81 Implementing an evidence based dementia program in PACE settings: A protocol for tracking adaptation and fidelity

#### Nancy Hodgson^1^, Laura Gitlin^2^

##### ^1^University of Pennsylvania, Philadelphia, PA, USA; ^2^Drexel University, Philadelphia, PA, USA

###### **Correspondence:** Nancy Hodgson (hodgsonn@nursing.upenn.edu)

**Background:** Despite over 200 evidence-based dementia caregiver programs we know little about whether these programs are scalable and for which service contexts, nor do we fully understand the most effective approaches of ensuring successful implementation. As a result, a small fraction of the 15+ million dementia caregivers in the US have access to evidence-based programs. One essential question to moving dementia care interventions into practice is,“What is the optimal balance between fidelity to and adaptation of a proven dementia program in “real world” long term care settings?"

**Methods:** This presentation describes the methods and measures that are being used to capture the adaptation/fidelity and implementation of an evidence based dementia care program (Care of Persons in their Environment or COPE) in Programs of All-Inclusive Care for the Elderly (PACE) settings. The ‘COPE in PACE’ study is an ongoing, 5-year, multisite pragmatic trail being conducted in10 PACE settings throughout the US (NCT04165213).

**Findings:** During pre-implementation the science-based elements of COPE were documented including the theory of change, logic model and core components. A due diligence review was conducted to identify fidelity/adaptation challenges. Adaptability in COPE program delivery characteristics were then identified pre-implementation and included elements of program structure (sequence of sessions), content (assessments), and methods of delivery (online). During implementation, the documentation of implementation strategies is being captured using a checklist derived from in the Expert recommendations for implementing change (ERIC) workgroup. Ongoing documentation of fidelity/adaptation aspects of program implementation is being conducting using the FRAME expanded framework for reporting adaptations and modifications to evidence-based interventions. Data collection methods include observation, fidelity checklists, focus groups, interviews and surveys.

**Implications for D&I Research:** Program adaptation/fidelity and implementation details should be clearly documented to enhance scalability. Understanding the methods and measures deployed in adaptation and implementation of evidence based dementia programs can help guide future translation efforts, and inform the design of new family caregiver support programs to optimize their potential.

**Primary Funding Source:** National Institutes of Health

### S-82 Making sense of adaptations in the veteran health administration's rural transitions nurse program: Refining methodology and pragmatic implications for scale-up

#### Michaela McCarthy^1^, Mary Nunnery^2^, Lexus Ujano-De Motta^3^, Chelsea Leonard^2^, Borsika Rabin^4^

##### ^1^Veterans Health Administration ,Seattle/Denver Center of Innovation, Rocky Mountain Region Veterans Administration Medical Center ,Denver, CO, USA; ^2^Veterans Health Administration, Rocky Mountain Regional VA Medical Center, Aurora, CO, USA; ^3^Veterans Health Administration, Seattle-Denver Center of Innovation, Aurora, CO, USA; ^4^Veterans Health Administration, VA Center of Excellence for Stress and Mental Health, San Diego, CA, USA

###### **Correspondence:** Michaela McCarthy (michaela.mccarthy@cuanschutz.edu)

**Background:**

In the context of the implementation of complex health services interventions in real-world settings, adaptations are inevitable. Adaptations are changes to an intervention, implementation strategy, or context that are meant to improve the fit to the local context before, during, and after implementation. There is a growing interest in understanding how to systematically document and make sense of adaptations including what changes, why, when, by whom, and with what impact. The rural Transitions Nurse Program is a care coordination program with the Veterans Health Administration, designed to safely transition a rural Veteran from a tertiary hospital to home and was implemented across 12 sites nationwide over four years. We will present findings on adaptations from the first cohort (5 sites) of our study and discuss methodological and scale-up implications.

**Methods:**

Adaptations were systematically documented using multiple approaches: a real-time database and semi-structured midpoint and exit interviews with providers, and member checking with the implementation team. Interviews were recorded and transcribed verbatim. Adaptations were identified from the various sources using the RE-AIM expanded version of the original Stirman framework. We organized adaptations by the timing of adaptation (pre-, early, mid, late implementation and sustainment) and coded whether they were proactive or reactive. Findings were based on the triangulation of data from the multiple data collection approaches.

**Findings:**

A total of 41 unique adaptations were reported during the 30-month study period. The most common types of adaptation were changes in target populations (enrollment criteria) and personnel changes (staff turnover). There was variation in the number and type if adaptations that were observed across the time points with most adaptations emerging mid-implementation. Comprehensive details for all categories of the adaptation framework will be presented across the various time points.

**Implications for D&I Research:**

We found that it was feasible to systematically document adaptations using multiple methods and triangulate findings. Providers were able to track adaptations real time across the course of an intervention providing timely and actionable feedback to the implementation team overseeing national roll-out. Longitudinal semi-structured interviews can complement the real-time database and elicit reflective adaptations.

**Primary Funding Source:** Department of Veterans Affairs

### S-83 A randomized trial to identify accurate and cost-effective fidelity measurement methods for cognitive-behavioral therapy: Project facts preliminary findings

#### Emily Becker-Haimes^1^, Steven Marcus^1^, Melanie Klein^1^, Sonja Schoenwald^2^, Shannon Dorsey^3^, David Mandell^1^, Judy Shea^1^, Bryce McLeod^4^, Perrin Fugo^1^, Rinad Beidas^5^

##### ^1^University of Pennsylvania, Philadelphia, PA, USA; ^2^Oregon Social Learning Center, Eugene, OR, USA; ^3^Psychology, University of Washington, Seattle, WA, USA; ^4^Virginia Commonwealth University, Richmond, VA, USA; ^5^University of Pennsylvania Perelman School of Medicine, Philadelphia, PA, USA

###### **Correspondence:** Emily Becker-Haimes (embecker@upenn.edu)

**Background:** Research to improve implementation outcomes in community mental health is hindered by an inability to accurately and inexpensively measure therapist fidelity to evidence-based treatments. In implementation science evaluation frameworks, fidelity is an indicator of care quality and an important mechanism by which treatment results in positive outcomes. Despite its importance, few fidelity measurement methods have demonstrated efficiency, reliability, and validity.

**Methods:** This randomized trial compared the accuracy and effectiveness of three methods of assessing fidelity to cognitive-behavioral therapy (CBT). We recruited 126 therapists from 27 community agencies in the City of Philadelphia. Therapists were randomized to one of three fidelity conditions: 1) enhanced self-report (therapist completes a self-report measure on the CBT interventions used in session developed in partnership with community stakeholders), 2) chart-stimulated recall (therapist reports on CBT used in session via an interview with a trained rater, with the chart available to assist reporting accuracy), and 3) behavioral rehearsal (therapist demonstrates CBT used in session via role-play with a trained rater). Each therapist provided data for approximately three unique sessions (client *n* = 304). All sessions were audio-recorded and coded using the Therapy Process Observational Coding System for Child Psychotherapy-Revised Strategies scale. We compared each condition to the gold standard of direct observation of therapist session behavior to determine which most accurately assesses fidelity.

**Findings:** Data collection is complete; analyses are in progress. Initial analyses will present psychometric performance of the self-report, chart-stimulated recall, and behavioral rehearsal measures. Three-level mixed effects regression will compare the performance of each novel fidelity condition to direct observation and the relative performance of each novel condition to another. Initial results suggest that behavioral rehearsal produces comparable fidelity scores to direct observation, and self-report and chart-stimulated recall yield inflated fidelity scores.

**Implications for D&I Research:** Results will provide needed information on how to accurately measure therapist fidelity to CBT for youth. Findings will inform fidelity measurement practices in future implementation studies as well as recommendations for fidelity monitoring in clinical practice.

**Primary Funding Source:** National Institutes of Health

### S-84 A study to determine the external validity of the Iowa implementation for sustainability framework

#### Laura Cullen^1^, Kirsten Hanrahan^1^, Stephanie Edmonds^2^, Heather Reisinger^3^, Michele Wagner^2^

##### ^1^University of Iowa Hospitals and Clinics, Iowa City, IA, USA; ^2^University of Iowa Hospitals & Clinics, Iowa City, IA, USA; ^3^University of Iowa Healthcare, Iowa City, IA, USA

###### **Correspondence:** Laura Cullen (laura-cullen@uiowa.edu)

**Background:** An application-oriented implementation framework designed for clinicians and based on the Diffusion of Innovation theory, identified 81 implementation strategies with suggested timing for use within four implementation phases. The purpose of this research was to strengthen the framework for clinician use and determine external validity.

**Methods:** A three-step, iterative approach guided revisions. First: Individuals (n=1,578) requesting use of the framework over the previous seven years were sent an electronic questionnaire. Evaluation captured usability, generalizability, accuracy, and implementation phases for each strategy. Second: Nurse leaders who use the framework pile sorted strategies for multidimensional scaling and hierarchical analysis. Third: EBP/implementation experts (panel=5) used data and built consensus to strengthen the framework.

**Findings:** Participants completing questionnaires (n = 127, 8% response) were predominately nurses (94%), highly educated (94% Master’s or higher), from across healthcare (52% hospital/system, 31% academia, and 7% community) in the U.S. (84%). 95% reported experience using the framework. A four-point scale (1 = not/disagree to 4 = very/agree) was used, combined ratings 3 and 4 are reported. The framework was deemed useful (92%), easy to use (72%), intuitive (67%), generalizable (100%), flexible and adaptive (100%), with accurate timing (96%), and accurate targets (100%). 88% would use the framework again. 51 participants identified implementation strategy timing within four phases, which was analyzed for significance (Cochran’s Q) - of 81 strategies, 54 were (66.7%, p<0.05) significantly linked to a specific phase; of these 30 (55.6%) matched the original framework. Next: nurse leaders (n=23) completed a pile sorting task; Anthropac software created a concept map and hierarchical clusters with four groups. Lastly: Experts used these data and implementation science to refine and specify each strategy, identifying actors, function, timing targets, and groups. Strategy usability, timing, and groupings were used to refine the framework.

**Implications for D&I Research:** The Iowa Implementation for Sustainability Framework offers a typology to guide implementation for healthcare improvements. This study specifies 77 implementation strategies and begins to establish external validity for the framework. Standard use of strategy names is foundational to compare and understand when implementation strategies are effective, in what dose, for which topics, by whom, and in what context.

### S-85 Validating and enhancing the clinical sustainability assessment tool: A quick assessment for researchers and practitioners

#### Sara Malone^1^, Virginia Mckay^2^, Kim Prewitt^3^, Justin Smith^4^, Asya Agulnik^5^, Douglas Luke^6^

##### ^1^Washington University in St. Louis, Saint Louis, MO, USA; ^2^School of Social Work, Washington University in St. Louis, St. Louis, MO, USA; ^3^Washington University in St. Louis, St. Louis, MO, USA; ^4^Northwestern University Feinberg School of Medicine, Chicago, IL, USA; ^5^St. Jude Children’s Research Hospital, Memphis, TN, USA; ^6^Center for Public Health Systems Science, Washington University in St. Louis, Saint Louis, MO, USA

###### **Correspondence:** Sara Malone (sara.malone@wustl.edu)

**Background:** Few validated assessment tools are available to support sustainable implementation, an increasingly recognized need among clinicians. We describe the validation of the Clinical Sustainability Assessment Tool, or CSAT, and multiple versions of the CSAT available to assess sustainability that are accessible for both researchers and practitioners in clinical settings.

**Methods:** We recruited clinicians from 25 clinics across the cancer care continuum. All participants were asked to complete the CSAT with a specific practice or program in mind in addition to questions about the individual, organization, practice. The CSAT is a 35-question measure, consisting of seven domains, with each question asking a Likert-type response from little to no extent (1) to a great extent (7). Respondents also answered validating questions from the Change Process Capability Questionnaire, a quality improvement measure aimed to assess stages of change management within a clinical setting. A CSAT score is generated as a simple average of responses overall and for each subdomain. A confirmatory factor analysis will be conducted to confirm the validity and reliability of the seven CSAT domains. Further validation will be demonstrated by relating CSAT scores to other theoretically relevant constructs, including organizational size, resource availability, and readiness for change.

**Findings:** We will present the reliability and validity of the seven subdomains within the CSAT, and the association between CSAT scores and organizational readiness for change. In addition, we will present two new extensions of the CSAT. The first is focused on an abbreviated version of the CSAT for use among clinical settings working to implement electronic medical record trigger systems. Second, we developed a Spanish version of the CSAT for use in predominantly Spanish-speaking communities. The tool is currently being the piloted with a number of clinicians in pediatric oncology centers in Central and South America.

**Implications for D&I Research:** Our work meets a clear demand for valid, reliable measures of clinical capacity for sustainable implementation. This is the only validated sustainability assessment tool as well as the only tool available in Spanish. We recommend the CSAT for researchers and clinicians interested in assessing clinical capacity, and the CSAT is currently available at sustaintool.org.

**Primary Funding Source:** National Institutes of Health

### S-86 Sufficient pathways for maintaining prevention programs long-term: A configurational approach using fuzzy-set qualitative comparative analysis

#### Sapna Mendon-Plasek^1^, Lawrence Palinkas^2^, Michael Hurlburt^3^, Juan Villamar^4^, C. Hendricks Brown^5^

##### ^1^Columbia University, New York, NY, USA; ^2^Social Work, University of Southern California, Los Angeles, CA, USA; ^3^University of Southern California, Los Angeles, CA, USA; ^4^Northwestern University Feinberg School of Medicine, Chicago, IL, USA; ^5^Northwestern University, Chicago, IL, USA

###### **Correspondence:** Sapna Mendon-Plasek (smendon@usc.edu)

**Background:** An understanding of what determines sustainment is critical to identifying strategies designed to improve long-term maintenance of evidence-based practice (EBP) in community-based organizations. Several elements such as planning for sustainment, program champions, coalition support, and meeting the needs of the community, have emerged as key facilitators of sustainment. However, how these elements are interrelated within a complex social system remains unknown; in other words, how do they function when combined with one another to produce desired sustainment?

**Methods:** Based on a larger parent-study, the current study included representatives from 145 grantee sites funded by seven SAMHSA-funded programs focused on reducing or preventing various mental health and substance-use related disorders among at-risk youth. A set-theoretic approach, fuzzy-set Qualitative Comparative Analysis (fsQCA), was used to identify configurations of conditions that, when combined in causal pathways, are sufficient for producing sustainment. Fuzzy-set QCA was conducted to assess five conditions derived from the Consolidated Framework for Implementation Research (CFIR): financial stability; responsiveness to community needs and values; coalitions, partnerships, & networks; organizational capacity and staff capability; and characteristics of the implementation process. Membership sets were calibrated on a 5-point scale. Following an analysis of necessity to determine which conditions are a superset of sustainment, sufficiency analysis was conducted to determine which configurations are a subset of sustainment.

**Findings:** Initial minimization based on Boolean logic found 14 configurations of a possible 32 to be relevant. Standard analysis found two configurational pathways with high consistency and moderate coverage, indicating that when these particular constructs combine, they are, as a unit, sufficient to produce sustainment: 1) community responsiveness and organizational capacity when combined with process, and 2) community responsiveness and organizational capacity when combined with coalitions, networks, and partnerships.

**Implications for D&I Research:** By applying novel non-linear methodology, this study moved beyond identifying individual factors that predict sustainment to identify the interdependent relationships of these elements within complex social infrastructures. Such knowledge assigns value to specific CFIR determinants and helps to prioritize which aspects can further guide implementation efforts in practice. Knowing which pathways are sufficient for sustainment can inform the selection of implementation strategies and may be a cost-savings approach.

**Primary Funding Source:** National Institutes of Health

### S-87 Illuminating the dimensions of bridging factors that link outer and inner context during EBP implementation and sustainment

#### Rebecca Lengnick-Hall^1^, Nicole Stadnick^2,3,4^, Kelsey Dickson^4^, Joanna Moullin^5^, Gregory Aarons^6^

##### ^1^Washington University, St. Louis, MO, USA; ^2^UC San Diego Dissemination and Implementation Science Center, La Jolla, CA, USA; ^3^University of California San Diego, La Jolla, CA, USA; ^4^Child and Adolescent Services Research Center, San Diego, CA, USA^5^Western Australia, Curtin University, Perth, Australia; ^6^University of California, San Diego, La Jolla, CA, USA

###### **Correspondence:** Rebecca Lengnick-Hall (rlengnick-hall@wustl.edu)

**Background:** Bridging factors are relational ties (e.g. partnerships), formal arrangements (e.g. contracts), or processes (e.g. data sharing agreements) that connect outer system and inner organizational contexts and may be critical drivers of evidence-based practice (EBP) implementation and sustainment. Bridging factors were recently defined in the Exploration, Preparation, Implementation, Sustainment (EPIS) framework but require further identification and specification to advance implementation models, measures and methods. More specifically, understanding interconnections between outer and inner contexts is important for successful EBP sustainment. Current implementation strategy research does not account for the complex interplay between outer and inner contexts. And, bridging factors are relevant to any framework with an outer and inner context or setting. Our goal is to advance the bridging factors construct by identifying relevant dimensions and presenting illustrative case studies.

**Methods:** We used a multiple case study design (N=12 cases) representing different bridging factor examples. Inclusion criteria: (1) clearly distinguishable outer and inner contexts/settings (2) identifiable bridging factor(s) (3) sufficient information to describe how the bridging factor affected implementation. We used iterative qualitative synthesis to develop a list of dimensions that define bridging factors and to assess case study data. Case information was entered into a matrix whereby dimensions comprised the rows and cases comprised the columns. We then collectively considered, and independently coded each dimension as one that describes the function or form of the bridging factor. The senior author was a tiebreaker for discrepancies.

**Findings:** We drew upon the concepts of functions and forms, a distinction that was originally proposed in the complex health intervention literature. We conceptualize “function bridging factor dimensions” as characteristics that describe the goal of the bridging factor related to EBP implementation and sustainment. We conceptualize “form bridging factor dimensions” as specific structures and/or activities that illustrate how the bridging factor was tailored for a local implementation project. Our final inventory included five function and nine form dimensions.

**Implications for D&I Research:** Enhanced specification and operationalization of bridging factors can significantly advance models, measures and methods because it offers researchers and practitioners a new lens for proactively investigating and leveraging linkages across outer and inner contexts during implementation efforts.

**Primary Funding Source:** National Institutes of Health

## Prevention and Public Health

### S-88 Moving towards the development of standard food insecurity screening practices in primary care settings: An embedded multiple case study

#### Sabira Taher

##### Northwestern University Feinberg School of Medicine, Chicago, IL, USA

###### **Correspondence:** Sabira Taher (sabira.taher@northwestern.edu)

**Background:**

Food insecurity is a social determinant of health that prevents low-income Americans from accessing healthy food in safe and socially acceptable ways. It contributes to difficulty with disease management and poor health outcomes. National policy advocates support universal application of the validated Hunger Vital Sign^TM^ tool in routine primary care to identify and connect at-risk patients to healthy food resources. Small-scale clinical screening initiatives have recently surfaced, yet, the lack of standard implementation guidelines limits wide-scale dissemination of these practices. The purpose of this study was to examine common implementation facilitators and barriers of two food insecurity screening initiatives in a diverse jurisdiction, guided by The Consolidated Framework for Implementation Research (CFIR).

**Methods:**

This was an embedded multiple case study that included five primary care clinics across two healthcare organizations in Chicago and Suburban Cook County. A theoretical sampling approach was used to select healthcare professionals (e.g., providers, medical directors and liaisons to community organizations) for participation in semi-structured interviews. Using a guide informed by CFIR and existing literature, n=19 interviews were conducted until data saturation was achieved. A cross-case thematic analysis was performed to identify salient implementation factors.

**Findings:**

Common implementation barriers reported by participants were lack of human and financial capital, rigid workflow processes and lack of physical space associated with the inner healthcare setting. Program participants reported that implementation was facilitated by cosmopolitanism (i.e. existing community collaborations) and clinic-level autonomy that effectively enhanced intervention adaptability and testability. These specific intervention characteristics fostered program support and sustainability that maybe replicated in other settings.

**Implications for D&I Research:**

Study findings were used to develop a formative conceptual model that establishes a foundation for the development of standard food insecurity screening practices in primary care settings. The study was innovative in its application of CFIR and the use of clinical stakeholders engagement to understand implementation processes in the face of resource challenges—a common barrier for clinics that treat underserved populations. These findings emphasize the importance of external support and intervention flexibility that maybe built upon and rigorously tested for wide-scale dissemination of screening practices.

**Primary Funding Source:** University of Illinois at Chicago

### S-89 Organizational characteristics associated with adoption and penetration of classroom physical activity in a national sample of US elementary schools

#### Blake Densley, Michaela McQuilkin, Hannah Calvert, Peter Boedeker, Lindsey Turner

##### Boise State University, Boise, ID, USA

###### **Correspondence:** Blake Densley (blakedensley@u.boisestate.edu)

**Background:** Classroom-based physical activity—including brief physical activity breaks (PABs), or physically active lessons (PALs)—can benefit student health, and is considered an evidence-based best practice. However, adoption of this practice is uneven across schools and penetration rates at the classroom level are rarely examined. Further, little research has examined implementation facilitators such as leadership, financial support, and the role of champions.

**Methods:** These analyses use survey data gathered from respondents at a nationally-representative sample of 559 US public elementary schools in 2019-20. Items assessed adoption of PALs (yes, no/DK), and PABs (yes, no/DK). Penetration was calculated based on reported number of teachers who use PABs (>50% vs <50%). Implementation facilitators were assessed with three items: 1) does the administrator encourage teachers to use PABs? (never/rarely, sometimes, often); 2) is financial support available to teachers to purchase CBPA curricula/resources? (yes, no/DK); and 3) is there a champion for wellness? (yes, no/DK). Analyses were conducted in a logistic regression framework controlling for school demographics (size, locale, region, racial/ethnic composition, and free/reduced-priced meal eligibility as a proxy for poverty). Analyses were weighted and accounted for sampling strata and clustering of schools within districts.

**Findings:** Adoption of PALs was reported at 81.7% of schools, but varied by implementation factors. In multivariate models, the adjusted prevalence of adopting PALs was 58.8% at schools with no/rare administrator encouragement vs 84.8% at schools where encouragement happened often (p=.001). Adjusted prevalence was 83.1% at schools with financial resources versus 73.7% at schools without (p=.014) and at 82.4% of schools with a champion versus 71.1% of schools without (p=.008). For PABs, while adoption was nearly universal (92.4%), it varied by administrator encouragement, with adoption at 97.8% where encouragement happened often, versus 78.8% where it never/rarely happened. Broader penetration occurred with implementation facilitators; where financial support was available, 63.1% of schools reported that a majority of teachers used PABs, versus 47.7% of schools without financial support.

**Implications for D&I Research:** Organizational adoption of PABs and PALs is facilitated by proactive leadership and resources. Increasing these facilitators in elementary schools may help to further increase penetration at the classroom level.

**Primary Funding Source:** The Robert Wood Johnson Foundation

### S-90 Results from a cluster-randomized hybrid type III implementation trial of obesity prevention practices in head start

#### Taren Swindle^1^, Nicole McBride^2^, James Selig^3^, Janna Martin^4^, Audra Staley^4^, Leanne Whiteside-Mansell^5^, Geoffrey Curran^6^

##### ^1^Univesity of Arkansas for Medical Sciences, Little Rock, AR, USA; ^2^Okinawa, Japan^3^UAMS, Little Rock, AR, USA; ^4^Little Rock, AR, USA; ^5^Family and Preventive Medicine, UAMS, Little Rock, AR, USA; ^6^Department of Pharmacy Practice, University of Arkansas for Medical Sciences, Little Rock, AR, USA

###### **Correspondence:** Taren Swindle (tswindle@uams.edu)

**Background:** This study reports on a cluster-randomized Hybrid III trial designed to test stakeholder-selected implementation strategies to support Together, We Inspire Smart Eating (WISE). The 8 implementation strategies selected by stakeholders and tested in this study included formal leadership commitments, preparation of champions, an implementation blueprint, reminders, educational materials, incentives, and facilitation.

**Methods:** A stratified randomization approach allocated 4 preschool centers serving children in families affected by poverty sites (18 classrooms) to receive enhanced implementation support (i.e., treatment) and 5 sites (20 classrooms) to standard support (i.e., control) before school began. The study was conducted in the 2018-2019 school year, and the Re-AIM framework guided the evaluation. T-test comparisons examined differences between conditions at the end of the school year. Additional multilevel regression analyses are planned that account for nesting of the data structure.

**Findings:** Indicators of Adoption and Implementation were significantly different between groups. Specifically, teachers in the enhanced condition reported greater readiness for change (*p* =.04), fewer barriers to implementation (*p* =.008), and higher feasibility (*p*=.05) and appropriateness (*p*=.03) of WISE. For Implementation fidelity, there were significant differences for 3 of the 4 WISE practices (all *p* < .03, effect sizes > .80). Educators reported reach of lessons was greater in the enhanced group, but not significantly so (69% basic, 76% enhanced). Child carotenoid levels increased in both groups but did not differ between conditions. Changes in child Body Mass Index were not different between groups. The cost of the enhanced implementation averaged $274 per classroom per year.

**Implications for D&I Research:** A set of stakeholder-driven implementation strategies improved the adoption, reach, and implementation of the WISE intervention in Head Start. Engagement of key stakeholders in the selection and tailoring of implementation strategies may prove a key approach to improving uptake of evidence in the early care and education setting.

**Primary Funding Source:** National Institutes of Health

### S-91 Evaluation of intervention and implementation fidelity of healthy me, healthy we

#### **Courtney Luecking**^1^, Amber Vaughn^2^, Regan Burney^2^, Heidi Hennink-Kaminski^2^, Derek Hales^2^, Dianne Ward^3^

##### ^1^University of Kentucky, Lexington, KY, USA; ^2^University of North Carolina, Chapel Hill, NC, USA; ^3^Department of Nutrition and Center for Health Promotion and Disease Prevention, University of North Carolina, Chapel Hill, NC, USA

###### **Correspondence:** Courtney Luecking (courtney.luecking@uky.edu)

**Background:** Public health interventions often target multiple levels of influence and are complex to implement and evaluate. To better understand the success or failure of interventions, it is imperative to evaluate fidelity of these multi-level, multi-faceted interventions and supporting implementation strategies. For this project, we evaluated the fidelity of implementation support and intervention delivery of a multi-level social marketing campaign, *Healthy Me, Healthy We* (HMHW), to identify successes and failures.

**Methods:** The HMHW’s 8-month social marketing campaign promoted healthy eating and physical activity behaviors for 3 – 4-year-old children attending early care and education (ECE) centers. HMHW required two levels of implementation support (research team’s support to ECE providers, ECE providers’ support to parents) and two levels of campaign delivery (ECE providers’ and parents’ delivery to children). Process evaluation data from the 48 centers in the intervention group of the hybrid type I cluster randomized control trial included attendance logs, self-report surveys, observation checklists, and field notes. These data were used to create a 35-item fidelity index of implementation support and intervention delivery.

**Findings:** The research team generally provided implementation support to ECE providers as intended. However, the overall fidelity of implementation support and intervention delivery by ECE providers and parents was low (mean 17.4 out of 35). Sub-scales of the index indicate fidelity decreased with each level of implementation support and intervention delivery – ECE providers’ delivery of HMHW at the center (mean 10.7 out of 16), ECE providers giving implementation support to parents (4 out of 12), and parents’ delivery of HMHW at home (mean 2.7 out of 7).

**Implications for D&I Research:** The decreasing fidelity at subsequent levels of implementation support and intervention delivery observed in this study offers possible explanations for the lack of effect seen in this and other health promotion interventions. Findings demonstrate a need for different or additional strategies to adequately support implementation of multi-level interventions, particularly when individuals are required to both deliver intervention components and support others in doing so. Design, selection, and evaluation of implementation strategies for each level of an intervention should, in the future, explore integration with existing routines, practices, and/or organizational structures.

**Primary Funding Source:** National Institutes of Health

### S-92 Specifying and reporting implementation strategies used in a school-based trauma-informed prevention trial

#### Stephanie Moore^1^, Kimberly Arnold^2^, Tamar Mendelson^3^

##### ^1^University of California, Riverside, Riverside, CA, USA; ^2^University of Pennsylvania Perelman School of Medicine, Philadephia, PA, USA; ^3^Johns Hopkins Bloomberg School of Public Health, Baltimore, MD, USA

###### **Correspondence:** Stephanie Moore (stephanie.moore@ucr.edu)

**Background:** Identifying implementation strategies that can support the adoption, implementation, and sustainment of evidence-based practices in real world settings is pertinent for reducing the research-to-practice gap. Whereas taxonomies of implementation strategies (e.g., Expert Recommendations for Implementing Change (ERIC)) and specification guidelines have been delineated, reporting standards have yet to be widely adopted, especially in educational settings. This study utilized the School Implementation Strategies, Translating ERIC Resources (SISTER) taxonomy and Proctor and colleagues’ recommendations to specify and report implementation strategies used during a school-based efficacy trial (Project POWER) of a trauma-informed prevention program delivered by a university research team with community members and school staff facilitators-in-training.

**Methods:** Following conclusion of the 4-year trial (fall 2019), we scheduled meetings with the core Project POWER research team members (n=10) to identify and operationalize the implementation strategies that supported intervention delivery during the trial. Concepts from leading implementation determinant frameworks were used to code action targets and temporality. We used descriptive statistics to explore and describe the SISTER strategy actions, actors, action targets, temporality, implementation outcomes, and dose.

**Findings:** Thirty-seven SISTER strategies were used in 29 schools during the trial. Most strategies fell in the categories of Train and Educate Stakeholders (n=7), Use Evaluative and Iterative Strategies (n=7), and Develop Stakeholder Interrelationships (n=5). Actors included members of the research team, partner schools, and the local community. Most implementation strategies were used multiple times during the preparation (54.1%) and implementation (83.7%) phases. The most common action targets were characteristics of individuals (38.5%), implementation process (36.5%), and characteristics of the inner setting (21.2%). Implementation outcomes that were expected to be impacted included fidelity (64.9%), acceptability (54.1%), feasibility (29.7%), and adoption (27.0%).

**Implications for D&I Research:** This study provides the first comprehensive description of the implementation strategies that were used during a school-based efficacy trial and provides an example of a retrospective approach that may be feasible for other intervention research teams. Educational settings are a unique context that warrant attention throughout all phases of translational research (i.e., efficacy, effectiveness, and implementation). Explicating implementation strategies sooner in this process could lead to better selection, refinement, and testing of strategies in future studies.

**Primary Funding Source:** National Institute of Mental Health, Institute of Education Sciences

### S-93 Acceptability of implementation strategies in a hybrid type 3 randomized trial of universal prevention programs in rural k-12 schools

#### Hannah Calvert, Nate Anderson, Tate Castleton, Teri Lewis, Ashley Havlicak, Carl Siebert, Lindsey Turner

##### Boise State University, Boise, ID, USA

###### **Correspondence:** Hannah Calvert (hannahcalvert898@boisestate.edu)

**Background:** Schools in rural areas often have limited access to resources for implementation of evidence-based programs. Positive Behavioral Interventions & Supports (PBIS) is an evidence-based framework for school-based universal prevention of problem behaviors. Achieving successful PBIS implementation outcomes requires time, capacity, and expertise. Many rural schools lack capacity for implementation (e.g., funding, staffing expertise), and have fewer external resources for implementation support. The RK-12 project is a hybrid type 3 implementation-effectiveness trial in 40 schools, with 20 schools receiving a package of implementation support strategies (external coaching, online resources, virtual learning community), and 20 receiving basic PBIS training without implementation strategies. This study explored perceptions of acceptability of these strategies

**Methods:** At the end of the first year of PBIS implementation (spring 2020), we recruited the principal and coach from each school’s PBIS leadership team for individual 30-minute interviews. Interviews were conducted with 70 individuals (32/40 schools had interviews with both the PBIS coach and administrator). Interviews were recorded and transcribed verbatim. One question addressed overall perceptions of implementation progress before the COVID-19 shutdown, and those in intervention schools were asked about acceptability of the implementation support strategies. The research received Institutional Review Board approval.

**Findings:** Individuals in intervention schools more frequently described a positive implementation process (versus a negative or neutral process) than control schools. Agreement between school dyads was relatively low (50%); administrators more frequently painted a positive picture of implementation than PBIS coaches at the same school. Almost all individuals at intervention schools reported that the PBIS team’s relationship with the external coach was extremely important to their implementation success. Many rated the in-person coaching as the most valuable component of the implementation support, but remote coaching was also helpful. Aspects of the implementation process facilitated by external coaches included accountability for having regular PBIS team meetings, and increased preparedness (having progress reports and action items) for those meetings. Participants emphasized that the objective perspective of an outside expert carried weight and helped their PBIS team stay focused.

**Implications for D&I Research:** External coaching from a content expert is viewed as a critical implementation support strategy for successful implementation of PBIS in rural schools.

**Primary Funding Source:** National Institute of Justice

### S-94 Effects of the COVID-19 pandemic on implementation of keep it up! at community-based organizations

#### Justin Jones^1^, Nanette Benbow^2^, Justin Smith^2^, Dennis Li^2^

##### ^1^Northwestern University, Chicago, IL, USA; ^2^Northwestern University Feinberg School of Medicine, Chicago, IL, USA

###### **Correspondence:** Justin Jones (justin.jones@northwestern.edu)

**Background:**

Keep It Up! 3.0 (KIU) is a Type III Hybrid Implementation-Effectiveness trial. KIU is an effective eHealth HIV prevention intervention for young men who have sex with men (YMSM). This study compares two pragmatic implementation strategies for KIU – centralized direct-to-consumer marketing versus decentralized delivery via community-based organizations (CBOs). Thirteen agencies in the CBO strategy began implementation in January–February 2020. The COVID-19 pandemic caused severe disruptions in their service delivery beginning in mid-March. We elucidate how the COVID-19 pandemic has affected CBOs’ implementation and their strategies to address the concomitant challenges which have arisen.

**Methods:**

Qualitative analysis, using a repetition-based narrative strategy, was conducted on CBO applications submitted in response to a request for proposals to implement KIU (n=13), field notes from one-on-one conversations with sites (n=13), and a recorded all-site meeting. Quantitative data was used to confirm themes derived from qualitative analysis and was obtained from quarterly monitoring reports supplied by CBOs (n=13), a survey administered following COVID-related closures (n=12), and enrollment data for KIU.

**Findings:**

The inability to meet with participants in-person due to COVID-related closures has thwarted HIV testing (key to KIU recruitment) and traditional outreach efforts. Persistent challenges facing many potential participants (e.g., low SES) have been exacerbated, and new challenges have emerged (e.g., closure of colleges). The continually changing landscape has necessitated the writing and rewriting of protocols for the provision of services. The disruption to in-person interactions caused other structural deficits such as reduced connections among CBO community partners and the ability to produce traditional media for recruitment. In response, CBO sites have begun to shift to at-home HIV testing; updated their incentive structures; increased recruitment via social media; and reinvested in relationship development with community partners.

**Implications for D&I Research:**

Future research is needed on (1) the effective use of online and other remote means of recruitment geared towards YMSM, and younger populations in general; (2) how pre-crisis cosmopolitanism predicts success in-crisis and how in-crisis efforts towards improving cosmopolitanism influence outcomes; and (3) how the stressors of crisis affect both implementers and the needs and resources of those served.

**Primary Funding Source:** National Institutes of Health

### S-95 The implications of COVID-19 on cardiac rehabilitation: The need for theory-driven, telehealth-enhanced, non-traditional models

#### Andrea T. Duran^1^, Ian Kronish^1^, Steven J. Keteyian^2^, Siqin Ye^1^, Kimberly Stavrolakes^3^, Harry West^4^, Nathalie Moise^1^

##### ^1^Columbia University Irving Medical Center, New York, NY, USA; ^2^Henry Ford Hospital, Detroit, MI, USA; ^3^New York Presbyterian Hospital, New York, NY, USA; ^4^Columbia University, New York, NY, USA

###### **Correspondence:** Andrea T. Duran (atd2127@cumc.columbia.edu)

**Background:** Using a theory-driven approach, we sought to determine the best model for cardiac rehabilitation (CR) delivery in the post-COVID-19 era of remote clinical care.

**Methods:** When clinic-based CR services ceased (March-May 2020), we conducted key informant interviews as part of a New York Presbyterian Hospital (NYPH) quality improvement initiative via Zoom/phone-call with CR supervisors (n=3) at major academic medical centers (New York, California, Michigan) and staff members (n=4)/health system leaders (n=2) affiliated with NYPH. Utilizing the Theoretical Domains Framework and the Consolidated Framework for Implementation Research, we assessed organizational-, provider-, and patient-level determinants of clinic- and home-based CR implementation during COVID-19, before eliciting suggestions on how best to design a remotely-delivered CR model.

**Findings:** For clinic-based CR, external policies (e.g., social-distancing), infrastructure/available resources (e.g., limited space), emotion (e.g., patient discomfort/fear of in-hospital services; provider redeployment/burnout), and relative priority (e.g., safety over clinic-based CR) were key determinants of implementation during COVID-19. For home-based CR, external policies (e.g., reimbursement), available resources (e.g., staff capacity; telehealth services/devices; exercise equipment), cost (e.g., limited hospital budget), knowledge/skills/beliefs about capabilities (e.g., unfamiliarity with home-based CR/telemedicine; usability; language/communication), decision processes (e.g., triaging patients), and beliefs about consequences (e.g., patient safety) were key barriers; facilitators included collaborating with CR/telehealth champions/opinion leaders, engaging leadership and leveraging existing EHR/telemedicine infrastructure, and intervention adaptability.

Informed by our results, we co-designed a telehealth-enhanced hybrid CR model with the potential to uphold CDC COVID-19 guidelines, align with NYPH telehealth initiatives, promote patient-provider communication, and permit reimbursement. The hybrid design combines home-based CR (e.g., remote exercise monitoring) with NYPH’s existing clinic-based CR and EHR/MyChart (e.g., video visits) infrastructure to offer 24 CR sessions (4 clinic-based, 20 home-based) over 12 weeks. This model is currently being pilot tested among low-risk cardiac patients attending a NYPH CR clinic.

**Implications for D&I Research:** This is the first study to employ theoretical frameworks to identify multi-level determinants of both clinic- and home-based CR implementation during COVID-19. Different barriers emerged for each CR model, supporting the need for a telehealth-enhanced hybrid CR program. Future research will provide vital knowledge on the effectiveness, feasibility, acceptability, and appropriateness of this hybrid model in the post-COVID era.

**Primary Funding Source:** National Institutes of Health

### S-96 Feasibility of community prevention organizations in promoting prescription medication disposal

#### Vaishnavi Tata^1^, Tamara Al Rawwad^1^, Danielle Campbell^1^, Matthew Wanat^1^, James Douglas Thornton^2^

##### ^1^University of Houston College of Pharmacy, Houston, TX, USA; ^2^University of Houston College of Pharmacy, The Prescription Drug Misuse Education and Research(PREMIER) Center, Houston, TX, USA

###### **Correspondence:** Vaishnavi Tata (vtata@central.uh.edu)

**Background:**

Community prevention organizations (CPOs), primarily Substance Abuse Prevention and Treatment Block Grant funded groups, serve public health regions throughout Texas. The objective of this study was to assess the feasibility of providing Single Use Disposal Systems (SUDS) through CPOs as part of a state-wide effort to promote the safe disposal of prescription medications and mitigate prescription drug misuse.

**Methods:**

This sequential explanatory study design included 47 CPOs, each covering a mutually exclusive geographic area within Texas. The quantitative arm of the study assessed the number of SUDS each organization received and distributed. Organizations were categorized as high-, medium-, or low-performing based on the proportion of SUDS distributed. The qualitative arm of the study involved semi-structured interviews to assess the process of distributing SUDS. Stratified random sampling was used to select five organizations from each performance strata to participate in a semi-structured interview. The interview guide was developed in accordance to Bowen’s framework and all constructs were represented. The interviews were conducted by two separate members of the research team. Thematic analysis was used to analyze the data from the semi-structured interviews. The coding of the transcripts was done by two separate members of the research team and any discrepancies were arbitrated by a third member.

**Findings:**

The thematic analysis identified 58 codes that were then grouped into 10 themes. Partnerships with local institutions and ability to distribute at community events were universal facilitators of the CPOs’ feasibility in providing SUDS to their community. The high and medium performing organizations emphasized education promotion efforts as a strategy to increase utilization of SUDS among end users. Low performing coalitions treated the SUDS as supplemental to their overall goals.

**Implications for D&I Research:**

CPOs have unparalleled access to community events, local institutions, and the general population they serve. For this reason, they have the potential to be active facilitators in implementing and disseminating health interventions. These findings are not only reported back to the funding agency but disseminated to participating organizations via virtual town halls. The next step within this project is to link CPO distribution to end user disposal activity.

**Primary Funding Source:** Substance Abuse and Mental Health Services Administration (SAMHSA)

### S-97 Understanding the influence of external and internal characteristics on the penetration of naloxone delivery from syringe service programs in the United States

#### Barrot H Lambdin^1^, Lynn Wenger^1^, Ricky Bluthenthal^2^, Bryan Garner^3^, Paul LaKosky^4^, Savannah O'Neill^5^, Alex Kral^1^

##### ^1^RTI International, San Francisco, CA, USA; ^2^University of Southern California, Los Angeles, CA, USA; ^3^RTI International, Research Triangle Park, NC, USA; ^4^North American Syringe Exchange Network, Tacoma, WA, USA; ^5^Harm Reduction Coalition, Oakland, CA, USA

###### **Correspondence:** Barrot H Lambdin (blambdin@rti.org)

**Background:** Syringe service programs (SSPs) have pioneered implementation efforts for naloxone distribution. Naloxone is an opioid antagonist that reverses opioid overdoses, and SSPs are ideal venues for naloxone distribution, with staff who excel in providing culturally appropriate services for people who use drugs (PWUD) and delivery systems designed to reach PWUD where they are. We assessed which factors from SSPs’ external and internal context were associated with penetration of naloxone distribution in the United States.

**Methods:** We surveyed all SSPs in the United States known to the North American Syringe Exchange Network in 2019. Out of the 342 known SSPs operating, 266 (78%) responded to the online survey. We utilized linear regression to assess which factors were associated with the number of naloxone doses distributed and people receiving naloxone, adjusting for opioid overdose deaths in the region. Both outcomes were log transformed to achieve a normal distribution.

**Findings:** SSPs reported distributing 710,232 naloxone doses to 230,506 people in the prior year. Regarding the external context, SSPs located in areas with high levels of community support had a 98% (p=0.011) higher level of naloxone distribution and 73% (p=0.022) higher number of people receiving naloxone. Regarding the internal context, SSPs with proactive refill systems (105% higher compared to passive refill systems; p=0.013), greater number of naloxone distribution days (12% higher for each additional day; p<0.001) and older programs (12% higher for each year older; p<0.001) were associated with higher levels of naloxone distribution. Also, a greater number of naloxone distribution days (9% higher for each additional day; p<0.001) and older programs (12% higher for each year older; p<0.001) were associated with a higher number of people receiving naloxone.

**Implications for D&I Research:** We identified external and internal characteristics of SSPs that were associated with greater penetration of naloxone delivery. Ensuring SSPs are adequately resourced to build community-support for services and develop service delivery models that maximize penetration of naloxone is critical to address the nation’s opioid overdose crisis.

**Primary Funding Source:** National Institutes of Health

### S-98 Evaluating statewide efforts to prevent adolescent substance use in Washington using the RE-AIM framework

#### Gitanjali Shrestha^1^, Brittany Cooper^1^, Laura Hill^1^, Elizabeth Weybright^2^

##### ^1^Human Development, Washington State University, Pullman, WA, USA; ^2^Washington State University, Pullman, WA, USA

###### **Correspondence:** Gitanjali Shrestha (gshrestha@wsu.edu)

**Background:** A priority for the field of prevention science is to identify a model for scaling up evidence-based programs (EBPs) that takes into account the dynamic and diverse prevention needs of communities. The WA State Community Prevention and Wellness Initiative (CPWI) is a community coalition-based model where local coalitions use a data-informed process to select and implement adolescent substance use prevention EBPs while receiving state funding and technical assistance. CPWI started in 2011 with a cohort of 19 communities and currently there are six cohorts with over 80 communities. We used the RE-AIM framework, which posits the public health impact of an initiative is a function of multiple dimensions, to examine the impact of CPWI on four RE-AIM dimensions: Effectiveness, Adoption, Implementation, and Maintenance. We examined impact of CPWI Cohorts 1-4, and aggregated data at the cohort level to determine the higher (macro) level impact of CPWI.

**Methods:** We used data from the CPWI Process Evaluation Survey, the CPWI Impact Over Time Evaluation Report, and state-level administrative database. We measured the RE-AIM dimensions as follows: 1) Effectiveness: proportion of outcomes where CPWI communities showed improvement from baseline to posttest; 2) Adoption: proportion of respondents who agreed their CPWI coalition has collaborative relationships and community support; 3) Implementation: proportion of EBPs implemented in the communities; 4) Maintenance: proportion of respondents seeking additional non-CPWI funding to implement CPWI activities.

**Findings:** The four CPWI cohorts included in the evaluation performed very well across all four dimensions with levels of Effectiveness, Adoption, Implementation, and Maintenance ranging from medium to high within and across cohorts. Three of these four cohorts scored high on Implementation. One of four cohorts scored high on Effectiveness and Maintenance.

**Implications for D&I Research:** Results indicate CPWI has the potential to positively affect public health outcomes by addressing the dynamic and diverse needs of communities. This presentation will address results of the RE-AIM evaluation and highlight the RE-AIM evaluation process including the generation of evaluation questions, selection of data from existing datasets, and lessons learned.

**Primary Funding Source:** Washington State Health Care Authority/Division of Behavioral Health and Recovery

### S-99 Optimizing naloxone distribution to prevent opioid overdose fatalities: Results from piloting the systems analysis and improvement approach within syringe service programs

#### Barrot H Lambdin^1^, Alex Kral^1^, **Anjuli Wagner**^2^, Lynn Wenger^1^, Kenneth Sherr^2^

##### ^1^RTI International, San Francisco, CA, USA; ^2^University of Washington, Seattle, WA, USA

###### **Correspondence:** Anjuli Wagner (anjuliw@uw.edu)

**Background:** Opioid overdose fatalities are preventable with the timely administration of naloxone, an opioid antagonist, during an opioid overdose event. Syringe service programs (SSP) have pioneered the implementation of naloxone distribution for people with a high risk of observing and/or experiencing an opioid overdose. We adapted and piloted an implementation strategy - the systems analysis and improvement approach (SAIA) – with the goal of improving the penetration of naloxone distribution from SSPs.

**Methods:** Twelve staff from two SSPs piloted SAIA for 6 months. SAIA includes (1) *cascade analysis* to identify gaps in the naloxone delivery cascade, (2) *flow mapping* to identify areas of attrition and brainstorm concepts for improvement, and (3) *continuous quality improvement* to test programmatic adaptations that address those gaps and assess the impact of these adaptations on the delivery cascade. We conducted an interrupted time-series study, using 52 weeks and 26 weeks of data before and after SAIA implementation, respectively. Prais-Winsten linear regression, accounting for serial correlation and adjusting for SSP participant volume and seasonality, was used to assess for changes in the average weekly number of people receiving naloxone and number of naloxone doses distributed.

**Findings:** Over the course of the study, 4,715 doses of naloxone were distributed to 1,883 participants. SSPs prioritized efforts to improve data collection procedures, proactively screen and identify naloxone-naïve participants and streamline naloxone refill systems. After SAIA implementation, we observed significant increases in the weekly average number of people receiving naloxone (average of 6 people per week received naloxone pre-SAIA verses 62 people per week received naloxone post-SAIA; adjusted Mean Difference (aMD) = 51; 95% CI: 45-57; p<0.001). We also observed a significant increase in the weekly average number of naloxone doses distributed (18 doses per week pre-SAIA vs 151 doses per week post-SAIA; aMD = 117; 95% CI: 90-143; p<0.001).

**Implications for D&I Research:** Our pilot suggested that SAIA has strong potential for improving penetration of naloxone distribution from SSPs. These findings are encouraging given the worsening opioid overdose crisis in the United States and support testing the SAIA in a large-scale randomized trial of SSPs.

**Primary Funding Source:** National Institutes of Health

## Promoting Health Equity and Eliminating Disparities

### S-100 Implementation preparation barriers and facilitators can influence modification type and frequency to a culturally competent care intervention

#### Jefferson Uriarte^1^, Naomi Anderson^1^, Henna McCoy^1^, Cindy Berumen^1^, Michelle Shumate^2^, Juan Carlos Caicedo^1^, **Elisa Gordon**^1^

##### ^1^Northwestern University Feinberg School of Medicine, Chicago, IL, USA; ^2^Northwestern University, Evanston, IL, USA

###### **Correspondence:** Elisa Gordon (e-gordon@northwestern.edu)

**Background:** Modifications to interventions are important to examine because they may interfere with fidelity and outcomes. Little is known about how organizational factors contribute to different types of modifications to complex interventions during the implementation phase. This study examined the types and characteristics of modifications made to the Hispanic Kidney Transplant Program (HKTP), a culturally competent care intervention, and describes how perceived institutional barriers and facilitators expressed during the implementation preparation phase may have enabled such modifications.

**Methods:** The HKTP intervention was implemented at two U.S. kidney transplant programs. Participants included transplant stakeholders: transplant physicians, administrators, and staff. In-depth qualitative interviews, quarterly learning collaborative discussions, and biweekly telephone meetings were conducted during the implementation preparation phase (2016) and implementation phase (2017) to assess perceived barriers and facilitators to implementing HKTP components and to identify modifications made to the intervention. The Consolidated Framework for Implementation Research (CFIR) guided interview design; Stirman’s Framework for Modifications and Adaptations guided modification classification.

**Findings:** 80 stakeholders participated across sites (2016: 51, 2017: 29). Overall, 51 modifications were made to the intervention (Site A: 18, Site B: 33). Sites differed by proportions of modification types: delaying/skipping (Site A: 50%, Site B: 34%), adding (Site A: 17%, Site B: 34%), tweaking (Site A: 11%, Site B: 16%), and substituting (Site A: 17%, Site B: 6%). Across sites, most modifications were made by the transplant team, followed by individuals, and institution (Site A: 66%, 17%, 17%; Site B: 63%, 19%, 12%), respectively. CFIR inner setting factors (e.g., infrastructure, team culture, complexity) helped explain the types of modifications made. The organization with more complex infrastructures (Site A) reported more skipping of intervention components, while the organization with an egalitarian team culture (Site B) reported more additions to the intervention.

**Implications for D&I Research:** Organizations implementing a complex culturally competent care intervention performed numerous modifications in the first year of implementation. Perceived intervention barriers and facilitators in the preparation phase may help explain why certain modifications occurred. Identifying factors contributing to modifications may help institutions prepare to implement interventions in a more deliberate manner while maximizing intervention fidelity.

**Primary Funding Source:** National Institutes of Health

### S-101 Using organizational input to adapt an evidence-based intervention for implementation in rural communities

#### Scherezade Mama^1^, Kathryn Schmitz^2^, Laura Rogers^3^

##### ^1^The University of Texas MD Anderson Cancer Center, Houston, TX, USA; ^2^Public Health Science, Penn State College of Medicine, Hershey, PA, USA; ^3^University of Alabama at Birmingham, Birmingham, AL, USA

###### **Correspondence:** Scherezade Mama (skmama@mdanderson.org)

**Background:** Rural cancer survivors are more likely to report high psychosocial distress and physical inactivity than urban cancer survivors. Evidence-based interventions to reduce distress and increase physical activity (PA) in cancer survivors were developed in urban or clinical settings and have previously failed when implemented in rural community settings. Thus, there is a clear need to adapt interventions to meet rural cancer survivors’ unique needs, that can be implemented in rural settings with limited resources.

**Methods:** This study used a staged community-engaged approach to adapt an evidence-based PA intervention to reduce psychosocial distress in rural cancer survivors. Stage 1 focused on setting-level adaptations. Community organizations (*N*=419) that serve rural cancer survivors, including cancer support groups, churches, community centers, YMCAs, local health centers, and non-profits, were initially contacted to complete a web-based or mailed survey. The Consolidated Framework for Implementation Research (CFIR) was used to guide survey development. Survey questions covered key attributes that may influence successful implementation of a physical activity intervention, including inner and outer setting characteristics, characteristics of individuals within organizations, and setting-level processes that impact implementation.

**Findings:** Ninety-six individuals representing 93 unique organizations completed the survey. Most organizations (55.2%) were located in a mostly rural or completely rural area, and 68.4% were physically located in the community their organization served. Most (71.9%) reported that opinion leaders within their organization consider PA programs to be beneficial or important, but only 16.0% strongly agreed that they would be able to successfully implement a PA program. Although 56.4% of participants felt that leadership would provide support for a new PA program to meet the needs of individuals served by the organization, 60.6% reported that changes would be needed to support the new program, and only 42.4% felt their organization was ready to make those changes. Organizations reported several barriers to implementing a new PA program, including cost (73.2%), the physical structure of the organization or space (64.9%), and conflicting priorities within the organization (33.3%).

**Implications for D&I Research:** Findings from this stage enforce the need for organization and stakeholder input to aid translation of evidence-based interventions into sustained practice within rural communities.

**Primary Funding Source:** National Institutes of Health

### S-102 The impact of joining an accountable care organization on the uptake of cancer screening practices in rural primary care

#### Heather Nelson-Brantley, Edward Ellerbeck, Allen Greiner, Christie Befort

##### University of Kansas Medical Center, Kansas City, KS, USA

###### **Correspondence:** Heather Nelson-Brantley (hnelson-brantley@kumc.edu)

**Background:** Cancer screening is recommended by the CDC as an evidence-based approach for early detection of many cancers. Yet, screening disparities among rural communities in the U.S. persist, with rates 15-30% lower than among non-rural populations. Many resource-challenged rural clinics are now joining Accountable Care Organizations (ACOs) to take advantage of alternative payment models that focus on shared savings and financial incentives for achieving quality targets. Yet, it is unclear what impact joining an ACO has on the uptake of cancer screening practices. The purpose of this study was to describe how cancer screening practices in rural primary care clinics were affected by joining an ACO.

**Methods:** We used a multiple case study design. Rural primary care clinics, defined as RUCA codes 6-9, in one ACO in the Midwestern U.S. were invited to participate. Data were collected from focus group interviews, cancer screening records, and workflow process mapping. Interviews, guided by the Consolidated Framework for Implementation Research, were conducted with providers, staff, and administrators. Data were cross-analyzed by clinic to identify common themes and clinic-specific contextual barriers and adaptations.

**Findings:** Eight clinics participated in the study. With integration into the ACO, clinics adopted four new strategies to support cancer screening: care gap lists, huddle sheets, screening via annual wellness visits, and information spread. Care gap lists provided by the ACO served as the first population-based reminder system and ACO promotion of wellness visits created the first opportunity for clinics to focus exclusively on addressing gaps such as cancer screening. All clinics used both visit-based and population-based cancer screening approaches. However, workflows varied widely across clinics. Clinics with higher cancer screening rates engaged nursing staff at higher levels and utilized their electronic health records to a higher degree compared to clinics with lower cancer screening.

**Implications for D&I Research:** Our study found considerable variation in cancer screening workflows and adaptations across rural primary care clinics within one Midwestern ACO. Future studies that compare the effectiveness of different implementation strategies and adaptations can advance the field vertically by improving the uptake of cancer screening practices in rural primary care, in turn, decreasing cancer-related health disparities among rural populations.

**Primary Funding Source:** National Institutes of Health

### S-103 Understanding de-implementation and adaptation of evidence-based interventions over time in the context of changing screening guidelines: Trust and mistrust as foundational determinants of health equity in implementation science

#### Rachel Shelton^1^, Laura Brotzman^1^, Detric Johnson^2^, Savannah Alexander^3^, Debbie Erwin^2^

##### ^1^Columbia University Mailman School of Public Health, New York, NY, USA; ^2^Roswell Park Cancer Insittute, Buffalo, NY, USA; ^3^Columbia University, School of Public Health, New York, NY, USA

###### **Correspondence:** Rachel Shelton (rs3108@cumc.columbia.edu)

**Background:** Research is needed to understand adaptation of programs to reflect changing scientific guidelines, including factors influencing the de-implementation of messaging or components that reflect guidelines or practices no longer recommended or with limited benefit according to new scientific evidence. This is particularly important to understand among African American communities that may experience higher levels of mistrust of providers and healthcare, rooted in a system of historical and ongoing structural discrimination in the U.S. This research seeks to address this gap by investigating barriers and facilitators to the adaptation of programs reflecting new scientific guidelines for breast/cervical cancer screening.

**Methods:** We conducted a convergent, mixed-methods design among The National Witness Project (NWP), a nationally implemented evidence-based Lay Health Advisor (LHA) program for breast/cervical cancer screening among African American women. Surveys were conducted among 201 Project Directors and LHAs representing 14 NWP sites nationally; in-depth interviews were conducted among 14 Project Directors to contextualize findings.

**Findings:** Trust and mistrust were important themes that arose in quantitative and qualitative data. Quantitative data indicated that only 57% of women agreed that there is high trust of medical organizations/providers in their community and 64% of women reported trusting the new breast/cervical cancer guidelines. Common concerns about adapting to new guidelines identified through the quantitative and qualitative data included: 1) perceptions that new guidelines misalign with personal values and beliefs of African American women; 2) mistrust of guidelines, providers, medical organizations; 3) confusion about inconsistent guidelines and concern they are based on studies that don't reflect the experiences of African American women (who have more aggressive tumors at younger ages); and 4) belief that breast self exam (BSE) is an empowerment tool for African American women and should be included to promote awareness, given many women discovered lumps/cancer through BSE.

**Implications for D&I Research:** Findings highlight trust and mistrust as foundational, structural factors that shape social determinants of health among African Americans, and that: 1) have critical implications for health equity in implementation science frameworks; and 2) provide context as to why new screening guidelines may not be fully embraced in this community.

**Primary Funding Source:** American Cancer Society

### S-104 Identifying technologies of power: A critical implementation praxis to advance health equity using the exploration, preparation, implementation, sustainment (EPIS) framework

#### Megan Stanton^1^, Samira Ali^2^

##### ^1^Eastern Connecticut State University, Willimantic, CT, USA; ^2^University of Houston, Houston, TX, USA

###### **Correspondence:** Megan Stanton (stantonmeg@easternct.edu)

**Background:** Implementation Science (IS) has begun to examine how the field can more intentionally promote health equity through, for example, centering communities experiencing health inequity in IS research and strengthening the science of adaptation. Lacking is a conceptual framework to analyze how power is generated and distributed through practical implementation processes and how this power can dismantle and/or reproduce health inequity through intervention implementation. This presentation will posit a conceptual framework which 1) identifies specific technologies of power which work through implementation, and 2) applies this power typology to articulate a critical implementation praxis to advance health equity using the Exploration, Preparation, Implementation, Sustainment (EPIS) framework.

**Methods:** The conceptual framework draws on the authors’ extensive work as researchers and capacity building consultants working with community-based organizations implementing evidence-based interventions and integrates literature review, the EPIS framework and ‘notes from the field’.

**Findings:** The authors identify three technologies of power working through implementation; 1) ***discursive power*** is enacted through defining health related problem to be targeted by intervention implementation, as well as through health narratives that emerge through implementation; 2) ***epistemic power*** influences whose knowledge is valued in decision-making and is recreated through knowledge generation; and 3) ***material power*** is created through resource distribution and patterns of access to health resources and acquisition of health benefits provided by the intervention. Decisions across all phases and related to all factors of EPIS influence how these forms of power striate through intervention implementation and ultimately affect health equity outcomes.

**Implications for D&I Research:** Health inequity is fundamentally about structures of power. This conceptual framework culminates in a set of concrete praxis questions for researchers and practitioners to interrogate power in implementation processes. This can result in meaningful changes to implementation planning and execution which enhance the health equity potential of evidence based interventions in community settings.

### S-105 Coach as ethnographer: Enhancing mixed-method implementation studies with participant-observation

#### Daniel Shattuck

##### Behavioral Health Research Center of the Southwest, Pacific Institute for Research and Evaluation, Albuquerque, NM, USA

###### **Correspondence:** Daniel Shattuck (dshattuck@pire.org)

**Background:** Short-term ethnography has grown in popularity within health services research because the methodological, practical, and analytical points of entry facilitate rapid application of emergent knowledge in urgent contexts. Ethnography also provides access to quotidian experiences and the multiple interpretations of reality within a given setting. Interventions, especially those aimed at ameliorating disparities for stigmatized populations, often depend on successfully navigating the confluence of individual-, interpersonal-, and institutional-level factors. Using the case of implementation coaches in the “Implementing School Nursing Strategies to Reduce LGBTQ+ Adolescent Suicide” (RLAS)—a study focused on the uptake and sustainment of six school-based LGBTQ+ supportive practices—this presentation explores the ways coaches can be effectively leveraged as ethnographers within a mixed-method research framework.

**Methods:** Researchers conducted and analyzed 88 semi-structured interviews and 33 focus groups spanning two cycles of annual data collection with school-based implementation leads and implementation resource teams across 19 sites, in addition to individual and small group interviews with six coaches in the third year of implementation. We also examined qualitative data derived from three years of weekly debriefs with implementation coaches, coach activity logs, school action plans, school self-assessments, and annual coaching summary reports.

**Findings:** Coaches’ capacity for ethnography emerged as a critical component of how the research team could make sense of site-level activities and engage in a continual, iterative, and real-time process of knowledge production and action. Coaches 1) served as change agents within sites; 2) improved quality of data collection through intensive rapport-building; 3) engaged in de facto participant observation; and 3) complemented, grounded, and triangulated data.

**Implications for D&I Research:** Strategic use of ethnographic methods can enhance implementation science research by providing nuanced attention to factors impacting implementation. Emphasis on the “invisible” labor that goes into making interventions work within a given setting, is particularly important for interventions aimed at stigmatized populations, like LGBTQ+ youth. In these milieus, attitudes and motivations at the individual level about the target beneficiary population—homophobia or transphobia—are intertwined with attitudes about interventions. Coaches, as quintessential participant observers, are well-positioned to perform the dual role of implementation practitioner and ethnographic researcher.

**Primary Funding Source:** National Institutes of Health

### S-106 Shifting to remote breastfeeding counseling for low-income minority women during COVID-19: A rapid process evaluation

#### Elizabeth Rhodes^1^, Helen Wilde LaPlant^2^, Grace Damio^2^, Walter Trymbulak^3^, Carrianne Crummett^3^, Rebecca Surprenant^3^, Nafeesa Abuwala^4^, Mahrukh Zahid^1^, Rafael Pérez-Escamilla^1^

##### ^1^Yale School of Public Health, New Haven, CT, USA; ^2^Hispanic Health Council, Hartford, CT, USA; ^3^Saint Francis Hospital and Medical Center, Trinity Health Of New England, Hartford, CT, USA; ^4^Yale School of Medicine, New Haven, CT, USA

###### **Correspondence:** Elizabeth Rhodes (elizabeth.rhodes@yale.edu)

**Background:** The Breastfeeding Heritage and Pride Program (BHP) provides evidence-based breastfeeding peer counseling to low-income minority mothers in Connecticut and Massachusetts. With the emergence of COVID-19, BHP shifted from delivering services in hospitals and participants’ homes to operating remotely. We aimed to systematically document adaptations to BHP and understand the feasibility, acceptability, and appropriateness of remote services.

**Methods:** We conducted interviews with implementation staff (program managers, peer counselors (PCs)) (n=12) and participants (n=24). We used semi-structured interview guides informed by the Framework for Reporting Adaptations and Modifications-Enhanced (FRAME) and Proctor’s taxonomy of implementation outcomes. Each interview was conducted in the individual’s preferred language (English or Spanish), audio-recorded, and transcribed verbatim. Data were analyzed using a framework-guided rapid analysis approach to provide timely results to implementation staff.

**Findings:** Adaptations to BHP included: recruitment via phone calls; replacement of in-person visits with videoconferencing or phone calls; PCs working from home with flexible hours; and move from paper to electronic forms for collecting data for monitoring. The adaptation process was facilitated by planning and support from program managers (e.g., provision of laptops and videoconferencing training for PCs). PCs were able to continue recruitment remotely, but some mothers did not respond to “cold calls.” PCs liked that their flexible schedules made them more available to participants and electronic data collection was efficient. However, PCs felt that virtual visits were less appropriate for building relationships, helping with certain breastfeeding problems (e.g., latching difficulties), and identifying barriers to breastfeeding (e.g., limited familial support at home). Some participants wanted support via videoconferencing (e.g., when experiencing breastfeeding challenges), while others preferred phone calls because of convenience. Although most participants appreciated remote support and expressed interest in using mobile technology after COVID-19, some participants desired future in-person visits.

**Implications for D&I Research:** The shift to remote breastfeeding counseling was well-planned and enabled service continuity for a low-income minority population amidst a public health emergency. Findings indicate that delivering services remotely has been feasible and acceptable but not appropriate for all aspects of counseling. Research is needed to design a telehealth model for breastfeeding counseling that can complement in-person services and evaluate its usability, effectiveness, and cost-effectiveness.

**Primary Funding Source:** National Institutes of Health

### S-107 Clinician and health care staff perspectives on potential disparities introduced by the rapid implementation of telehealth services during COVID-19

#### Phoutdavone Phimpasone-Brady, Amber Sieja, Rita Lee, Joanne Chiao, Mark Earnest, Samantha Farro, Danielle Loeb, Laura Macke, Hillary Lum, Christopher Schifeling, Amy Huebschmann

##### University of Colorado School of Medicine, Aurora, CO, USA

###### **Correspondence:** Amy Huebschmann (amy.huebschmann@ucdenver.edu)

**Background:** COVID-19 has rapidly altered the delivery of healthcare. The rapid implementation of telehealth due to COVID-19 may have unintentionally worsened health disparities. The purpose of this study was to assess the rapid telehealth implementation experiences of clinicians and staff in primary care and Psychiatry, including their perspectives on potential disparities that occurred in this transition, and recommended strategies to improve telehealth implementation.

**Methods:** Office managers at three University of Colorado Health (UCHealth) clinics (Geriatrics, Internal Medicine, Psychiatry) e-mailed invitations to all clinic staff and clinicians. Experiences with telehealth care delivery were assessed through open-ended questions and Likert-scale responses, including concerns about disparities introduced and recommendations to improve telehealth implementation. Data were analyzed as descriptive statistics and rapid thematic analysis of qualitative responses.

**Findings:** Respondents (n = 147) were distributed across these specialties: 14% Geriatrics, 66% Internal Medicine, and 19% Psychiatry. Response rate was 57%. Prior to COVID-19, 77% of respondents had never delivered telehealth services. During the COVID-19 "stay-at-home/safer-at-home" period, 78% of respondents conducted at least 50% of clinical services by telehealth. Among respondents, 34% agreed with the statement that “rapid telehealth implementation exacerbated disparities”, as compared to 14% who disagreed. In the qualitative responses, perceived disparities were mainly conceptualized as lower access to telehealth video visits, particularly for patients who were older, had Limited English Proficiency, or limited access to the appropriate technology. Participants’ recommendations to improve these disparities commonly mapped to two categories of implementation strategies: 1) ‘change infrastructure’ to give patients access to ‘electronic tablets’ or other necessary technology, and to ensure video telehealth platforms can simultaneously accommodate family members and/or foreign language translators, in addition to the patient; 2) ‘train and educate stakeholders’, framed as providing patients with better guides for accessing video visits, and assistance troubleshooting common obstacles.

**Implications for D&I Research:** Approximately 1/3 of clinicians and staff at the participating UCHealth clinics perceived disparities introduced by the COVID-19 transition to telehealth, particularly challenges with video visit access for certain populations. To avoid perpetuating disparities in treatment access, further research with patients and clinics to co-create equitable telehealth implementation strategies are urgently needed.

### S-108 Organizationally-tailored vs. standard approach for integrating an evidence-based cancer control intervention into African American churches

#### Cheryl Knott^1^, Janice Bowie^2^, Daniel Mullins^3^, Jimmie Slade^4^, Nathaniel Woodard^1^, Barbara Jean Shaneman-Robinson^3^, Leonore Okwara^1^, Maisha Huq^1^, Ralph Williams^5^, Xin He^1^, Chang Chen^6^

##### ^1^University of Maryland, College Park, MD, USA; ^2^Johns Hopkins University, Baltimore, MD, USA; ^3^University of Maryland, Baltimore, MD, USA; ^4^Community Ministry of Prince George's County, Upper Marlboro, MD, USA; ^5^Leggette ETI, Brentwood, MD, USA; ^6^University of Maryland, College Park, COLLEGE PARK, MD, USA

###### **Correspondence:** Cheryl Knott (cholt14@umd.edu)

**Background:** Implementation science research is increasingly focused on optimizing the fit of evidence-based interventions with the organizational setting in which they are delivered. Less is known about how to maximize this fit, particularly in settings outside of healthcare systems.

**Methods:** We report initial findings from a cluster randomized trial that compared a new organizationally-tailored with a standard (core components only) approach to customizing an evidence-based cancer control intervention in 14 African American churches. In the organizationally-tailored group, leaders in African American churches completed a memorandum of understanding where they identified three or more implementation strategies from a menu of 20, and select the planned implementation timeframe. We report on study group differences in the primary outcome of institutionalization of health promotion activities based on pastor surveys conducted at 12 months. The institutionalization measure, created for use in faith-based organizations, included subscales for *organizational structures* for health promotion (e.g., health ministry or wellness team), *organizational processes* for health promotion (e.g., keeping records of health activities; health policy), *organizational resources* for health promotion (e.g., budget, space), and *organizational communication* for health promotion (e.g., health content in church bulletins or sermons).

**Findings:** Churches in the organizationally-tailored group reported significantly greater institutionalization of health promotion activities at 12 months than those in the standard group (health promotion structures M=4.89 [SD=.93] vs. M=3.38 [SD=1.19], p = .01; health promotion processes M=6.11 [SD=1.83] vs. M=4.57 [SD=.79], p = .04; health promotion resources M=6.44 [SD=1.24] vs. M=4.38 [SD=1.60], p = .01; health promotion communication, M=9.78 [SD=2.33] vs. M=6.13 [SD=3.00], p = .02, respectively).

**Implications for D&I Research:** Initial findings suggest that this new approach may be a promising way to maximize intervention-organization fit, implementation, and sustainability. Implications for integrating evidence-based health promotion interventions into limited-resource, non-healthcare community settings such as churches, are discussed.

**Primary Funding Source:** American Cancer Society

### S-109 Medicaid expansion reduces disparities in access and retention in addiction health services

#### Erick Guerrero^1^, Tenie Khachikian^2^, Yinfei Kong^3^, Avelardo Valdez^4^, Christine Grella^5^, Thomas D'Aunno^6^

##### ^1^Research to End Healthcare Disparities Corp, Los Angeles, CA, USA; ^2^University of Chicago, Chicago, IL, USA; ^3^California State University, Fullerton, CA, USA; ^4^University of Southern California, Los Angeles, CA, USA; ^5^University of California, Los Angeles, Los Angeles, CA, USA; ^6^New York University, New York, NY, USA

###### **Correspondence:** Erick Guerrero (erickgcoaching@gmail.com)

**Background**: The Medicaid expansion has provided an opportunity to eliminate racial and ethnic disparities in treatment access and engagement in addiction health services (AHS) in the United States. By implementing public insurance coverage and delivering coordinate mental health and HIV testing, high capacity treatment programs may eliminate disparities in wait time and retention. High capacity programs in this study accept Medicaid payments and have high levels of director leadership and staff readiness for change. In this IRB approved study, we examined the extent to which program capacity leads to higher implementation of coordinated care and in turn increased treatment access and engagement among Latinos and African Americans comparing pre and post-Medicaid expansion.

**Methods**: We analyzed publically-available multi-year data from clients and programs at four points. We analyzed two waves during pre-expansion in 2011 (N=115 programs, n= 11,526 clients) and 2013 (N=111 programs, n= 18,789 clients), and two waves during post-expansion in 2015 (N=106 programs, n= 17,339 clients) and 2017 (N=94 programs, n= 16,191 clients). We relied on two path analyses to test difference between pre and post expansion on days to enter treatment (wait time) and days in treatment (retention), as well as mechanisms of change (coordinated care). We compared two multiple group negative binomial regression models to test race/ethnicity as moderators and coordinated care as mediating mechanisms.

**Findings**: Compared to pre-Medicaid expansion and white clients, Latinos and African Americans reported shorter wait times to enter care in high-capacity programs post-expansion. African Americans’ retention was longer than whites in high-capacity programs post expansion. Additionally, receipt of HIV testing and coordination of mental health services played an indirect role in the relationship between high capacity programs and shorter wait time.

**Implications for D&I Research:** Medicaid expansion played a significant role in eliminating disparities in treatment access and retention in AHS. Program leadership, readiness for change and Medicaid acceptance are important capacity factors to implement coordinated mental health and HIV testing and increase treatment access. Future D&I research on program capacity factors to implement other public health services (COVID-19 testing) should be considered to mitigate the disproportionate impact of the pandemic on Latino and African American communities.

**Primary Funding Source:** National Institutes of Health

### S-110 Addressing mental health treatment access disparities in rural Michigan: The role of community-university partnership in implementation

#### Addie Weaver^1^, Jessica Hahn^2^, Lynne McQuown^3^, Caroline Landry^1^, Amy Kilbourne^1^, Joe Himle^1^

##### ^1^University of Michigan, Ann Arbor, MI, USA; ^2^Trinity Lutheran Church, Hillsdale, MI, USA^3^Jonesville First Presbyterian Church, Jonesville, MI, USA

###### **Correspondence:** Addie Weaver (weaverad@umich.edu)

**Background:** Rural Americans experience mental health needs at rates similar to urban residents; yet, rural residents are significantly less likely to receive any mental health treatment compared to urban peers. This disparity is driven by substantial barriers to care, the most significant of which is persistent shortage of rural mental health providers. Given well-established access challenges, it is imperative for implementation researchers to engage with rural communities to identify non-mental health settings where evidence-based interventions can be delivered to maximize accessibility and acceptability.

**Methods:** Beginning in 2014, we engaged in a community-university partnership within a rural Michigan county to address community-identified mental health needs using evidence-based practices, packaged to be responsive to the rural context. We specifically explored the feasibility and acceptability of mental health service delivery in church settings. We conducted mixed methods studies including: 1) two community surveys with 120 respondents, 2) focus groups and interviews with 33 human service providers, and 3) interviews and focus groups with 21 clergy.

**Findings:** Results suggest depression is a significant concern in this community; yet treatment resources are limited. 25% of 63 congregant survey respondents and 49% of 57 respondents of a survey of food bank service recipients endorsed at least mild to moderate depressive symptoms, whereas thematic analyses of provider focus groups identified a major gap in services for mild to moderate depression. Residents, providers, and clergy were receptive to packaging evidence-based depression treatment for delivery in rural churches, as this aligns with residents’ help-seeking preferences, reduces stigma, and decreases access challenges. Findings indicate the need for clergy training and support, use of technology to facilitate intervention delivery, and packaging the intervention to be non-stigmatizing, tailored for rural experiences and church settings, and inclusive for residents with low literacy.

**Implications for D&I Research:** This community-university partnership led to development and implementation of a computer-assisted cognitive behavioral therapy for depression, Raising Our Spirits Together, tailored for the rural context and church setting, that is currently being piloted. Participatory approaches can advance health equity and offer strategies for innovatively, acceptably, and sustainably packaging and delivering needed care in low resource community settings.

**Primary Funding Source:** National Institutes of Health

### S-111 Promoting community conversations about research to end suicide: Engaging community stakeholders in shaping the pathway to from research to action

#### **Lisa Wexler**^1^, Roberta Moto^2^, Pangaanga Pungowiyi^3^

##### ^1^University of Michigan, Ann Arbor, MI, USA; ^2^Maniilaq Association, Kotzebue, AK, USA; ^3^Kawerak Organization, Nome, AK, USA

###### **Correspondence:** Lisa Wexler (lwexler@umich.edu)

**Background:** Community settings, being a foundational site for determinants of health, are an ideal point of intervention for many disparate health conditions, including mental health and suicide. However, community settings, such as remote Alaska Native villages, when compared to clinical environments, are less hierarchical, more informally structured, and highly dynamic, presenting some challenges to conventional research approaches. Furthermore, implementation at the community level necessitates community members’ championing research evidence to inform decisions at multiple levels and across different community domains (formal and informal); however, studies focused on understanding how, why, and under what conditions community members learn about and use research-based evidence are relatively rare.

**Methods:** PC CARES is a community health intervention that draws upon participatory approaches which prioritize bi-directional exchange and co-creation of knowledge. In collaboration with a local steering committee, research advisory board, and iterative and collaborative co-development, PC CARES uses a train-the-trainers model where local facilitators host a series of learning circles using culturally responsive and evidence-supported curriculum to promote active learning and spark local grassroots efforts to implement suicide prevention measures in rural Alaskan villages.

**Findings:** Local PC CARES facilitators have hosted 73 learning circles with 643 participants between 2015-2020. Facilitators follow curriculum with fidelity (80%) and accuracy (81%). Linked participant surveys show participants report high levels of satisfaction and positive changes in knowledge, beliefs, collaborative relationships, and willingness to take action for suicide prevention in their communities.

**Implications for D&I Research:** On this panel, we will share how PC CARES positions community partners and end-users as primary actors in using research evidence to shape the implementation pathway from research to action in their own communities. In particular, we emphasize how generalizable research evidence has been made useful by our partner communities in ways that align with their priorities and fit within the constraints of their daily lives. Novel approaches to measurement and evaluation for non-prescriptive, locally driven interventions where end users determine how best to utilize research information will also be discussed.

**Primary Funding Source:** National Institutes of Health

### S-112 Methods for engaging and retaining larges samples of community agencies and practitioners

#### Rogerio Pinto^1^, Susan Witte^2^, Prema Filippone^2^

##### ^1^University of Michigan, Ann Arbor, MI, USA; ^2^Columbia University, New York, NY, USA

###### **Correspondence:** Rogerio Pinto (ropinto@umich.edu)

**Background:** Engaging community-based service agencies and health service providers is central to advancing the implementation of evidence-based practices. Nonetheless, “real-world” challenges – large caseloads, time constraints, and limited resources – have hampered recruiting and retaining providers. Meaningful engagement and retention requires participatory approaches in which providers are true partners who participate in a systematized combination of research tasks and procedures.

**Methods:** We present a research-engagement model used in Project ICI, “Interprofessional Collaboration Implementation,” the first longitudinal, mixed methods study aimed to examine the impact of collaboration among providers as they implement evidence-based HIV prevention services in New York City. The research-engagement model was developed based on several studies (both qualitative and quantitative) examining providers’ willingness to become involved in research, as well as rigorous review of community-engaged research literature. The research-engagement model includes four core participatory approaches critical for sustained engagement and successful implementation: 1) creation of an implementation community collaboration board that uses dialectic processes, 2) social gatherings between researchers and community partners, 3) collaborative identification and resolution of recruitment barriers, and 4) sharing resources with the community.

**Findings:** The research-engagement model resulted in successful recruitment and retention of 36 community-based agencies over the 5-year ICI project period. 379 providers representing the 36 agencies participated in the ICI project at baseline, 75% of whom were retained at 12-month follow-up (n=285). Between 12-month (n=285) and 24-month (n-256) follow-up, 90% of the providers were retained. All providers (n=20) selected to participate in qualitative interviews at baseline, 12-month, and 24-month follow-up were retained throughout the project.

**Implications for D&I Research:** Research directives encourage researchers to engage with community partners, yet there is limited implementation research fully describing successful community-engaged practices and participatory approaches. This case illustration provides a concrete research-engagement model for implementation studies. Findings demonstrate the value of community-engaged practices for developing and sustaining partnerships with providers that are essential to identifying and testing feasible, acceptable, and sustainable implementation strategies over time. Additionally, results call attention to the ways in which participatory approaches provide local knowledge that allows for rapid response and adaptation to environmental shifts in policies and priorities.

**Primary Funding Source:** National Institutes of Health

